# Widespread white matter microstructural differences in schizophrenia across 4322 individuals: results from the ENIGMA Schizophrenia DTI Working Group

**DOI:** 10.1038/mp.2017.170

**Published:** 2017-10-17

**Authors:** S Kelly, N Jahanshad, A Zalesky, P Kochunov, I Agartz, C Alloza, O A Andreassen, C Arango, N Banaj, S Bouix, C A Bousman, R M Brouwer, J Bruggemann, J Bustillo, W Cahn, V Calhoun, D Cannon, V Carr, S Catts, J Chen, J-x Chen, X Chen, C Chiapponi, Kl K Cho, V Ciullo, A S Corvin, B Crespo-Facorro, V Cropley, P De Rossi, C M Diaz-Caneja, E W Dickie, S Ehrlich, F-m Fan, J Faskowitz, H Fatouros-Bergman, L Flyckt, J M Ford, J-P Fouche, M Fukunaga, M Gill, D C Glahn, R Gollub, E D Goudzwaard, H Guo, R E Gur, R C Gur, T P Gurholt, R Hashimoto, S N Hatton, F A Henskens, D P Hibar, I B Hickie, L E Hong, J Horacek, F M Howells, H E Hulshoff Pol, C L Hyde, D Isaev, A Jablensky, P R Jansen, J Janssen, E G Jönsson, L A Jung, R S Kahn, Z Kikinis, K Liu, P Klauser, C Knöchel, M Kubicki, J Lagopoulos, C Langen, S Lawrie, R K Lenroot, K O Lim, C Lopez-Jaramillo, A Lyall, V Magnotta, R C W Mandl, D H Mathalon, R W McCarley, S McCarthy-Jones, C McDonald, S McEwen, A McIntosh, T Melicher, R I Mesholam-Gately, P T Michie, B Mowry, B A Mueller, D T Newell, P O'Donnell, V Oertel-Knöchel, L Oestreich, S A Paciga, C Pantelis, O Pasternak, G Pearlson, G R Pellicano, A Pereira, J Pineda Zapata, F Piras, S G Potkin, A Preda, P E Rasser, D R Roalf, R Roiz, A Roos, D Rotenberg, T D Satterthwaite, P Savadjiev, U Schall, R J Scott, M L Seal, L J Seidman, C Shannon Weickert, C D Whelan, M E Shenton, J S Kwon, G Spalletta, F Spaniel, E Sprooten, M Stäblein, D J Stein, S Sundram, Y Tan, S Tan, S Tang, H S Temmingh, L T Westlye, S Tønnesen, D Tordesillas-Gutierrez, N T Doan, J Vaidya, N E M van Haren, C D Vargas, D Vecchio, D Velakoulis, A Voineskos, J Q Voyvodic, Z Wang, P Wan, D Wei, T W Weickert, H Whalley, T White, T J Whitford, J D Wojcik, H Xiang, Z Xie, H Yamamori, F Yang, N Yao, G Zhang, J Zhao, T G M van Erp, J Turner, P M Thompson, G Donohoe

**Affiliations:** 1 Imaging Genetics Center, Stevens Neuroimaging & Informatics Institute, Keck School of Medicine, University of Southern California, Marina del Rey, CA, USA; 2 Harvard Medical School, Boston, MA, USA; 3 Melbourne Neuropsychiatry Centre, Department of Psychiatry, University of Melbourne and Melbourne Health, Carlton South, VIC, Australia; 4 Maryland Psychiatric Research Center, Department of Psychiatry, University of Maryland School of Medicine, Baltimore, MD, USA; 5 NORMENT, KG Jebsen Centre for Psychosis Research, Division of Mental Health and Addiction, Oslo University Hospital and Institute of Clinical Medicine, University of Oslo, Oslo, Norway; 6 Department of Clinical Neuroscience, Centre for Psychiatry Research, Karolinska Institutet, Stockholm, Sweden; 7 Department of Psychiatric Research, Diakonhjemmet Hospital, Oslo, Norway; 8 University of Edinburgh, Edinburgh, UK; 9 University of Oslo, Oslo, Norway; 10 Child and Adolescent Psychiatry Department, Hospital General Universitario Gregorio Marañón, School of Medicine, Universidad Complutense, IiSGM, CIBERSAM, Madrid, Spain; 11 Laboratory of Neuropsychiatry, Department of Clinical and Behavioral Neurology, IRCCS Santa Lucia Foundation, Rome, Italy; 12 Department of Psychiatry, Brigham and Women’s Hospital, Harvard Medical School, Boston, MA, USA; 13 Florey Institute of Neuroscience and Mental Health, Parkville, VIC, Australia; 14 Department of General Practice, The University of Melbourne, Parkville, VIC, Australia; 15 Swinburne University of Technology, Melbourne, VIC, Australia; 16 Department of Psychiatry, Brain Center Rudolf Magnus, University Medical Center Utrecht, Utrecht, The Netherlands; 17 Neuroscience Research Australia and School of Psychiatry, University of New South Wales, Sydney, NSW, Australia; 18 University of New Mexico, Albuquerque, NM, USA; 19 The Department of Electrical and Computer Engineering, University of New Mexico, Albuquerque, NM, USA; 20 The Mind Research Network, Albuquerque, NM, USA; 21 Centre for Neuroimaging and Cognitive Genomics (NICOG), Clinical Neuroimaging Laboratory, NCBES Galway Neuroscience Centre, National University of Ireland Galway, Galway, Ireland; 22 Discipline of Psychiatry, School of Medicine, University of Queensland, Herston, QLD, Australia; 23 Department of Computer Science and Engineering, The Ohio State University, Columbus, OH, USA; 24 Beijing Huilongguan Hospital, Beijing, China; 25 Worldwide Research and Development, Pfizer, Cambridge, MA, USA; 26 Santa Lucia Foundation, Rome, Italy; 27 Department of Psychiatry, Seoul National University College of Medicine, Seoul, Republic of Korea; 28 Department of Psychiatry and Neuropsychiatric Genetics Research Group, Institute of Molecular Medicine, Trinity College Dublin, Dublin, Ireland; 29 University Hospital Marqués de Valdecilla, IDIVAL, Department of Medicine and Psychiatry, School of Medicine, University of Cantabria, Santander, Spain; 30 CIBERSAM, Centro Investigación Biomédica en Red Salud Mental, Santander, Spain; 31 Department NESMOS, Faculty of Medicine and Psychology, University ‘Sapienza’ of Rome, Rome, Italy; 32 Department of Neurology and Psychiatry, Sapienza University of Rome, Rome, Italy; 33 Center for Addiction and Mental Health, Toronto, ON, Canada; 34 Division of Psychological and Social Medicine and Developmental Neurosciences, Technische Universität Dresden, Faculty of Medicine, University Hospital C.G. Carus, Dresden, Germany; 35 University of New South Wales, School of Psychiatry, Sydney, NSW, Australia; 36 The University of Queensland, Queensland Brain Institute and Centre for Advanced Imaging, Brisbane, QLD, Australia; 37 University of California, VAMC, San Francisco, CA, USA; 38 Department of Psychiatry and Mental Health, University of Cape Town, Cape Town, South Africa; 39 Division of Cerebral Integration, National Institute for Physiological Sciences, Aichi, Japan; 40 Olin Neuropsychiatric Research Center, Institute of Living, Hartford Hospital and Department of Psychiatry, Yale University School of Medicine, New Haven, CT, USA; 41 Departments of Psychiatry and Radiology, Massachusetts General Hospital, Harvard Medical School, Boston, MA, USA; 42 Department of Psychiatry and Human Behavior, University of California Irvine, Irvine, CA, USA; 43 Zhumadian Psychiatry Hospital, Henan Province, China; 44 Department of Psychiatry, University of Pennsylvania, Philadelphia, PA, USA; 45 Molecular Research Center for Children's Mental Development, United Graduate School of Child Development, Osaka University, Osaka, Japan; 46 Department of Psychiatry, Osaka University Graduate School of Medicine, Osaka, Japan; 47 Brain and Mind Centre, University of Sydney, Sydney, NSW, Australia; 48 School of Electrical Engineering and Computer Science, University of Newcastle, Callaghan, NSW, Australia; 49 Health Behaviour Research Group, University of Newcastle, Callaghan, NSW, Australia; 50 Hunter Medical Research Institute, Newcastle, NSW, Australia; 51 National Institute of Mental Health, Klecany, Czech Republic; 52 Third Faculty of Medicine, Charles University, Prague, Czech Republic; 53 University of Western Australia, Perth, WA, Australia; 54 Erasmus University Medical Center, Rotterdam, The Netherlands; 55 Laboratory for Neuroimaging, Department of Psychiatry, Psychosomatic Medicine and Psychotherapy, Goethe University, Frankfurt/Main, Germany; 56 Brain and Mental Health Laboratory, Monash Institute of Cognitive and Clinical Neurosciences, School of Psychological Sciences and Monash Biomedical Imaging, Monash University, Clayton, VIC, Australia; 57 Department of Psychiatry, Lausanne University Hospital (CHUV), University of Lausanne, Lausanne, Switzerland; 58 Departments of Psychiatry and Radiology, Brigham and Women's Hospital, Harvard Medical School, Boston, MA, USA; 59 Sunshine Coast Mind and Neuroscience Institute, University of the Sunshine Coast QLD, Australia, Brain and Mind Centre, University of Sydney, Sydney, NSW, Australia; 60 Department of Psychiatry, University of Minnesota, Minneapolis, MN, USA; 61 Research Group in Psychiatry (GIPSI), Department of Psychiatry, Faculty of Medicine, Universidad de Antioquia, Mood Disorder Program, Hospital Universitario San Vicente Fundación, Medellín, Colombia; 62 Department of Psychiatry, Massachusetts General Hospital, Harvard Medical School, Boston, MA, USA; 63 University of Iowa, Iowa City, IA, USA; 64 VABHS, Harvard Medical School, Boston, MA, USA; 65 Department of Psychiatry, Trinity College Dublin, Dublin, Ireland; 66 Department of Psychiatry and Biobehavioral Sciences, University of California, Los Angeles, Los Angeles, CA, USA; 67 The University of Texas Health Science Center at Houston, Houston, TX, USA; 68 Harvard Medical School and Massachusetts Mental Health Center Public Psychiatry Division of the Beth Israel Deaconess, Medical Center, Boston, MA, USA; 69 The University of Newcastle, Newcastle, NSW, Australia; 70 Schizophrenia Research Institute, Sydney, NSW, Australia; 71 Queensland Brain Institute, The University of Queensland, Brisbane, QLD, Australia and Queensland Centre for Mental Health Research, Brisbane and Queensland Centre for Mental Health Research, Brisbane, QLD, Australia; 72 Centre for Neural Engineering (CfNE), Department of Electrical and Electronic Engineering, University of Melbourne, Parkville, VIC, Australia; 73 The Florey Institute of Neuroscience and Mental Health, University of Melbourne, Melbourne, VIC, Australia; 74 Instituto de Alta Tecnología Médica, Medellín, Colombia; 75 School of Biomedical Sciences, Faculty of Health, the University of Newcastle, Callaghan, NSW, Australia; 76 Priority Centre for Brain and Mental Health Research, The University of Newcastle, Newcastle, NSW, Australia; 77 SU/UCT MRC Unit on Anxiety and Stress Disorders, Department of Psychiatry, Stellenbosch University, Stellenbosch, South Africa; 78 Murdoch Childrens Research Institute, The Royal Children’s Hospital, Parkville, VIC, Australia; 79 Neuroscience Research Australia, Sydney, NSW, Australia; 80 School of Psychiatry, University of New South Wales, Sydney, NSW, Australia; 81 VA Boston Healthcare System, Boston, MA, USA; 82 Division of Neuropsychiatry, Menninger Department of Psychiatry and Behavioral Sciences, Baylor College of Medicine, Houston, TX, USA; 83 Department of Psychiatry and MRC Unit on Anxiety and Stress Disorders, University of Cape Town, Cape Town, South Africa; 84 Department of Psychiatry, School of Clinical Sciences, Monash University and Monash Health, Clayton, VIC, Australia; 85 Chongqing Three Gorges Central Hospital, Chongqing, China; 86 Department of Psychology, University of Oslo, Oslo, Norway; 87 Neuroimaging Unit, Technological Facilities, Valdecilla Biomedical Research Institute IDIVAL, Santander, Spain; 88 Department of Psychiatry, University of Iowa, Iowa City, IA, USA; 89 Research Group in Psychiatry (GIPSI), Department of Psychiatry, Faculty of Medicine, Universidad de Antioquia, Medellín, Colombia; 90 Neuropsychiatry Unit, Royal Melbourne Hospital, Parkville, VIC, Australia; 91 Kimel Family Translational Imaging-Genetics Research Laboratory, Campbell Family Mental Health Research Institute, CAMH Department of Psychiatry, University of Toronto, Toronto, ON, Canada; 92 Luoyang Fifth People's Hospital, Henan Province, China; 93 Department of Psychiatry, Yale University School of Medicine, New Haven, CT, USA; 94 Department of Computer Science and Electrical Engineering, University of Maryland, Baltimore, MD, USA; 95 School of Psychology, Shaanxi Normal University and Key Laboratory for Behavior and Cognitive Neuroscience of Shaanxi Province, Xi’an, Shaanxi, China; 96 Psychology Department & Neuroscience Institute, Georgia State University, Atlanta, GA, USA

## Abstract

The regional distribution of white matter (WM) abnormalities in schizophrenia remains poorly understood, and reported disease effects on the brain vary widely between studies. In an effort to identify commonalities across studies, we perform what we believe is the first ever large-scale coordinated study of WM microstructural differences in schizophrenia. Our analysis consisted of 2359 healthy controls and 1963 schizophrenia patients from 29 independent international studies; we harmonized the processing and statistical analyses of diffusion tensor imaging (DTI) data across sites and meta-analyzed effects across studies. Significant reductions in fractional anisotropy (FA) in schizophrenia patients were widespread, and detected in 20 of 25 regions of interest within a WM skeleton representing all major WM fasciculi. Effect sizes varied by region, peaking at (*d*=0.42) for the entire WM skeleton, driven more by peripheral areas as opposed to the core WM where regions of interest were defined. The anterior corona radiata (*d*=0.40) and corpus callosum (*d*=0.39), specifically its body (*d*=0.39) and genu (*d*=0.37), showed greatest effects. Significant decreases, to lesser degrees, were observed in almost all regions analyzed. Larger effect sizes were observed for FA than diffusivity measures; significantly higher mean and radial diffusivity was observed for schizophrenia patients compared with controls. No significant effects of age at onset of schizophrenia or medication dosage were detected. As the largest coordinated analysis of WM differences in a psychiatric disorder to date, the present study provides a robust profile of widespread WM abnormalities in schizophrenia patients worldwide. Interactive three-dimensional visualization of the results is available at www.enigma-viewer.org.

## Introduction

Schizophrenia, a debilitating psychiatric disorder with a considerable societal burden, has been a major focus of neuroimaging studies for decades, yet its neurobiology remains only partially understood.^1^ Cumulative evidence has led to a dysconnectivity hypothesis—that schizophrenia may involve abnormal or inefficient communication between functional brain regions,^2^ and disturbances in the underlying pattern of white matter (WM) structural organization.^3, 4, 5^ Genetic and histopathological studies reveal oligodendrocyte and myelin abnormalities in schizophrenia.^4, 6, 7^ Furthermore, WM genetic variants have been found to overlap with genes associated with liability for schizophrenia.^8^ However, consistent alterations in WM microstructure have been difficult to identify. Diffusion tensor imaging (DTI) allows for the *in vivo* study of WM microstructural properties that cannot be made with standard magnetic resonance imaging alone. DTI has revealed WM changes beyond volumetric differences in unmedicated, first-episode and chronically affected schizophrenia patients,^9, 10^ but the anatomical scope and strength of these effects varies widely across studies.^11^

WM disruptions in DTI studies of schizophrenia patients are commonly identified, with significantly lower fractional anisotropy (FA) in patients compared with controls. Generally, findings have implicated prefrontal and temporal lobes,^5, 12, 13^ and the fiber tracts connecting these regions.^14^ Abnormalities have been linked to cognitive deficits and symptoms in schizophrenia, including memory impairment and auditory hallucinations.^13, 15^ However, the considerable heterogeneity in both the effect sizes and the regional distribution of FA reduction reported across studies have limited the conclusions drawn to date. In a review by Kanaan *et al.*,^3^ all studies included reported altered FA in schizophrenia patients, but the specific regions implicated were highly equivocal: each region implicated had a roughly equal chance of being identified versus not in a given study. It also remains unclear whether WM abnormalities are localized to specific tracts or distributed throughout the brain, and whether specific networks are differentially disrupted.^11, 16^

Sources of heterogeneity between studies may be attributed to imaging protocols, scanner differences, differences in patients’ ages at time of scanning, age at illness onset, duration of illness, symptom severity, medications and other variations in demographic and socioeconomic factors. Study samples also vary in size that alone may have a significant effect on the reported findings.^17^ These factors may modulate the degree of WM abnormalities detectable with DTI.^10^

To address the variations in methods, and boost statistical power, the Schizophrenia Working Group of the Enhancing Neuroimaging Genetics through Meta-Analysis consortium (ENIGMA-Schizophrenia) initiated the first worldwide initiative to pool effect sizes from a harmonized, coordinated DTI analysis. Our primary goal was to identify WM microstructural measures derived from DTI with the most robust disease effects. Focusing on FA, and further exploring DTI-derived diffusivity measures of both whole-brain and atlas-defined WM regions of interest (ROIs), we coordinated a large-scale meta-analysis using the established ENIGMA-DTI protocol to harmonize the processing of diffusion data from multiple sites (http://enigma.usc.edu/ongoing/dti-working-group/).^18, 19, 20^ These analytic techniques enable a coordinated meta-analysis of independent samples worldwide with an effort to remove biases in pooling, in contrast to retrospective meta-analyses based on published findings.

We set out to identify consistent and robust WM differences between individuals with schizophrenia and healthy controls in an unprecedented sample of 4322 individuals scanned across 29 cohorts from Australia, Asia, Europe, South Africa and North America. We hypothesized moderate effect sizes across the brain, based on previous volumetric findings;^21^ yet we aimed to order regions according to consistency and magnitude of effect size, thereby regionally identifying WM that is most severely disrupted in schizophrenia. We aimed to also determine whether disease-related factors (including duration of illness, age at onset of schizophrenia, antipsychotic medication, smoking and severity of positive and negative symptoms) are also associated with differences in WM microstructure.

## Materials and methods

### Study samples

The ENIGMA-Schizophrenia DTI working group currently comprises 29 cohorts from 14 countries totaling 2359 healthy controls and 1963 individuals with schizophrenia (see Supplementary Figure 1).

The mean age across samples was 36.14 years for controls (range: 18–86) and 36.22 years for patients (range: 18–77). Samples of controls and patients were ∼53.4% and 67% males respectively, but effects of sex were also modeled. The mean age at onset and duration of illness across the patient groups were 23 and 14 years, respectively. The mean total Positive and Negative Syndrome Scale (PANSS) and Scale for the Assessment of Positive and Negative Symptoms (SANS and SAPS) across the samples were 55.90, 16.72 and 13.70, respectively. For samples that recorded current antipsychotic type and dose, the fraction of patients on second-generation antipsychotics (atypical) was 71%, first generation (typical) was 6%, both 10% and neither 13%. As in van Erp *et al.*,^21^ chlorpromazine (CPZ) equivalents were computed using methods previously described in Woods (2005; http://www.scottwilliamwoods.com/files/Equivtext.doc). The mean CPZ dose equivalent across the samples was 371.23. Supplementary Tables 2 and 3 summarize key clinical and demographic information. Each study sample had been assessed with participants’ written informed consent approved by local institutional review boards. Individuals with bad-quality diffusion images were excluded from the analysis.

### Imaging acquisition and processing

Details of study type, scanner and acquisition parameters for each of the 29 sites are provided in Supplementary Table 1. Preprocessing, including eddy current correction, echo-planar imaging-induced distortion correction and tensor fitting, was carried out at each site. Recommended protocols and procedures as well as quality control pipelines are available as part of the ENIGMA-DTI webpage and NITRC (Neuroimaging Informatics Tools and Resources Clearinghouse). Harmonization of preprocessing schemes was not enforced across sites to allow individual sites to use existing pipelines that may be more appropriate for their data acquisition. Once tensors were estimated, harmonized image analysis of DTI measure of FA was conducted at each site using the ENIGMA-DTI protocol (see Supplementary Note 1). As part of a secondary analysis, mean diffusivity (MD), axial diffusivity (AD) and radial diffusivity (RD) images were also derived. The present analysis combined ROIs across both hemispheres to avoid any potential issues of left/right flipping. Lateralized results are reported in Supplementary Table 4 and Supplementary Note 5 but should be interpreted with caution.

### Statistical analysis

#### Per-site analysis

We evaluated FA, and available MD, AD and RD differences between schizophrenia cases and healthy controls by calculating Cohen’s *d* effect size estimates for diagnosis of schizophrenia in each of the 25 ROIs listed in Table 1.

To tease apart regional WM effects from global differences, *post hoc* analyses were carried out to covary for the effects of global FA measures across the entire skeleton, including average FA across the full skeleton, the central, or ‘core’ FA comprising the average of all voxels in the JHU (Johns Hopkins University) ROIs, and the peripheral regions defined as everything besides the core (non-JHU) in the full skeleton analyzed (see Supplementary Note 2 for calculations of core and periphery FA).

Cohen’s *d* effect sizes were also calculated for differences in FA between patients on atypical antipsychotics, typical antipsychotics, both and unmedicated. Differences in FA were assessed between subgroups of patients who were smokers versus nonsmokers. Multiple linear regressions were performed to examine the effects of age at onset, duration of illness, CPZ scores, PANSS total, positive and negative scores and SAPS and SANS total scores. Age, sex, age-by-sex interaction and quadratic covariates of age^2^ and age^2-^by-sex interaction were modeled as linear and nonlinear age and sex interactions have been reported for FA.^22^ As age and duration of illness are collinear, we also examined duration of illness in years without covarying for age, as well as duration expressed as the percent of a person’s lifetime they had been ill. Interaction effects, including diagnosis by sex and diagnosis by age, were also calculated. A minimum of 10 subjects per group was used as the cutoff for inclusion in the statistical analyses. An independent *t*-test between s.d. measures for average FA between patients (s.d.=0.0176) and controls (s.d.=0.0194) revealed no significant difference in variance between the groups (*P*>0.0.5).

### Code availability

All analyses were conducted using generalizable scripts available on the ENIGMA-GitHub https://github.com/ENIGMA-git/ENIGMA/tree/master/WorkingGroups/EffectSize_and_GLM. Individual sites downloaded a single set of R scripts and specified the set of regressions that were customized for the current ENIGMA-Schizophrenia DTI analysis, publicly available on a set of Google Spreadsheet configuration files. Standardized regression outputs were then uploaded to a central server for meta-analysis.

### Meta-analysis

As in prior ENIGMA disease working group meta-analyses,^21, 23, 24, 25^ a random-effects inverse-variance weighted meta-analysis was conducted at a central coordinating site (the University of Southern California Imaging Genetics Center) in R (metafor package, version 1.99–118 http://www.metafor-project.org/) to combine individual site effect sizes (See Supplementary Note 3). Heterogeneity scores (*I*^2^) for each test were also computed, indicating the percent of the total variance in effect size explained by heterogeneity of the effects alone. Lower values of *I*^2^ indicate lower variance in the effect size estimates across studies.

Effect sizes are reported as overall Cohen’s *d* values for case/control effects and *Z*-scores for quantitative effects from linear regressions. To control the reporting of false positives from multiple tests across the ROIs, for our primary analysis of FA differences in cases as compared with controls, effects were declared to be significant if they survived the Bonferroni correction threshold of 0.05/25=0.002.

## Results

### FA differences between schizophrenia patients and controls

**Figure 1:**
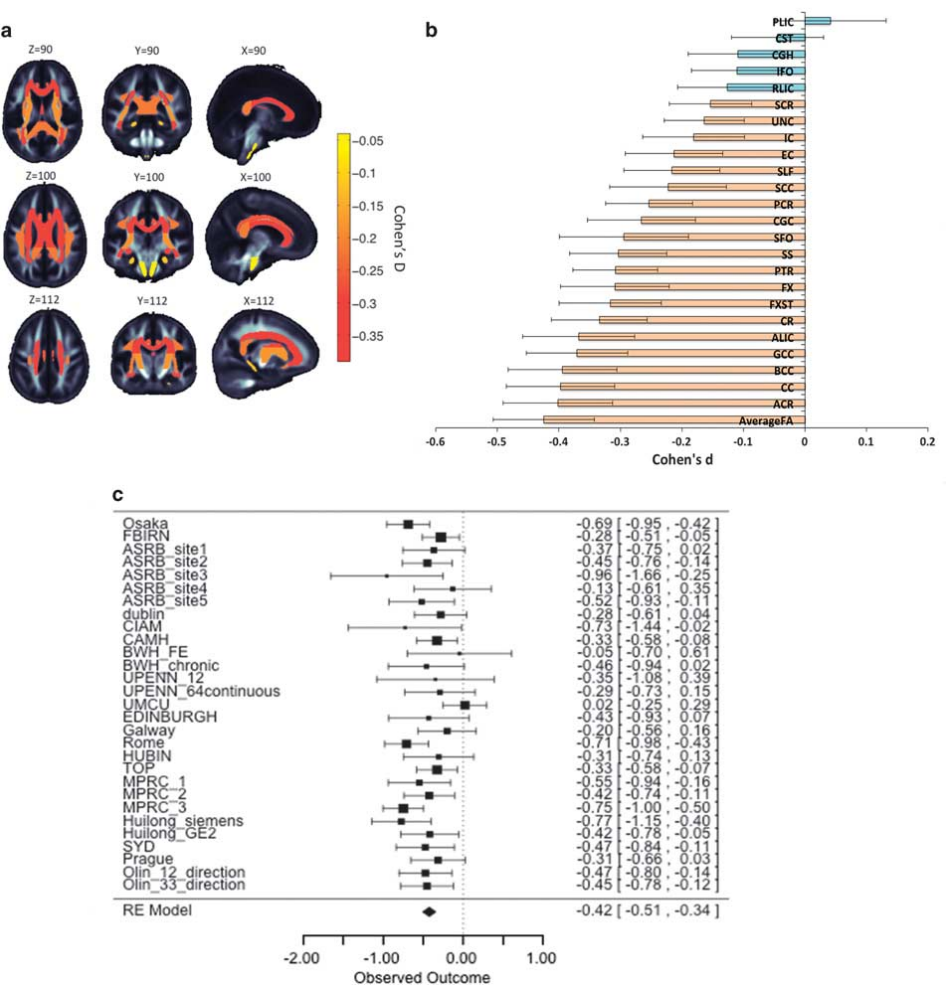
(**a**) Fractional anisotropy (FA) differences between schizophrenia patients and healthy controls for 25 white matter (WM) regions representing major fasciculi. Gradient bar indicates Cohen’s *d* effect sizes after meta-analysis. (**b**) Cohen’s *d* effect sizes after meta-analysis, sorted in increasing magnitude of Cohen’s *d* effect sizes across 29 cohorts for FA differences in schizophrenia patients (*N*=1963) versus healthy controls (*N*=2359), after including age, sex, age × sex, age^2^ and age^2^ × sex as covariates. Error bars represent 95% confidence intervals. Significant regions after adjusting for multiple regions tested (*P*<0.05/25=0.002) are highlighted in orange. (**c**) Forest plot of effect sizes for 29 cohorts. Interactive three-dimensional (3D) visualization of the results is available at www.enigma-viewer.org.

Of the 25 regions, 20 showed significantly lower FA in patients. Based on previous meta-analysis^5^ we hypothesized that WM tracts interconnecting the frontal lobe, thalamus, cingulate gyrus and regions of the temporal lobe would be most severely affected. The largest effect size was observed for lower *average* FA (across the whole-brain WM skeleton) in schizophrenia patients, followed by the anterior corona radiata (ACR), the whole corpus callosum (CC), body of the CC and the genu of the CC (GCC). Significant patient reductions were also found in a further 15 ROIs (see Figure 1, Table 1 and Supplementary Figure 5).

### MD and RD differences between schizophrenia patients and healthy controls

**Figure 2:**
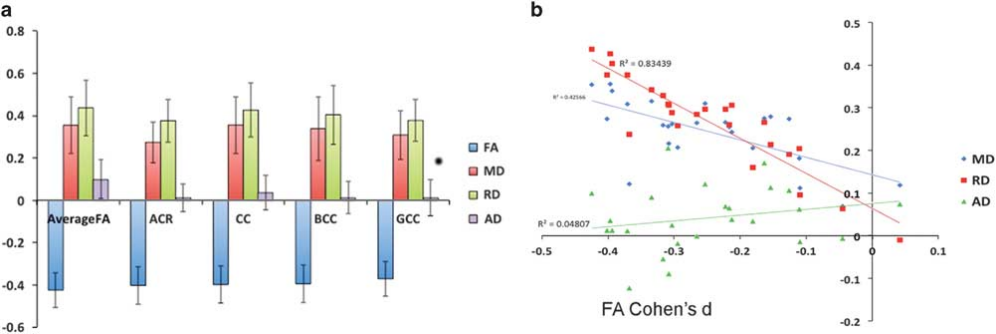
(**a**) Cohen’s *d* effect sizes, after meta-analysis, for fractional anisotropy (FA), mean diffusivity (MD) and radial diffusivity (RD) differences in schizophrenia patients versus healthy controls, after including age, sex, age × sex, age^2^ and age^2^ × sex as covariates for the top four regions of interest (ROIs) showing the largest FA effects (average FA, body of corpus callosum (BCC), corpus callosum (CC), anterior corona radiata (ACR) and genu of corpus callosum (GCC)). Error bars represent the 95% confidence intervals. The corpus callosum, ACR and FA across the whole brain are among the measures that show most robust effects in cohorts worldwide. (**b**) Relationship between FA and diffusivity (mean, axial and radial) Cohen’s *d* effect sizes after meta-analysis for differences between schizophrenia patients and healthy controls. The effect of RD is highly correlated, whereas axial diffusivity (AD) shows no correlation and low effect sizes.

A secondary analysis was carried out using established measures of WM diffusivity (MD, AD and RD) for all 29 cohorts. Patients had significantly higher MD across 17 ROIs and significantly higher RD across 21 ROIs. Effects observed were larger and more widespread for RD. Finally, patients had significantly higher AD in the fornix in comparison with healthy controls (Figure 2, Supplementary Figures 6).

### Sex differences

To examine differences between males and females in relation to diagnosis, sex-by-diagnosis interactions were conducted. Although we did not detect a significant interaction effect, we observed significant differences in effect size when analyzing males and females separately, with females showing significantly larger effects for decreased FA (t=−10.83, *P*=0.001) in comparison with males, as seen through a paired *t*-test. See Supplementary Figures 10 and 11 and Supplementary Note 6.

### Controlling for average, core and peripheral FA

To tease apart regional WM effects from the global differences, a *post hoc* analysis was conducted in individual tract ROIs (as previously performed) but additionally covarying for (1) average FA across the entire skeleton (the average FA contains *both* core and periphery voxels), (2) average FA within the core of the skeleton only, within which ROIs are defined as part of the JHU atlas or (3) the average FA within the periphery of the skeleton only—areas on the skeleton not included in the ROIs of the JHU atlas (non-JHU).

Covarying for *average FA*, there is significantly increased FA in the posterior limb of internal capsule (PLIC) only (*P*<0.002). After covarying for *core FA*, there is significantly decreased average FA, body of corpus callosum, fornix, CC and anterior limb of internal capsule (ALIC), as well as increased FA in the PLIC (*P*<0.002). Finally, after covarying for *periphery FA*, no effects remain significant after multiple comparison correction (*P*<0.05), suggesting the effects are being driven by the peripheral WM regions and no individual ROI in the core of the WM goes significantly above and beyond those effects. See Supplementary Figures 2.

### Association of clinical traits with FA

#### Age by diagnosis

To examine differences in the effects of age on FA between patients and controls, we performed age-by-diagnosis interaction tests for each ROI. A significant age-by-diagnosis interaction was observed in the ACR (β=−0.00037, *P*=0.0001) and across the average FA skeleton (β=−0.00018, *P*=0.0003), with patients showing accelerated FA reductions with age (see Supplementary Figure 12).

### Duration of illness and age at onset

To investigate whether WM microstructure may change with illness duration, reflecting a progressive disease process, we conducted correlations between duration of illness and FA for patients across all ROIs. We found a significant inverse association between FA and duration of illness in years for 10 of the 25 ROIs (see Table 2), without age included in the regression model (*N*=1178, 20 sites). When covarying for age, the relationship between duration of illness and FA is nonsignificant. No significant association between FA and duration of illness expressed as a percentage was observed. Similarly, no significant associations between FA and age at onset of schizophrenia were observed (*N*=1161, 20 cohorts).

### Symptom severity

To investigate whether WM changes were associated with positive and negative symptom severity in schizophrenia, we conducted correlations between symptom severity scores—as measured by PANSS positive, PANSS negative, SAPS and SANS—and FA across all ROIs. Trending negative correlations were observed between SANS total scores (*N*=433, 9 cohorts) and FA of the ALIC, CC, cingulum, IC and posterior thalamic radiation, as well as between SAPS total scores (*N*=406, 9 cohorts) and FA of the CC and GCG, but the effects did not survive correction for multiple comparisons. Trending negative associations were also observed between PANSS negative (*N*=674, 10 cohorts) scores in the ALIC, IFO, posterior thalamic radiation and SCC and PANSS total scores (*N*=695, 10 cohorts) in the ALIC, CC, SCC and SCR. Similarly, these effects did not survive correction for multiple comparisons (see Supplementary Tables 5 and 6).

### Medication and smoking

To assess the potential impact of antipsychotic medication on WM changes in schizophrenia, we carried out correlations between CPZ equivalent scores (Woods, 2005, http://www.scottwilliamwoods.com/files/Equivtext.doc) and FA. No significant associations between FA and CPZ scores were detected (*N*=627, 10 cohorts). The current study was underpowered to compare patients based on their atypical or typical antipsychotic use in a multisite framework and therefore we decided to omit this from the analysis. Using a cutoff of *N*=10 per group, only 31 patients from two cohorts were taking both typical and atypical medication, 27 patients from 2 cohorts were taking typical antipsychotics only and 68 patients from 4 cohorts were unmedicated. The majority of patients (*N*=486 across 10 cohorts) were taking atypical antipsychotics. Finally, to investigate the effects of nicotine intake on WM in schizophrenia, we examined differences between smokers and nonsmokers in the patient group. No significant differences in FA between smokers and nonsmokers in the patient group were observed. There were 248 nonsmoker patients and 260 smoker patients across 6 cohorts.

## Discussion

In the largest coordinated meta-analysis to date—evaluating DTI data from 1963 individuals with schizophrenia and 2359 healthy participants—we found a highly consistent pattern of affected WM in cohorts recruited around the world. FA was lower for patients globally across the whole-brain WM skeleton and significantly detected in 19 out of 24 tract-based ROIs, including the CC (specifically body and genu), the CR (anterior, posterior and superior), ALIC, fornix, posterior thalamic radiation, superior fronto-occipital fasciculus, sagittal stratum, cingulum, superior longitudinal fasciculus, external capsule, IC and uncinate (see Table 1). Largest effects were observed for whole brain average FA, ACR, CC, body of corpus callosum, genu of the CC and ALIC, all showing Cohen’s *d* magnitudes of >0.35 (medium effect sizes, according to Cohen’s criteria^26^). When controlling for global effects, none of the individual tract regions showed significant case/control associations beyond those detected globally, with the exception of increased FA in the PLIC for patients. Widespread increases in mean and radial diffusivity for patients were also observed, as well as a subtle increase in axial diffusivity of the fornix. This ranking of regions may guide and inform future studies of schizophrenia with DTI. The current sample size is also well powered to detect Cohen’s *d* values as small as 0.10 (see Supplementary Note 4 and Supplementary Table 7). Similar magnitudes of effect were also observed in a prior coordinated meta-analysis of subcortical volumes in schizophrenia^21^ where effects ranged from −0.46 to −0.37. The uniform direction and widespread distribution of these differences may support the concept of schizophrenia as a disorder of global structural dysconnectivity. Schizophrenia as a disorder that affects WM globally is supported by recent studies of the human connectome indicating that although long-range WM connections between brain hubs are most severely disrupted in the disorder, connectivity deficits are widespread and span virtually the entire connectome.^16, 27^ The assumption of global (instead of localized) FA deficits in schizophrenia has also been supported by alternative analytical approaches based on systematic resampling of an original data set.^17^

In the context of a structural dysconnectivity hypothesis in schizophrenia, we were able to provide a more accurate estimate of WM alterations in the disorder. We analyzed data from 29 cohorts with a total sample of 4322 individuals using a common analysis pipeline we had previously developed.^18, 19, 20^ This allowed for a coordinated prospective meta-analysis of the data, unlike traditional meta-analyses that attempt to combine statistical results from the literature. This approach addresses, for we believe the first time, issues of low power and inconsistencies of analysis that have contributed to the heterogeneity and ambiguity in results of DTI studies in schizophrenia to date.

### Regional specificity of WM findings in schizophrenia

The most consistent findings in the schizophrenia literature until now have included aberrant interhemispheric and fronto-temporal connectivity.^5, 12, 13^ Magnetic resonance imaging studies show smaller volume of the CC and its subregions in chronic, first-episode and high-risk individuals, suggesting that it is a stable feature of the disorder.^28, 29, 30^ Reduced FA of the CC was also reported in a prior retrospective meta-analysis of 15 DTI studies in schizophrenia^5^ and a meta-analysis of studies examining both gray and WM.^31^ These findings suggest that abnormal interhemispheric communication may play a role in the etiology of schizophrenia.

WM FA differences in the ALIC and ACR are consistently reported in schizophrenia.^32, 33^ The ALIC connects the thalamus and prefrontal cortex and FA of this region has been shown to be correlated with verbal and nonverbal episodic memory in single-site studies of schizophrenia.^32, 33^ In line with prior DTI studies, we report significantly decreased FA for the ALIC and not the PLIC.^33^ The corona radiata contains reciprocal connections from the thalamus to the cerebral cortex and is thought to be involved in information processing. Reductions in FA of this pathway have been associated with increased severity of auditory verbal hallucinations in schizophrenia.^34^ A medium effect was also observed for the fornix, a structure implicated in cognitive disturbances and memory function in schizophrenia.^35^ A subtle increase in axial diffusivity for patients was also observed in this region. The fornix is particularly vulnerable to partial volume effects, and hence WM measures in this region are inconsistent.^36, 37^ Therefore, the current findings in the fornix should be interpreted with caution. Reliability estimates for all ROIs are reported in the Supplementary Tables 8.

Although the largest effects were observed for WM tracts of frontotemporal, interhemispheric and corticothalamic regions, the current findings suggest a global mechanism for WM dysconnectivity in schizophrenia. After controlling for average and periphery FA, non-significant differences are observed for almost all ROIs, with the exception of increased FA in the PLIC for patients, suggesting that the effect for average FA of the WM skeleton is driving the difference in FA across almost all of the ROIs. Patterns of widespread FA decreases and localized FA increases have been previously found in schizophrenia, with the latter thought to be a result of aberrant axonal pruning.^16, 38^

### Demographic and clinical correlates of WM changes in schizophrenia

No significant sex-by-diagnosis interactions were observed in the current analysis, but females had significantly larger effect sizes for lower FA overall. Post-mortem studies suggest that WM abnormalities are more pronounced in female patients^39, 40^ and an *in vivo* DTI analysis^41^ reported decreased FA in the CC for female patients compared with males. The etiology of these sex differences is unclear, but the findings suggest subtle sex-dependent alterations of WM in schizophrenia.

Duration of illness was negatively correlated with FA in 10 of the 25 WM ROIs. As duration of illness is highly correlated with age, these effects are hard to separate. After covarying for age, the relationship between FA and duration of illness is no longer significant. Longitudinal analyses of first-episode, high-risk and chronic patients are needed to distinguish effects of age and duration of illness. Significant age-by-diagnosis interactions were found in the ACR and across the average FA. Unlike previous findings,^42^ Kochunov *et al.*^43^ report similar age-by-diagnosis interactions in these regions, indicating that schizophrenia patients are more susceptible to faster age-related decline in FA values of WM regions that mature later in life.

Trend-wise negative relationships between positive and negative symptom severity scores and WM FA of regions of the CC, IC and thalamic radiations were also observed. WM integrity of these regions has previously been associated with severity of positive, negative and cognitive symptoms.^33, 44, 45, 46^ Parsing these measures based on the individual items of the positive and negative symptom scales may reveal more robust associations with WM FA. No significant effects of age at onset of schizophrenia or medication dosage were detected in the current study. Many cross-sectional DTI studies report no detectable correlations between medication and anisotropy.^10, 47^

### Biological basis of WM abnormalities

This study confirms a brain-wide pattern of reduced FA and focal increased FA in schizophrenia, with the latter hypothesized to occur as a consequence of aberrant axonal pruning.^16, 38^ By evaluating WM on a common skeleton, we are able to focus on microstructural differences independent of gray matter volumetric abnormalities. In comparing our findings with the ENIGMA-Schizophrenia study of subcortical volumes,^21^ we note that the maximal effect sizes are similar (Cohen’s *d* ∼−0.4), yet in that work, effects were found to be specific to certain subcortical structures including the hippocampus, amygdala and thalamus, whereas structures of the basal ganglia, including the caudate, putamen and pallidum, showed no effect. This implies more global deficiencies in WM whereas volume differences are more localized, perhaps in part because of the effect of added comorbidities. In a proof-of-concept work, Franke *et al.*^48^ were unable to find genetic overlap between schizophrenia and subcortical volumes. It is yet to be determined whether WM, which is more globally affected, may serve as a more promising endophenotype. Altered WM connectivity is postulated to play a significant role in the etiology of schizophrenia, but the mechanisms leading to altered connectivity remain unknown. Currently, hypotheses about the causative basis of this dysconnectivity are varied and include changes in oligodendrocyte and microglial function.^49^ As noted above, age at onset, duration of illness (covarying for age) and medication dosage did not explain a significant proportion of WM variation, suggesting that whatever the causes, their effects do not overlap exclusively with the period of clinical diagnosis and treatment. However, WM deterioration may also be a consequence of the disorder. Cropley *et al.*^50^ found that gray matter volume loss predated WM loss, with accelerated WM deterioration with age in schizophrenia, detecting significant WM differences in patients only detected after age 35. Furthermore, Pasternak *et al.*^51, 52^ found increased free water—a potential surrogate marker for neuroinflammation—in first-episode schizophrenia, with deterioration in free-water corrected FA in chronic schizophrenia. These results may indicate that WM abnormalities may be more pronounced and more widespread in later stages of the illness and may be predated by gray matter volume loss and inflammation in earlier stages of the illness. Future analyses of these data sets will examine available samples of high-risk, first-episode, early and chronic schizophrenia to assess whether these WM effects are present before illness onset and whether they progress throughout the illness.

### Study limitations

Despite being the largest of its kind, our study should be interpreted cautiously. This meta-analysis is cross-sectional and effects of age, age by diagnosis, along with durations of illness and/or medication exposure on WM microstructure may be more thoroughly investigated with a longitudinal design looking at rates of changes in the microstructural properties. The ENIGMA-DTI protocol has been validated for longitudinal analyses,^53^ but follow-up assessments of patients with diffusion imaging are limited within the ENIGMA-Schizophrenia Working group cohorts.

This current study is the first to show that effect sizes from harmonized DTI analyses may be pooled to detect reliable microstructural differences in patients from around the world. Future analyses aimed at harmonizing additional covariates across the ENIGMA groups may help tease apart effects. For example, accounting for genetic and environmental factors associated with schizophrenia, including general health conditions, lifestyle, additional recreational drugs or medications, trauma and stress, may add to a deepened understanding of the WM differences seen in this study.

In general, larger effects were observed in the larger ROIs containing a greater number of voxels, including the CC, and we found a medium but nonsignificant correlation between effect size and the square root of the number of voxels (sqrt(N)) in each ROI (see Supplementary Note 7). This is an issue that should be taken into consideration when interpreting regions where significant effects were not observed. As well as ROI size, we also investigated whether the number of gradient directions significantly affected Cohen’s *d* measures by splitting the cohorts into high direction (>30 directions) and low direction (<30 directions) groups. We found no significant differences in Cohen’s *d* measures between these groups (Supplementary Figure 9).

Lower FA in coherent fiber bundles may reflect abnormal fiber coherence or packing, or aberrations of axonal integrity and/or myelination. Although more detailed histopathological validations are needed, analyses of other diffusivity measures may help interpret the underlying microstructural abnormalities. Here we found widespread increased MD and RD in patients compared with controls, as well as increased AD in the fornix. This suggests that the observation of lower FA may be driven mainly by aberrant myelination across most ROIs, with axonal density reduction in the fornix, although these imaging-based estimates do not fully capture the underlying microscopic cellular processes to confirm this.^49^

Finally, tract-based spatial statistics (TBSS) is a widely used method for voxel-based analysis of WM that addresses issues associated with smoothing and misalignment in DTI group analysis.^54^ However, the method has some limitations. TBSS reduces WM tracts into a skeleton, delineating the center of the tracts and projecting onto it only the highest value FA along the projection that results in loss of information^55^ and potential artifacts,^56^ often a result of misregistration. For example, as with any population study that spatially normalizes brain images, misalignment can occur; for TBSS however, this can be a particular problem in the separation of adjacent WM tracts.^57^ Smaller tracts, such as the fornix, are more susceptible to misalignment. Approaches for retrospective multisite analysis of DTI are extremely limited, yet we, as ENIGMA-DTI, have conducted several test–retest and reliability analyses to ensure reproducibility of measures and effects using this TBSS approach.^53^ Although the regional analysis conducted here using TBSS is not able to capture all information regarding brain microstructure, the results from this work confirm the importance of WM alterations and global dysconnectivity. This will enable more in-depth studies, including voxelwise rather than ROI-based analyses and motivate the harmonization of tractography-based approaches. We hope this work shows the importance of pooling diffusion magnetic resonance imaging for large-scale studies and motivates additional methodological approaches for such multisite work, including harmonizing connectomic analyses and further evaluating tract-based measures, such as tract density and cortical connections.

## Conclusions

This study shows robust and widespread WM changes across multiple regions of interest for schizophrenia patients. Global microstructural alterations, corresponding to previously reported gray matter disruptions,^31^ provide further evidence that schizophrenia may be, in part, a disorder of global brain structural connectivity. The present study is the largest analysis of WM differences in schizophrenia to date, and the first to use harmonized protocols, allowing for more accurate estimates of effect size. The reported effect sizes also provide important information for power estimates in schizophrenia imaging studies. Our consortium is currently applying these methods across multiple samples of other psychiatric disorders, including major depression, bipolar disorder and obsessive compulsive disorder, that will eventually allow for a large-scale cross-disorder comparison of disease effects on WM (for an overview of ongoing projects in ENIGMA, see http://enigma.usc.edu). Comparing WM effects will no doubt shed further light on similarities and differences in the pathophysiology of these disorders.

## Figures and Tables

**Table 1:** Cohen’s *d* values, their s.e., *P*-values and *I* ^2^ (heterogeneity) values after meta-analysis for FA differences between schizophrenia patients and healthy controls

*ROI*	*Cohen’s* d	*s.e.*	P*-value*	I^*2*^	N *voxels*
Average FA	−0.42	0.042	4.5 × 10^−24^	34.65	112 889
ACR	−0.40	0.045	9.19 × 10^−19^	43.83	3129
CC	−0.40	0.045	8.36 × 10^−19^	42.51	7318
BCC	−0.39	0.045	2.51 × 10^−18^	43.24	3173
GCC	−0.37	0.042	1.24 × 10^−18^	35.22	1834
ALIC	−0.37	0.046	2.27 × 10^−15^	46.30	1510
CR	−0.33	0.040	3.16 × 10^−17^	27.89	7344
FXST	−0.32	0.042	8.28 × 10^−14^	36.39	706
FX	−0.31	0.045	6.76 × 10^−12^	43.25	222
PTR	−0.31	0.035	1.99 × 10^−18^	11.78	1987
SS	−0.30	0.040	4.91 × 10^−14^	30.34	1294
SFO	−0.29	0.054	3.96 × 10^−8^	59.68	193
CGC	−0.27	0.045	2.95 × 10^−9^	42.87	594
PCR	−0.25	0.036	2.39 × 10^−12^	15.76	1437
SCC	−0.22	0.048	4.39 × 10^−6^	50.94	2311
SLF	−0.22	0.040	5.61 × 10^−8^	29.17	3503
EC	−0.21	0.040	1.41 × 10^−7^	31.01	2896
IC	−0.18	0.042	1.80 × 10^−5^	36.25	4781
UNC	−0.16	0.033	8.78 × 10^−6^	4.18	125
SCR	−0.15	0.034	6.91 × 10^−6^	7.84	2778
RLIC	−0.13	0.041	0.0021	33.10	1496
IFO	−0.11	0.038	0.0035	22.70	88
CGH	−0.11	0.041	0.0082	33.73	524
CST	−0.04	0.038	0.24	24.20	167
PLIC	0.04	0.046	0.37	46.20	1775

Abbreviations: ACR, anterior corona radiata; ALIC, anterior limb of internal capsule; BCC, body of corpus callosum; CC, corpus callosum; CGC, cingulum; CGH, cingulum (hippocampal portion); CR, corona radiata; CST, corticospinal tract; EC, external capsule; FA, fractional anisotropy; FX, fornix; FXST, fornix stria terminalis; GCC, genu of corpus callosum; IC, internal capsule; IFO, inferior fronto occipital fasciculus; PCR, posterior corona radiata; PLIC, posterior limb of internal capsule; PTR, posterior thalamic radiation; RLIC, retrolenticular part of IC; ROI, region of interest; SCC, splenium of corpus callosum; SCR, superior corona radiata; SFO, superior fronto-occipital fasciculus; SLF, superior longitudinal fasciculus; SS, sagittal stratum; UNC, uncinate.

**Table 2:** Regression β-values, s.e. and *P*-values after meta-analysis for FA associations with duration of illness in years including sex as a covariate

*Region*	*Z-score*	β*-Value*	*s.e.*	P*-value*
ACR	−4.04	−0.001013	0.00054	0.071
**ALIC**	−**3.77**	−**0.000512**	**0.00041**	**1.66 × 10**^**−9**^
Average FA	−7.08	−0.000476	0.00024	0.0094
**BCC**	−**1.78**	−**0.001170**	**0.00024**	**9.33 × 10**^**−19**^
**CC**	−**3.34**	−**0.000836**	**0.00026**	**7.24 × 10**^**-6**^
**CGC**	−**7.67**	−**0.000761**	**0.00014**	**2.5 × 10**^**−13**^
CGH	−4.58	0.000101	0.00020	0.65
CR	−4.19	−0.000702	0.00019	0.12
CST	−7.64	0.000042	0.00013	0.95
**EC**	−**6.14**	−**0.000528**	**0.00047**	**2.45 × 10**^**−10**^
FX	−0.72	−0.002240	0.00011	0.0003
**FXST**	−**1.90**	−**0.000673**	**0.00010**	**2.26 × 10**^**−5**^
GCC	−3.42	−0.001011	0.00013	0.033
IC	−5.27	−0.000348	0.00045	0.11
IFO	−3.77	−0.000164	0.00063	0.72
PCR	−2.75	−0.000573	0.00008	0.0052
PLIC	−3.60	−0.000222	0.00057	0.4
**PTR**	−**2.82**	−**0.001138**	**0.00011**	**3.03 × 10**^**−7**^
RLIC	−3.87	−0.000584	0.00043	0.024
**SCC**	−**5.02**	−**0.000600**	**0.00019**	**1.29 × 10**^**−8**^
SCR	−2.80	−0.000499	0.00025	0.24
SFO	−3.05	−0.000599	0.00030	0.078
**SLF**	−**6.76**	−**0.000600**	**0.00012**	**2.10 × 10**^**−15**^
**SS**	−**2.37**	−**0.000913**	**0.00009**	**2.62 × 10**^**−24**^
**UNC**	−**0.65**	−**0.000871**	**0.00028**	**5.20 × 10**^**−8**^

Abbreviations: ACR, anterior corona radiata; ALIC, anterior limb of internal capsule; BCC, body of corpus callosum; CC, corpus callosum; CGC, cingulum; CGH, cingulum (hippocampal portion); CR, corona radiata; CST, corticospinal tract; EC, external capsule; FA, fractional anisotropy; FX, fornix; FXST, fornix stria terminalis; GCC, genu of corpus callosum; IC, internal capsule; IFO, inferior fronto occipital fasciculus; PCR, posterior corona radiata; PLIC, posterior limb of internal capsule; PTR, posterior thalamic radiation; RLIC, retrolenticular part of IC; SCC, splenium of corpus callosum; SCR, superior corona radiata; SFO, superior fronto-occipital fasciculus; SLF, superior longitudinal fasciculus; SS, sagittal stratum; UNC, uncinate.

Significant regions (*P*<0.05/25=0.002) are highlighted in bold.
