# Supplementary material for: Widespread white matter microstructural differences in schizophrenia across 4322 individuals: results from the ENIGMA Schizophrenia DTI Working Group
Source: Mol Psychiatry. 2017 Oct 17;23(5):1261–9. doi: 10.1038/mp.2017.170 (PMC5984078; doi:10.1038/mp.2017.170)
Supplement: Supplementary Information [file mp2017170x1.doc]

# Supplementary Information

**Supplementary Note 1**. Further description of ENGMA-DTI protocols

**Supplementary note 2.** Controlling for core and periphery FA

**Supplementary note 3.** Meta-analysis description

**Supplementary Note 4**. Power analysis

**Supplementary Note 5.** Diffusivity analysis

**Supplementary Note 6.** Sex-specific effect sizes: Male/female cases vs controls

**Supplementary Note 7.** Correlation between effect size and ROI size

**Supplementary Note 8**. Acknowledgements and conflicts of interest

**Supplementary Figure 1:** Map of ENIGMA-Schizophrenia DTI sites

**Supplementary Figure 2:** Cohen’s *d* effect sizes, after meta-analysis, for FA differences between individuals with schizophrenia and healthy controls, **covarying for average FA**. Age, sex, age×sex, age^2^ and age^2^×sex, included as covariates. Error bars represent the 95% confidence interval. Orange bars represent significance after Bonferroni correction threshold of 0.05/25 = 0.002.

**Supplementary Figure 3:** Cohen’s *d* effect sizes, after meta-analysis, for FA differences between individuals with schizophrenia and healthy controls, **covarying for core FA**. Age, sex, age×sex, age^2^ and age^2^×sex, included as covariates. Error bars represent the 95% confidence interval. Orange bars represent significance after Bonferroni correction threshold of 0.05/25 = 0.002.

**Supplementary Figure 4:** Cohen’s *d* effect sizes, after meta-analysis, for FA differences between individuals with schizophrenia and healthy controls, **covarying for periphery FA**. Age, sex, age×sex, age^2^ and age^2^×sex, included as covariates. Error bars represent the 95% confidence interval.

**Supplementary Figure 5:** Meta-analysis forest plots for each ROI

**Supplementary Figure 6**. Cohen’s *d* effect sizes, after meta-analysis, for MD differences between schizophrenia patients and healthy controls, including age, sex, age×sex, age^2^ and age^2^×sex, as covariates. Error bars represent the 95% confidence interval. Significant regions (p < 0.05/25 = 0.002) are indicated in orange.

**Supplementary Figure 7.** Cohen’s *d* effect sizes, after meta-analysis, for RD differences between schizophrenia patients and healthy controls, including age, sex, age×sex, age^2^ and age^2^×sex, as covariates. Error bars represent the 95% confidence interval. Significant regions (p < 0.05/25 = 0.002) are indicated in orange.

**Supplementary Figure 8:** **.**Cohen’s *d* effect sizes, after meta-analysis, for AD differences between schizophrenia patients and healthy controls, including age, sex, age×sex, age^2^ and age^2^×sex, as covariates. Error bars represent the 95% confidence interval. Significant regions (p < 0.05/25 = 0.002) are indicated in orange.

**Supplementary Figure 9:** Cohen’s d effect sizes, after meta-analysis, of five tracts showing the largest effect sizes in the overall meta-analysis, for 14 cohorts with a high number of gradient directions (53+) and 15 cohorts with a low number of gradient directions (<32). Error bars represent the 95% confidence interval.

**Supplementary Figure 10:** Cohen’s d effect sizes after meta-analysis for FA differences between schizophrenia patients and healthy controls in females only, including age as a covariate.

**Supplementary Figure 11:** Cohen’s d effect sizes after meta-analysis for FA differences between schizophrenia patients and healthy controls in males only, including age as a covariate.

**Supplementary Figure 12:** Scatter plot illustrating age by diagnosis interaction for FA of the ACR and average FA across the entire skeleton.

**Supplementary Table 1:** DTI acquisition protocols for each site

**Supplementary Table 2:** ENIGMA-Schizophrenia DTI site demographics

**Supplementary Table 3:** ENIGMA-Schizophrenia DTI clinical information

**Supplementary Table 4.** Lateralized results for case/control FA differences

**Supplementary Table 5:** Partial correlation betas (meta b), and p-values for symptom severity as measured by SAPS and SANS scores

**Supplementary Table 6:.** Partial correlation betas (meta b), and p-values for symptom severity as measured by PANSS total and PANSS negative scores

**Supplementary Table 7:** N80: The total number of samples required, per group, to achieve 80% power to detect group differences using a t-test at the threshold of *p*<0.05 (two-tailed).

**Supplementary Table 8:** Intraclass correlation coefficients of FA measures for 19 healthy individuals scanned at baseline, 3 months and 6 months follow-up.

**Supplementary Table 9:** Intraclass correlation coefficients of AD (axial diffusivity) measures for 19 healthy individuals scanned at baseline, 3 months and 6 months follow-up.

**Supplementary Table 10:** Intraclass correlation coefficients of RD (radial diffusivity) measures for 19 healthy individuals scanned at baseline, 3 months and 6 months follow-up.

**Supplementary Table 11:** Intraclass correlation coefficients of MD (mean diffusivity) measures for 19 healthy individuals scanned at baseline, 3 months and 6 months follow-up.

**Supplementary Table 12:** Intraclass correlation coefficients of FA measures for 19 healthy individuals scanned at baseline and 3 months.

**Supplementary Table 13:** Intraclass correlation coefficients of AD (axial diffusivity) measures for 19 healthy individuals scanned at baseline and 3 months.

**Supplementary Table 14:** Intraclass correlation coefficients of MD (mean diffusivity) measures for 19 healthy individuals scanned at baseline and 3 months.

**Supplementary Table 15:** Intraclass correlation coefficients of RD (radial diffusivity) measures for 19 healthy individuals scanned at baseline and 3 months.

**Supplementary Table 16:** Intraclass correlation coefficients of FA measures for 19 healthy individuals scanned at baseline and 6 months.

**Supplementary Table 17:** Intraclass correlation coefficients of AD (axial diffusivity) measures for 19 healthy individuals scanned at baseline and 3 months.

**Supplementary Table 18:** Intraclass correlation coefficients of MD (mean diffusivity) measures for 19 healthy individuals scanned at baseline and 3 months.

**Supplementary Table 19:** Intraclass correlation coefficients of RD (radial diffusivity) measures for 19 healthy individuals scanned at baseline and 3 months.

**Supplementary Table 20:** Intraclass correlation coefficients of FA measures for 19 healthy individuals scanned at 3 months and 6 months after baseline.

**Supplementary Table 21:** Intraclass correlation coefficients of AD (axial diffusivity) measures for 19 healthy individuals scanned at 3 months and 6 months after baseline.

**Supplementary Table 22:** Intraclass correlation coefficients of MD (mean diffusivity) measures for 19 healthy individuals scanned at 3 months and 6 months after baseline.

**Supplementary Table 23:** Intraclass correlation coefficients of RD (radial diffusivity) measures for 19 healthy individuals scanned at 3 months and 6 months after baseline.

**Supplementary Table 24:** Intraclass correlation coefficients for FA measures of N=10 healthy individuals, age 22-28 years, scanned at baseline, 24 hours and 72 hours

**Supplementary Table 25:** Intraclass correlation coefficients for FA measures of N=10 healthy individuals, age 22-28 years, scanned at baseline and 24 hours .

**Supplementary Table 26:** Intraclass correlation coefficients for FA measures of N=10 healthy individuals, age 22-28 years, scanned at baseline and 72 hours .

**Supplementary Table 27:** Intraclass correlation coefficients for FA measures of N=10 healthy individuals, age 22-28 years, scanned at 24 hours and 72 hours after baseline.

**Supplementary Table 28:** Intraclass correlation coefficients for FA measures of N=10 healthy individuals, age 22-28 years, scanned at baseline and 72 hours.

**Supplementary Table 29:** Intraclass correlation coefficients for FA measures of N=10 healthy individuals, age 22-28 years, scanned at 24 hours and 72 hours after baseline.

**Supplementary Note 1: Further detail of ENIGMA-DTI protocols**

Details on study type, scanner and acquisition parameters are provided in Supplementary Table 3.  Preprocessing, including eddy current correction, EPI induced distortion correction, and tensor fitting, was carried out at each site. Image analysis was conducted at each site using tract-based spatial statistics (TBSS) as part of FSL software (Smith et al., 2006).

Individual subject FA maps were aligned to the custom ENIGMA-DTI FA template derived from 400 adult participants scanned across four sites designed for for optimal multi-site harmonization (Jahanshad et al., 2013).  FA voxels were then projected onto the ENIGMA-DTI template skeleton. This creates a unique FA skeleton in the same space for each individual in each cohort. To minimize effects of residual registration misalignment, the regions of interests were consistent in size across sites and the skeletonization procedure was performed individually at each site to minimize any site-specific residual misalignment.

The same projection used for the FA images also projects the non-FA (mean axial and radial) images onto the skeleton. Voxels along the individual skeletons were averaged across white matter ROIs. A total of 25 bilateral ROIs were delineated based on the JHU WM atlas, an established WM parcellation derived using deterministic tractography (Hua et al, 2008). A whole-brain WM skeleton was defined according to the tract-based spatial statistic methodology (Smith et al, 2006) and ROI-averaged measures of FA, MD, AD and RD were then calculated by averaging each of these voxel measures over all skeleton voxels encapsulated by a particular ROI. This ensured that voxels at the periphery of a fiber bundle, where residual registration misalignment is typically maximal, were excluded from the ROI average. In other words, ROI averaging was performed based on the core of each fiber bundle, as defined by the WM skeleton.

The multi-subject JHU white matter parcellation atlas (Mori et al., 2008) was used to parcellate regions of interest from the ENIGMA template in MNI space. As certain ROIs may be susceptible to the effects of field of view (FOV) and partial voluming, leading to unstable and unreliable estimates, a reliability analysis based on multiple cohorts with longitudinal data was conducted for each ROI (see supplementary material).

A total of twenty-five bilateral white matter ROIs were extracted from the skeletonized FA images and averaged. Table 1 lists 25 ROIs (some partially overlapping) that were extracted from the skeletonized images, including 5 midsagittal regions (no lateralized components), and 19 lateralized regions (left and right are averaged to obtain bilateral FA) (Supplementary material). The overall average FA values were calculated by averaging values for the entire white matter skeleton.

ENIGMA-DTI QA/QC protocol consists of visual inspection of the images before and after registration to the ENIGMA template, as well as calculating the average skeleton projection distance.  The distance of voxel projection to the ENIGMA skeleton can assess the registration quality between individual images and ENIGMA-DTI template. Higher projection distance may indicate problems with aligning individual brain to the template. After ROI extraction, histograms of FA and diffusivity measures are computed for each ROI.

| Abbreviation | Full tract name |
| --- | --- |
| AverageFA | Full skeleton average FA |
| ACR (L+R) | Anterior *corona radiata* |
| ALIC (L+R) | Anterior limb of internal capsule |
| BCC | Body of *corpus callosum* |
| CC (BCC+GCC+SCC) | Corpus callosum |
| CGC (L+R) | Cingulum (cingulate gyrus) |
| CGH (L+R) | Cingulum (hippocampal portion) |
| CR (L+R) | *Corona radiata* |
| CST (L+R) | Corticospinal tract |
| EC (L+R) | External capsule |
| FX | *Fornix* |
| FXST (L+R) | *Fornix* (cres) / *Stria terminalis* |
| GCC | ***Genu*** of *corpus callosum* |
| IC (L+R) | Internal capsule |
| IFO (L+R) | Inferior fronto-occipital fasciculus |
| PCR (L+R) | Posterior *corona radiata* |
| PLIC (L+R) | Posterior limb of internal capsule |
| PTR (L+R) | Posterior thalamic radiation |
| RLIC (L+R) | Retrolenticular part of internal capsule |
| SCC | ***Splenium*** of *corpus callosum* |
| SCR (L+R) | Superior *corona radiata* |
| SFO (L+R) | Superior fronto-occipital fasciculus |
| SLF (L+R) | Superior longitudinal fasciculus |
| SS (L+R) | Sagittal *stratum* |
| UNC (L+R) | *Uncinate* fasciculus |

**Supplementary Figure 1.** Map of ENIGMA-Schizophrenia DTI contributing sites. For Australia, ASRB sites should also include Brisbane, Newcastle and Perth.

**Supplementary Note 2: Covarying for core and periphery FA**

The average FA is the FA averaged across the entire white matter skeleton, excluding gray matter.  The white matter skeleton  is then subdivided into a core region and a periphery region. Here, we defined the “core” as the region within the skeleton that was labeled by the JHU white matter atlas, the rest of the skeleton was defined as the “periphery” or non-JHU, and included more than just the edges of the brain. In fact, within the standard, full FOV, ENIGMA-DTI template, the average FA is made up of 112,889 voxels, and the core consists of 31,742 voxels, less than a third of the average FA. The remaining 81,147 voxels surrounding the core comprise the periphery (non-JHU).

To tease apart regional WM effects from the global differences, a post-hoc analysis was conducted in individual tract ROIs (as previously performed) but additionally covarying for 1) average FA across the entire skeleton , 2) average FA within the core of the skeleton only, within which ROIs are defined, or 3) the average FA within the periphery of the skeleton only - areas of the skeleton not included in the ROIs of the JHU atlas.

Core FA was calculated by computing the weighted average of all non-overlapping ROIs. Periphery FA was calculated by subtracting weighted core FA (N core voxels/N periphery voxels x core FA) from the weighted average FA voxels (N average FA voxels/N periphery FA voxels x averageFA).

**Supplementary Note 3: Meta-analysis description**

Using this a random-effects inverse-variance weighted meta-analysis, results from statistical tests performed at each individual site were combined and the overall statistical effect size was calculated as the weighted average of the effects from the individual cohorts as determined by the standard error of the effect.

**Supplementary Note 4: Power analysis**

A post-hoc power analysis, conducted using G*Power v3.1, revealed that the current sample of 1,963 individuals with schizophrenia and 2,359 healthy controls achieved 80% power to detect Cohen’s *d* effect sizes as small as *d*=0.086 at the standard alpha level of *p*<0.05 (two-tailed), and 80% power to detect Cohen’s *d* effect sizes of *d*=0.12 at the study’s Bonferroni-corrected threshold of *p*<0.002. Across all ROIs, we estimated *N*80: the total number of samples required, per group, to achieve 80% power to detect group differences using a t-test at the threshold of *p*<0.05 (two-tailed). *N*80 ranged from 90-6,281 (See Supplementary Table 5).

**Supplementary Note 5: Diffusivity analysis**

Available diffusivity images, including mean, radial and axial diffusivity, were skeletonized and relevant ROI information was extracted according to the ENIGMA-DTI template. A random effects, inverse variance weighted meta-analysis was conducted to combine results. A random effects, inverse variance weighted meta-analysis was conducted to combine results.

**Supplementary Note 6: Sex-Specific Effect Sizes: Male / Female Cases vs Controls**

We observed significant differences in effect size when analyzing males and females separately, with females showing significantly larger effects for decreased FA (paired t=-3.98 p=0.001).

For females (N=1090 controls and 671 schizophrenia patients), 20 of the 25 ROIs showed significantly lower FA for patients at the p=0.002 significance threshold (See Supplementary Figure 9).

For males (N = 1296 controls and 1292 schizophrenia patients) 14 of the 25 ROIs showed significantly decreased FA for patients at the p=0.002 significance threshold (See Supplementary Figure 10).

**Supplementary Note 7: ROI size and effect size**

To investigate if effect sizes are increased for larger ROIs as a result of improved signal, we correlated the square root of the number of voxels with the Cohen’s d effect size of each ROI (see graph below). Excluding average FA of the whole skeleton, we found a medium but non-significant correlation between effect size and ROI size (r=-0.39, p < 0.05). Although the correlation wasn’t significant, caution should be taken when interpreting small or non-significant effects for smaller ROIs as this may be a result of poor signal. When average FA of the whole skeleton is included, the correlation becomes significant (r=0.43, p=0.036).

**
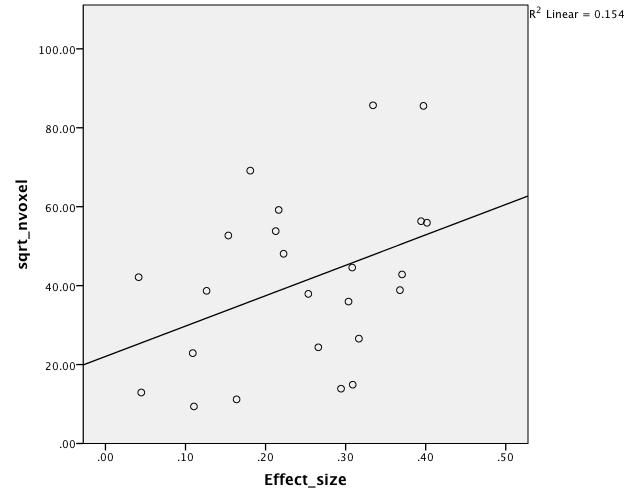
**

**Pearson correlation between ROI size (square root of the number of voxels) and Cohen’s d effect size**

**Supplementary Note 8: Acknowledgements and conflicts of interest**

The **ENIGMA-Schizophrenia working** group gratefully acknowledges support from the NIH BD2K award, U54EB020403.

**TOP:** The TOP study was supported by the Research Council of Norway (#213837, #223273, #229129), South-East Norway Health Authority (#2013-123), South-Eastern Norway Regional Health Authority (#2014097) and KG Jebsen Foundation. The research leading to these results has received funding from the European Community's Seventh Framework Programme (FP7/2007-2013) under grant agreement n° 602450 (IMAGEMEND).

**Osaka:** Some computations were performed at the Research Center for Computational Science, Okazaki, Japan.

**Brain and Mind Research Institute:** This study was supported by the following National Health and Medical Research Council funding sources: Programme Grant (no. 566529), Centres of Clinical Research Excellence Grant (no. 264611), Australia Fellowship (no. 511921) and Clinical Research Fellowship (no. 402864).

**Zora Kikinis (CIDAR)**: Work supported by grant R21MH106793

**Paul Klauser** **(ASRB)** was supported by the Swiss National Science Foundation (SNSF) and the Swiss Society for Medicine and Biology Scholarships (148384) and the National Center for Competence in Research — SYNAPSY — funded by the SNSF.

**Robert W. McCarley** is upported by NIMH 5P50MH080272

**Edinburgh:** Supported by Wellcome Trust 104036/Z/14/Z and by Sackler Foundation

**Raquelle I. Mesholam-Gately** Supported by NIMH 5P50MH080272

**Roberto Roiz** “We thank IDIVAL Neuroimaging Unit for its help in the technical execution of this work”

**Peter Savadjiev** was supported by NARSAD Young Investigator Award Number 22591, from the Brain and Behavior Research Foundation.

**Ulrich Schall** was supported by the National Health & Medical Research Council of Australia and received infrastructure funding of the New South Wales State Government through the Hunter Medical Research Institute and the Schizophrenia Research Institute.

**Martha E. Shenton (CIDAR)** is supported by VA Merit Award (I01 RX00928)

**Gianfranco Spalletta (Rome)** was funded by the Italian Ministry of Health grant RC 12-13-14-15/A

**FBIRN** This work was supported by the National Center for Research Resources at the National Institutes of Health (grant numbers: NIH 1 U24 U24 RR021992 (Function Biomedical Informatics Research Network), NIH 1 U24 RR025736-01 (Biomedical Informatics Research Network Coordinating Center; http://www.birncommunity.org), and he National Institute Of Biomedical Imaging And Bioengineering (NIBIB) of the National Institutes of Health under Award Number U54EB020403. The funding sources had no role in the study design, data collection, analysis, and interpretation of the data. We are thankful to Liv McMillan for overall study coordination, Harry Mangalam, Joseph Farran, and Adam Brenner, for administering the University of California, Irvine High-Performance Computing cluster, and to the research subjects for their participation.

**Esther Goudzwaard (FBIRN)** was supported by the following Dutch Foundations: A.S.C. Academy, Amsterdam Universiteitsfonds, Bekker-la Bastide-Fonds, Schimmel Schuurman - van Outeren Foundation, Scholten-Cordes Foundation.

Drs Pacigo, Xie, Hyde, Chen, O’Donnell and Hibar have private funding unrelated to the content of this paper.

**
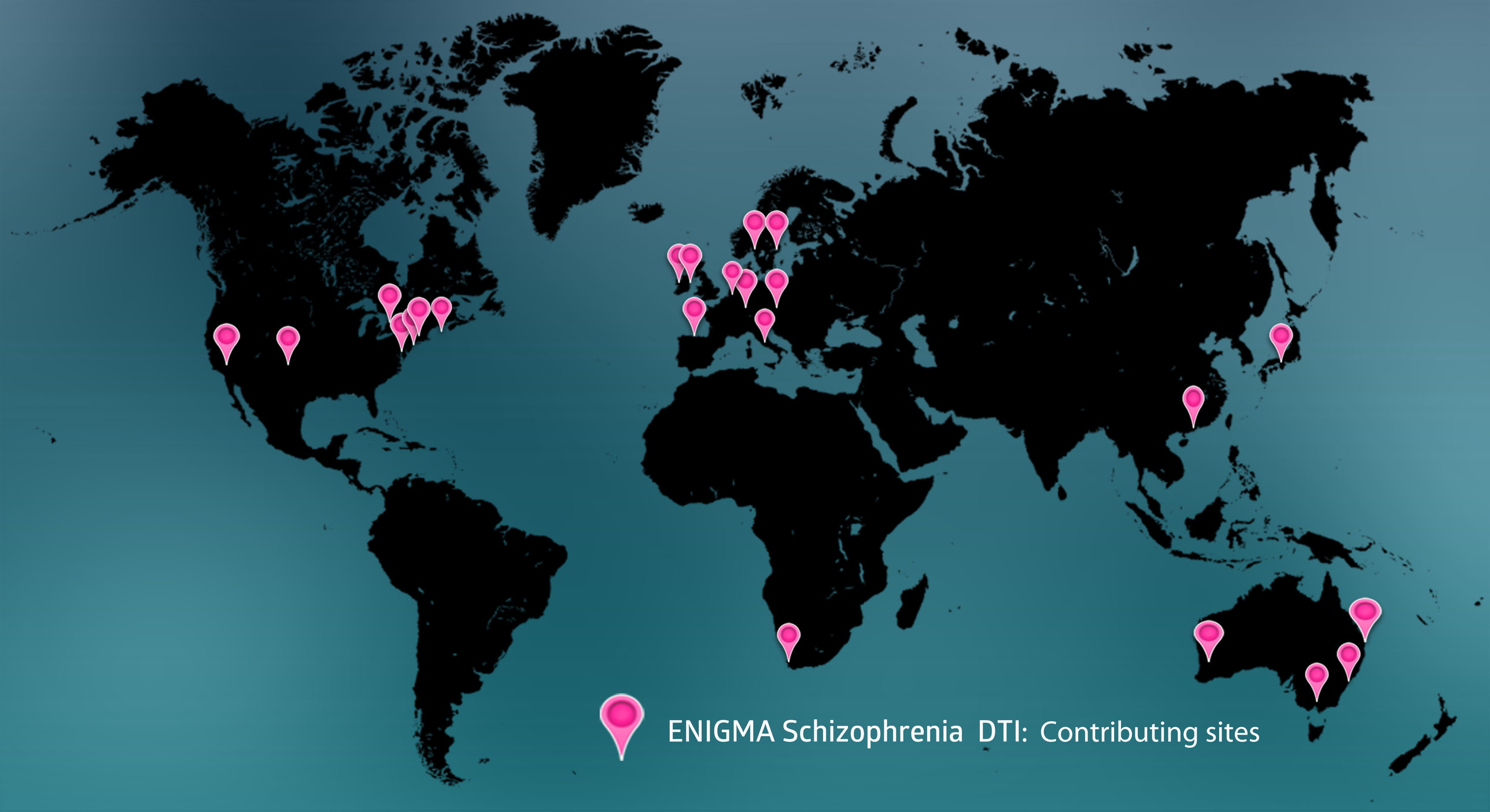
**

**Supplementary Figure 1**: Map of ENIGMA-Schizophrenia DTI participating sites

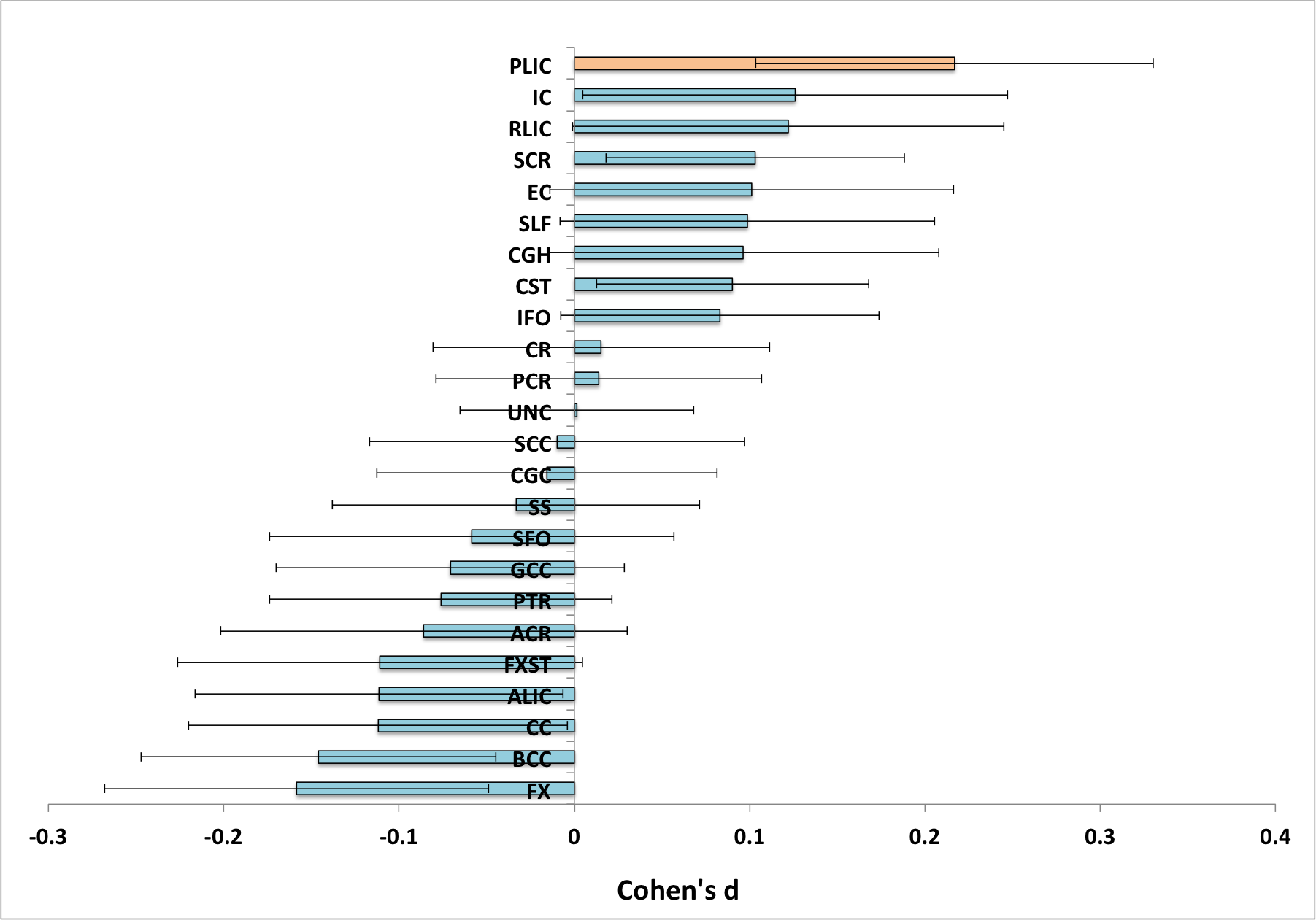


**Supplementary Figure 2.** Cohen’s *d* effect sizes, after meta-analysis, for FA differences between individuals with schizophrenia and healthy controls, **covarying for average FA**. Age, sex, age×sex, age^2^ and age^2^×sex, included as covariates. Error bars represent the 95% confidence interval. Orange bars represent significance after Bonferroni correction threshold of 0.05/25 = 0.002.


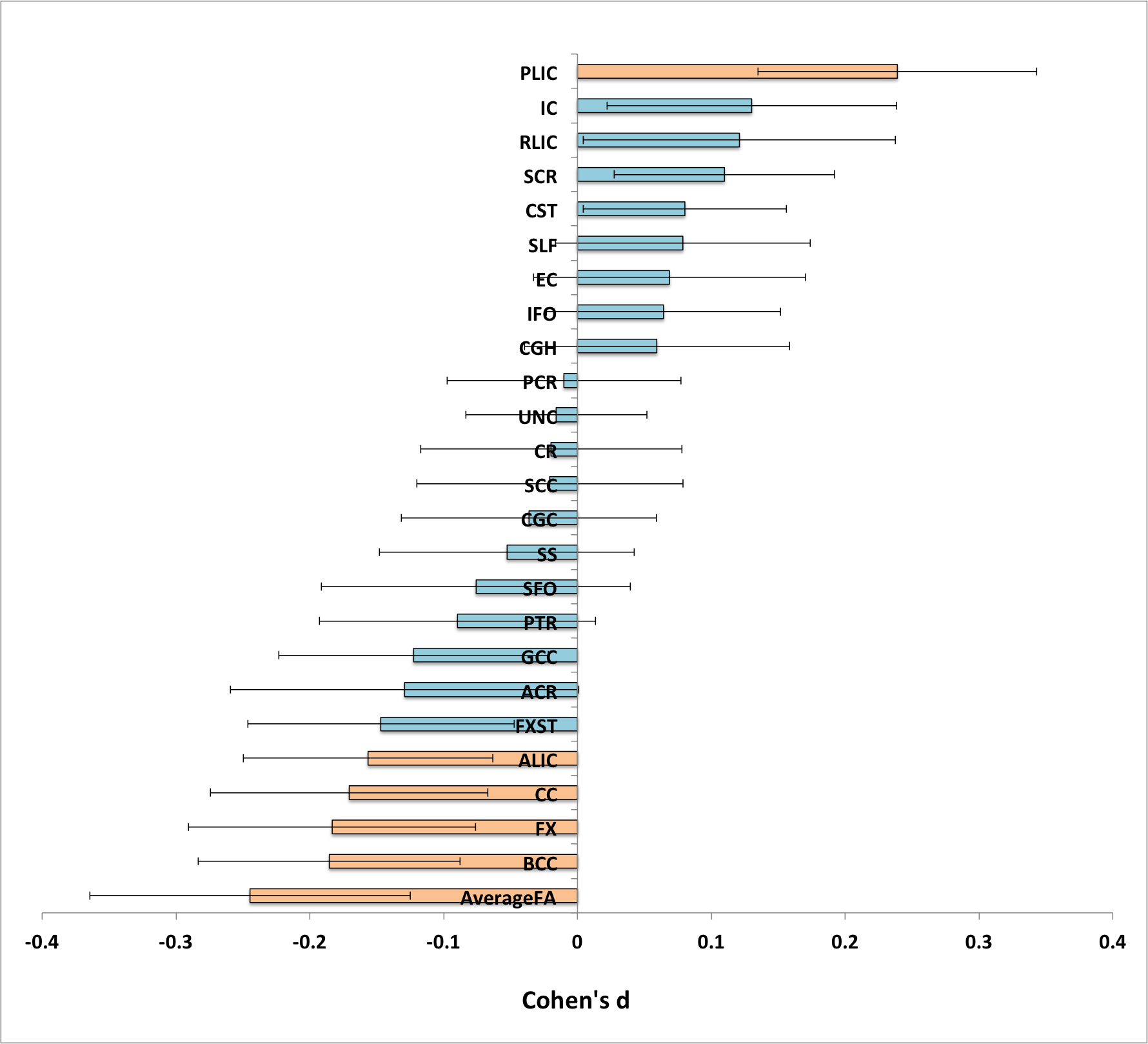


**Supplementary Figure 3.** Cohen’s *d* effect sizes, after meta-analysis, for FA differences between individuals with schizophrenia and healthy controls, **covarying for core FA**. Age, sex, age×sex, age^2^ and age^2^×sex, included as covariates. Error bars represent the 95% confidence interval. Orange bars represent significance after Bonferroni correction threshold of 0.05/25 = 0.002.


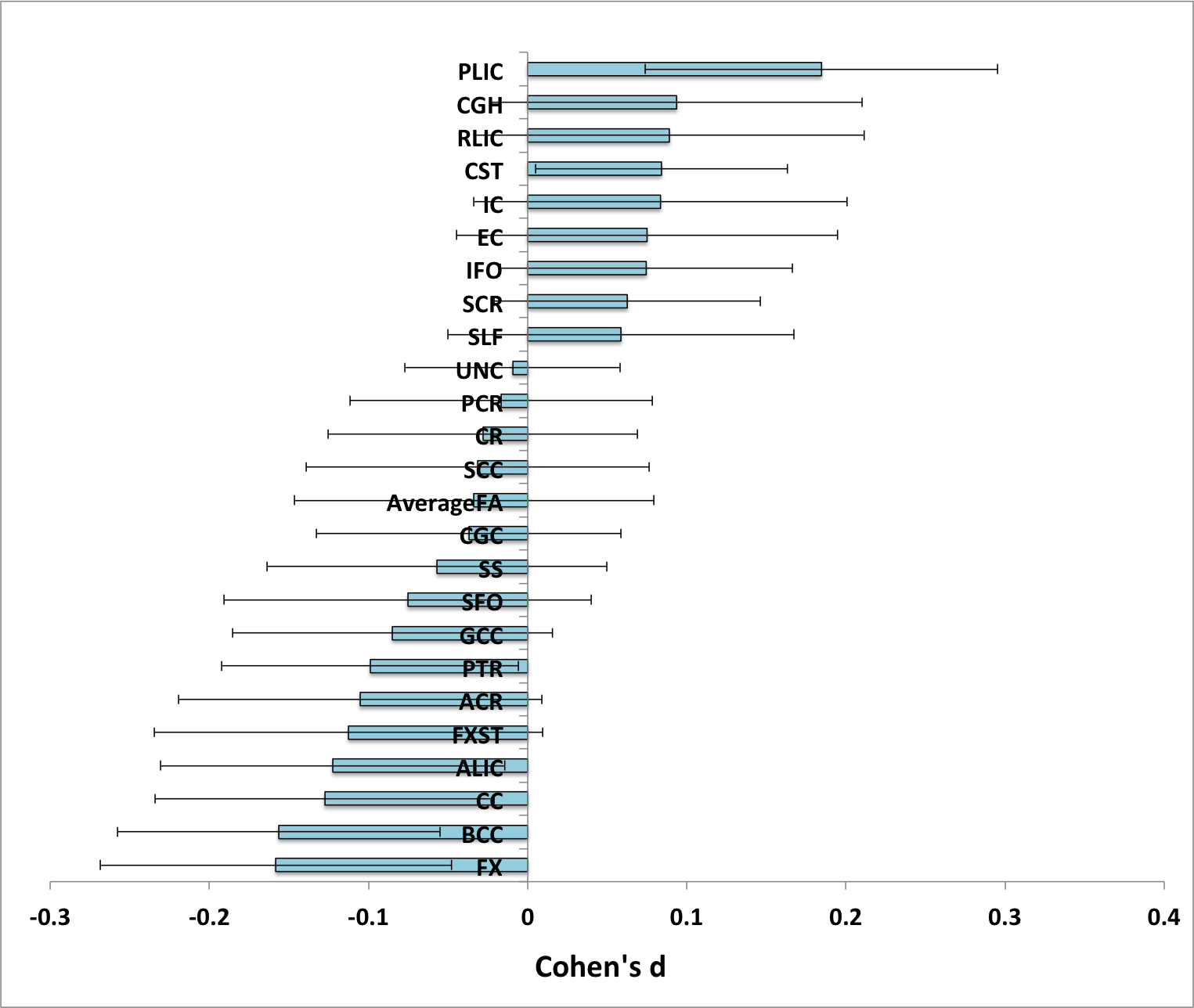


**Supplementary Figure 4.** Cohen’s *d* effect sizes, after meta-analysis, for FA differences between individuals with schizophrenia and healthy controls, **covarying for periphery FA**. Age, sex, age×sex, age^2^ and age^2^×sex, included as covariates. Error bars represent the 95% confidence interval.

**Supplementary Figure 5. Meta-analysis forest plots for each ROI**


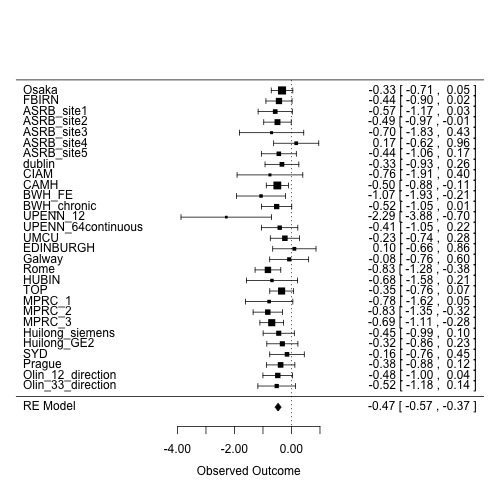

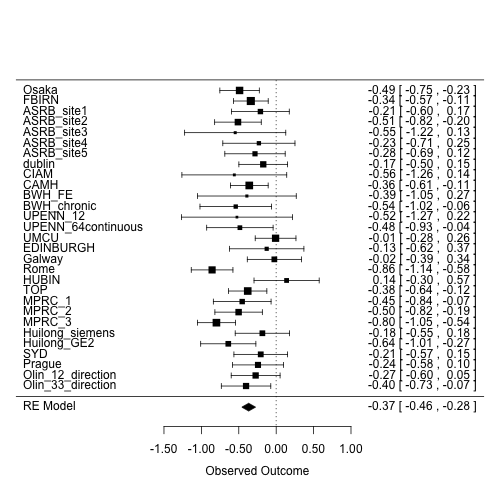


**ACR ALIC**


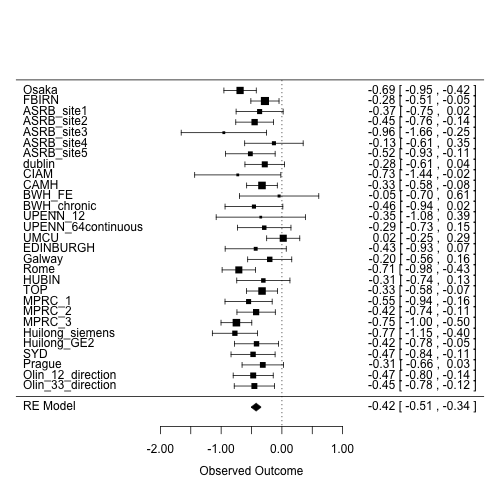

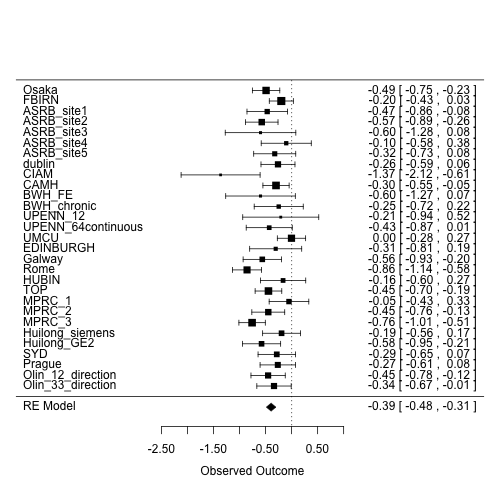


**Average FA BCC**


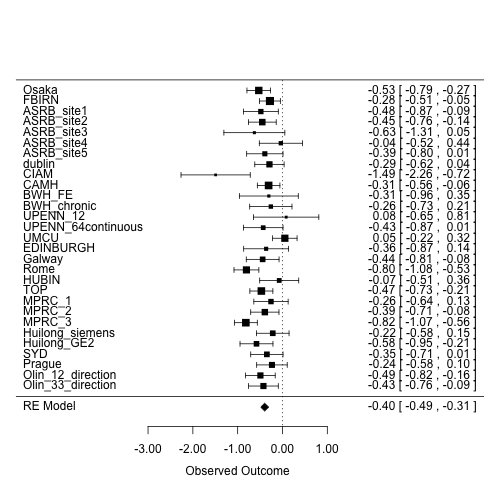

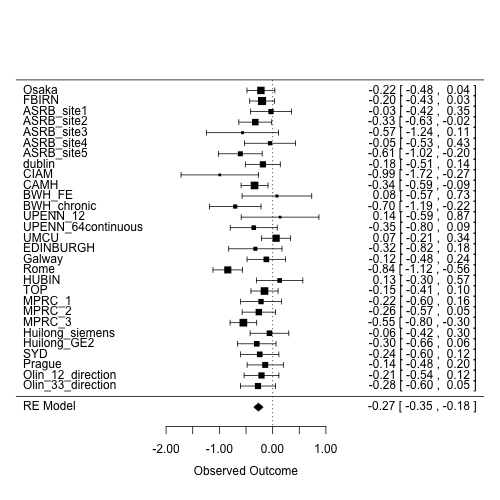


**CC CGC**


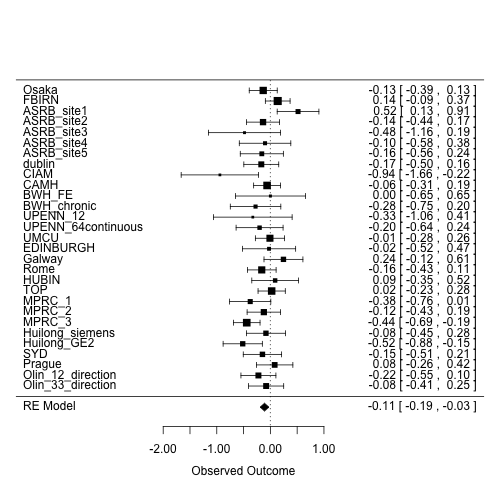

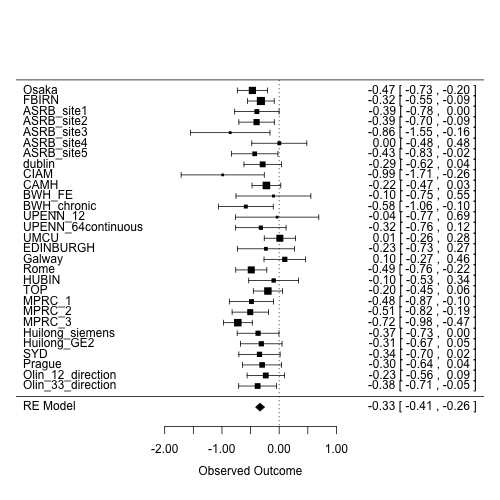


**CGH CR**


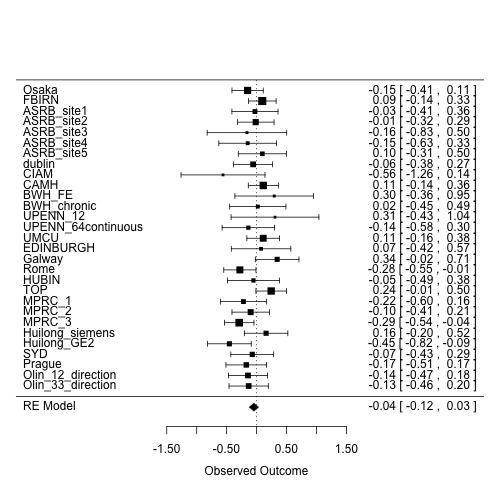

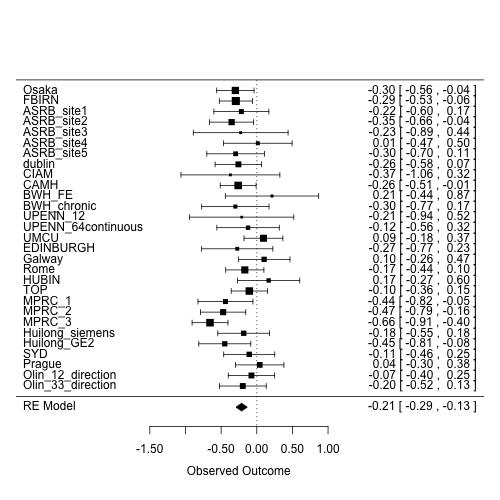


**CST EC**


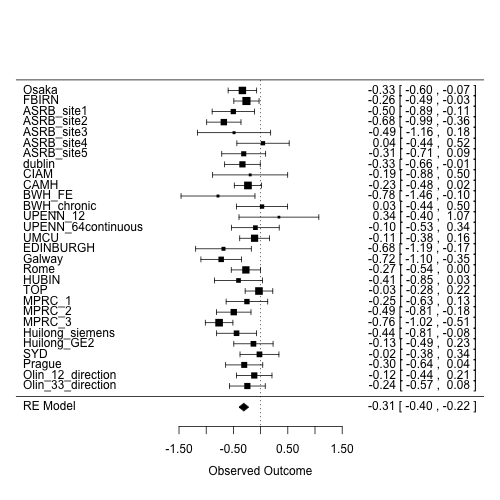

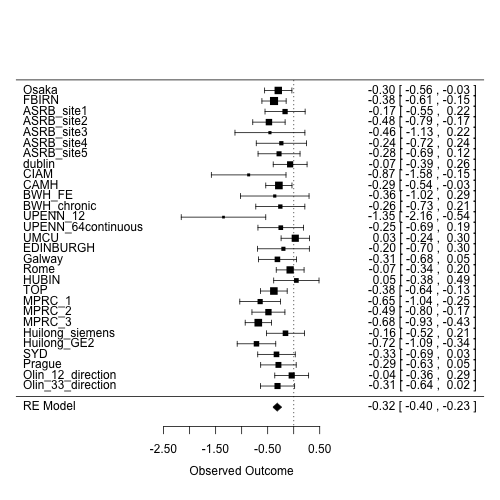


**FX FXST**


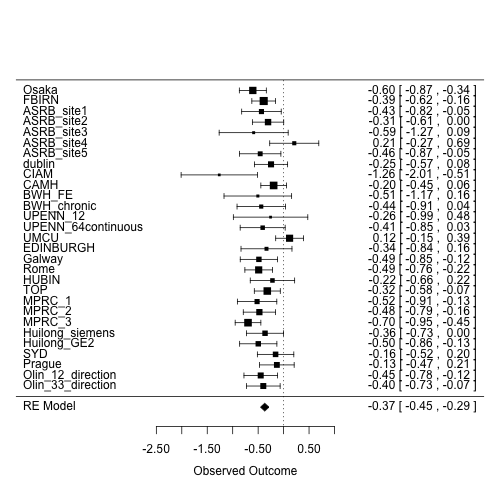

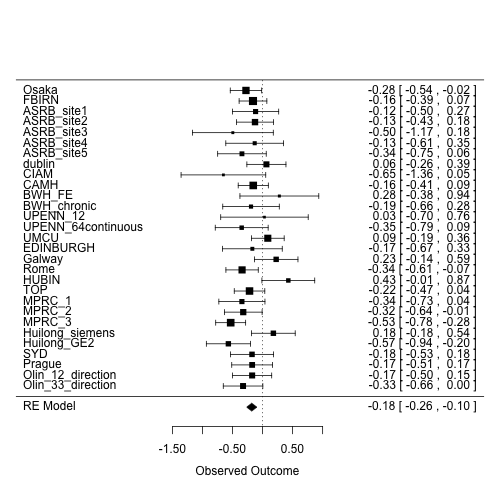


**GCC IC**


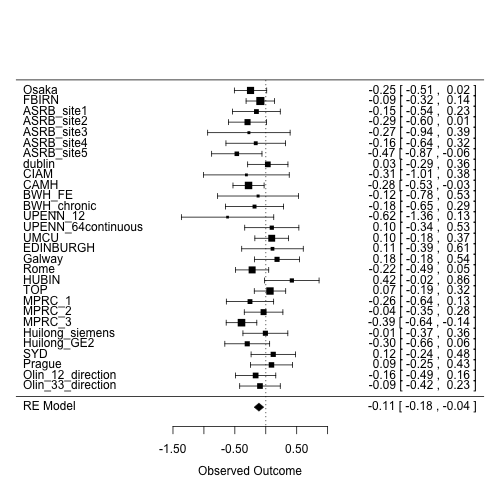

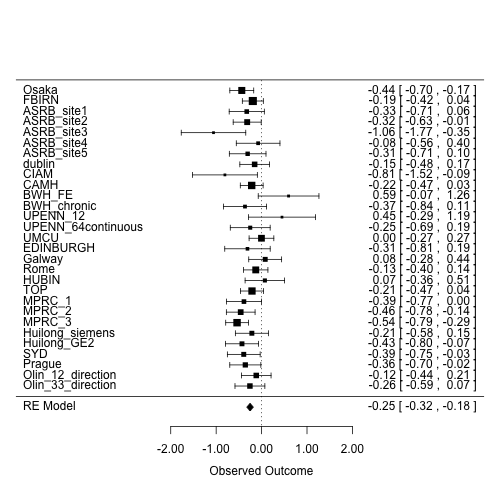


**IFO PCR**


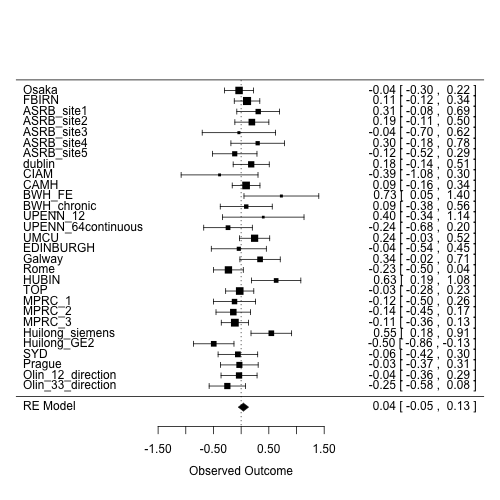

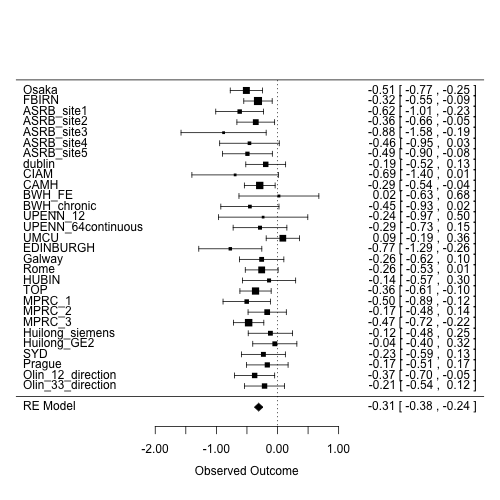


**PLIC PTR**


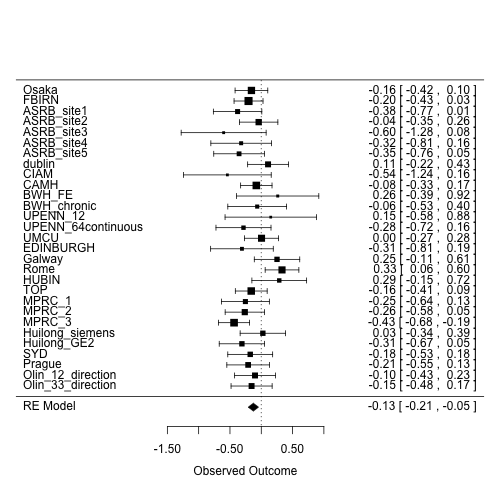

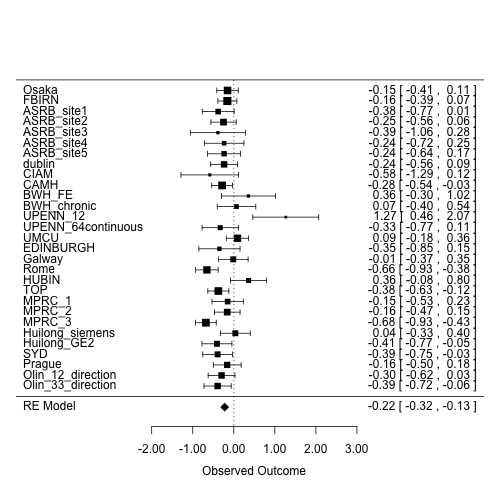


**RLIC SCC**


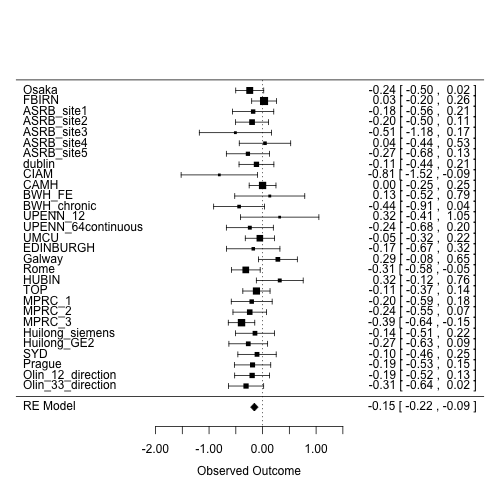

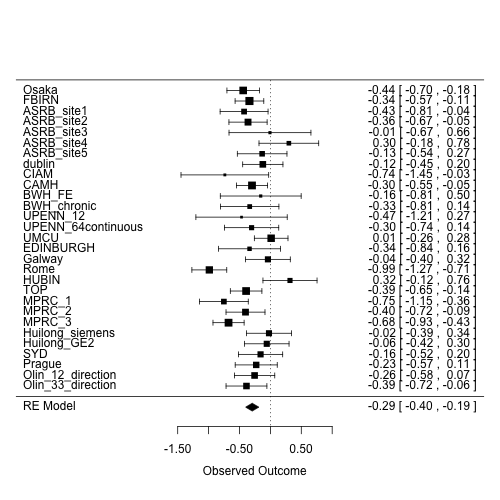


**SCR SFO**

**
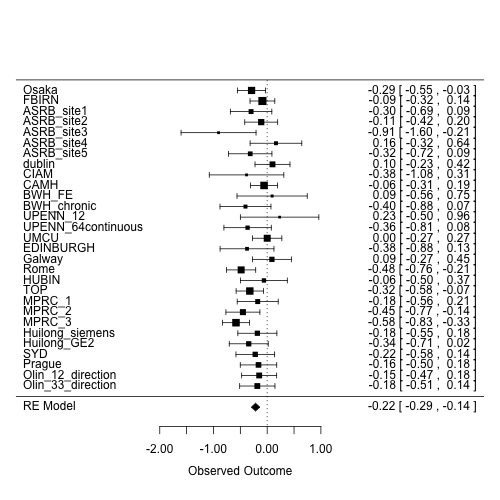

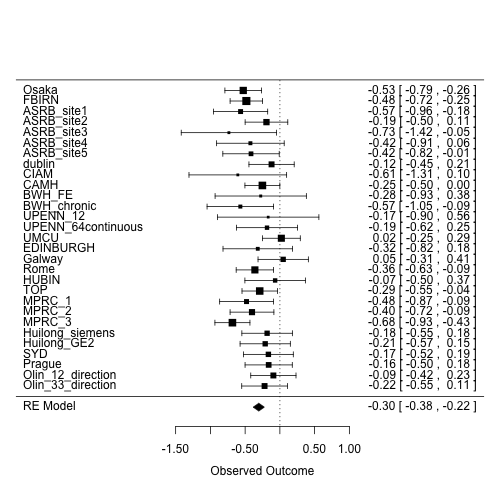
**

**SLF SS**

**
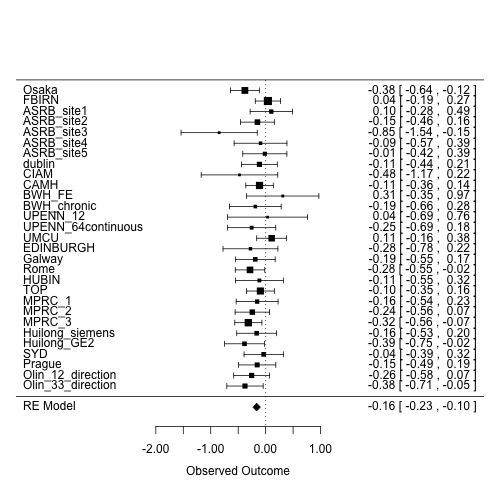
**

**UNC**


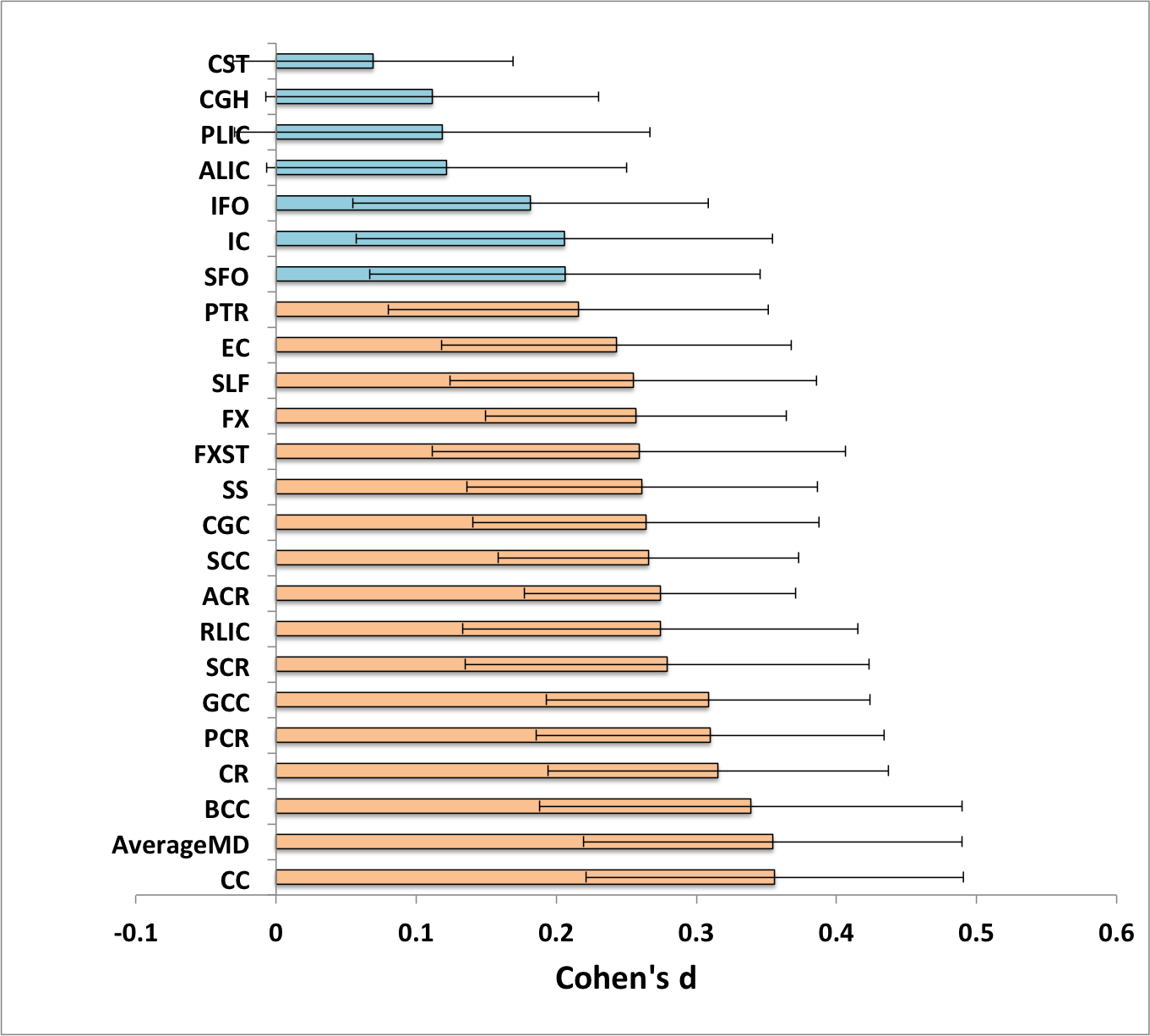


**Supplementary Figure 6**. Cohen’s *d* effect sizes, after meta-analysis, for MD differences between schizophrenia patients and healthy controls, including age, sex, age×sex, age^2^ and age^2^×sex, as covariates. Error bars represent the 95% confidence interval. Significant regions (p < 0.05/25 = 0.002) are indicated in orange.


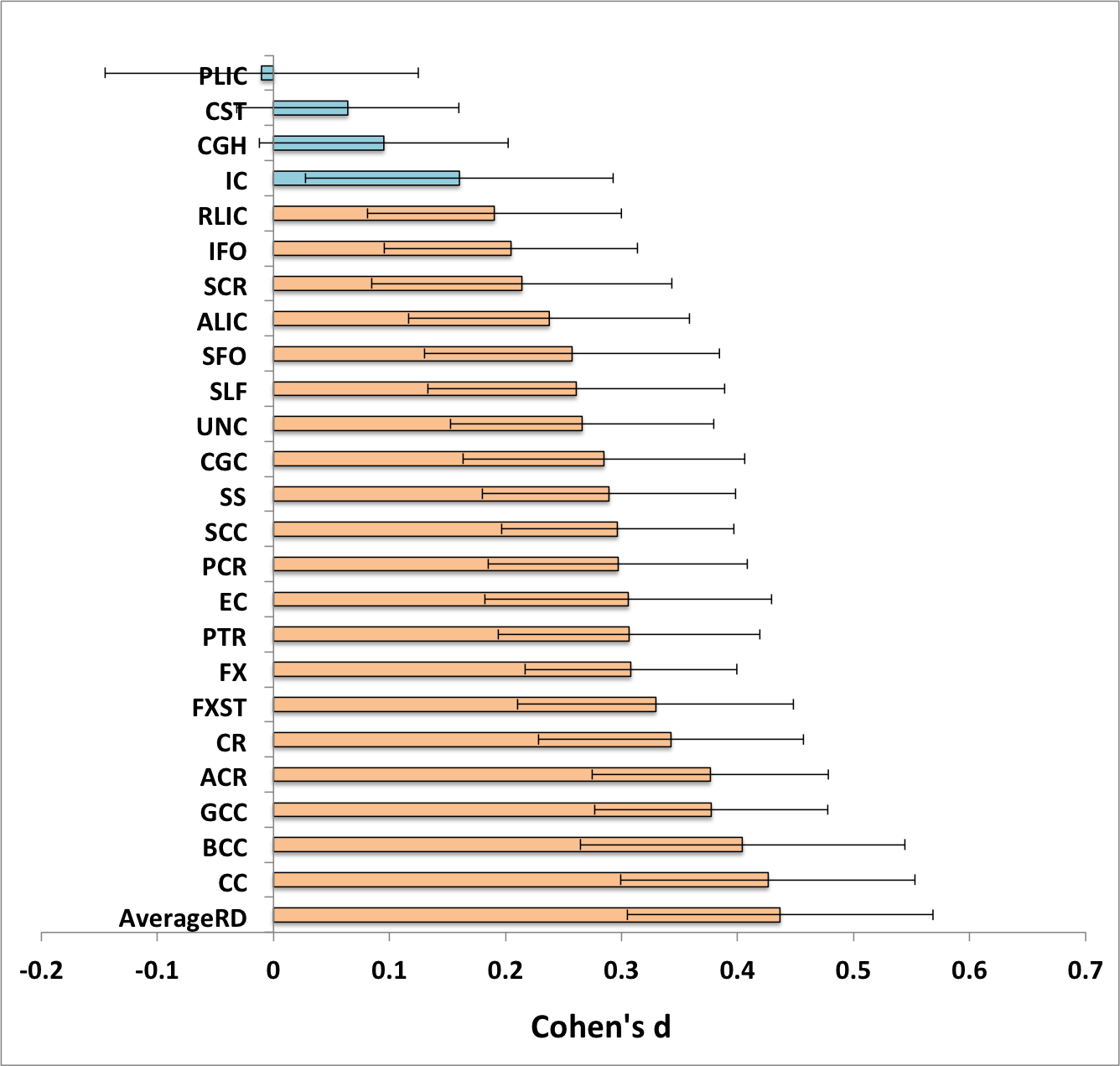


**Supplementary Figure 7.** Cohen’s *d* effect sizes, after meta-analysis, for RD differences between schizophrenia patients and healthy controls, including age, sex, age×sex, age^2^ and age^2^×sex, as covariates. Error bars represent the 95% confidence interval. Significant regions (p < 0.05/25 = 0.002) are indicated in orange.


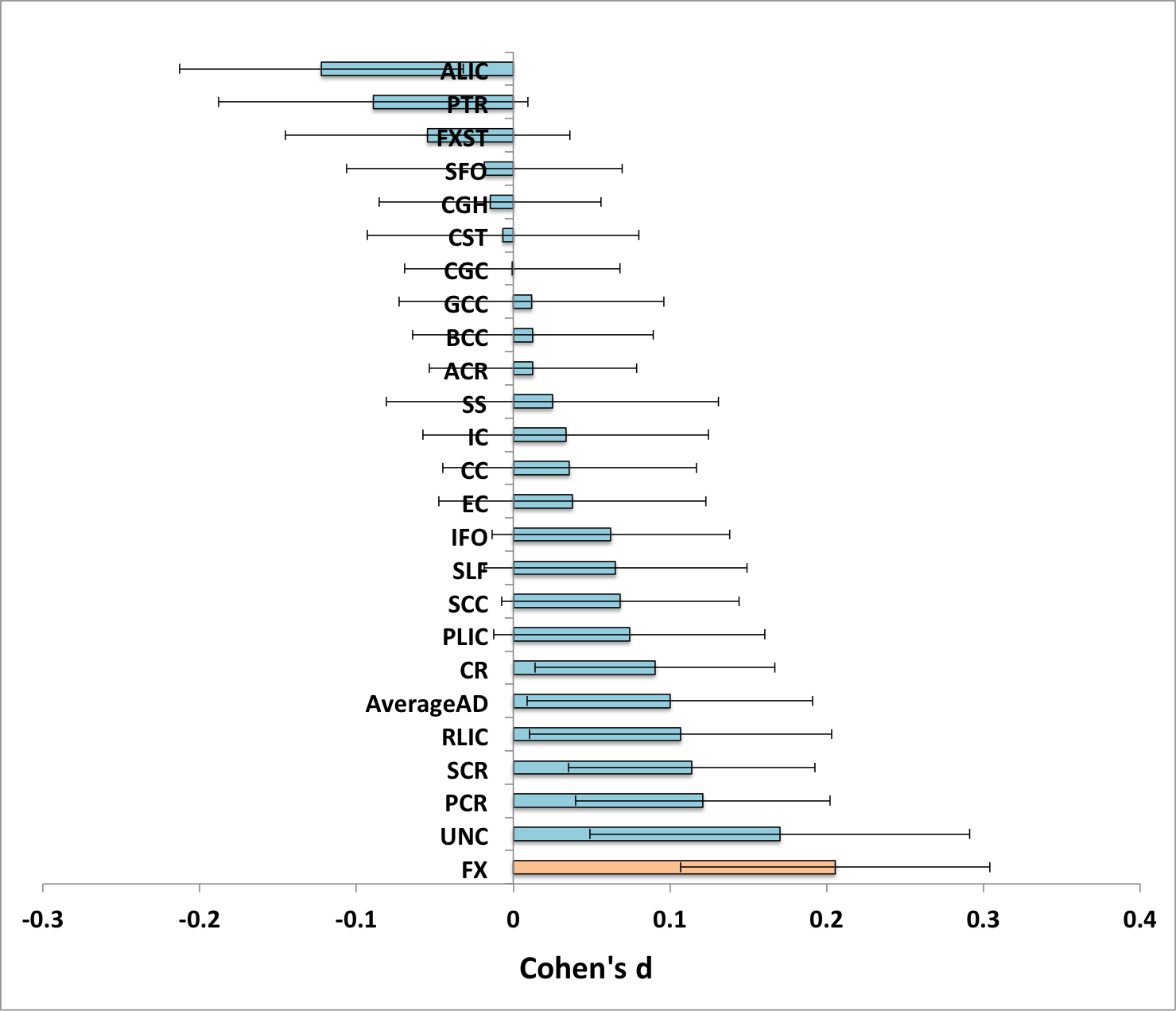


**Supplementary Figure 8.** Cohen’s *d* effect sizes, after meta-analysis, for AD differences between schizophrenia patients and healthy controls, including age, sex, age×sex, age^2^ and age^2^×sex, as covariates. Error bars represent the 95% confidence interval. Significant regions (p < 0.05/25 = 0.002) are indicated in orange.


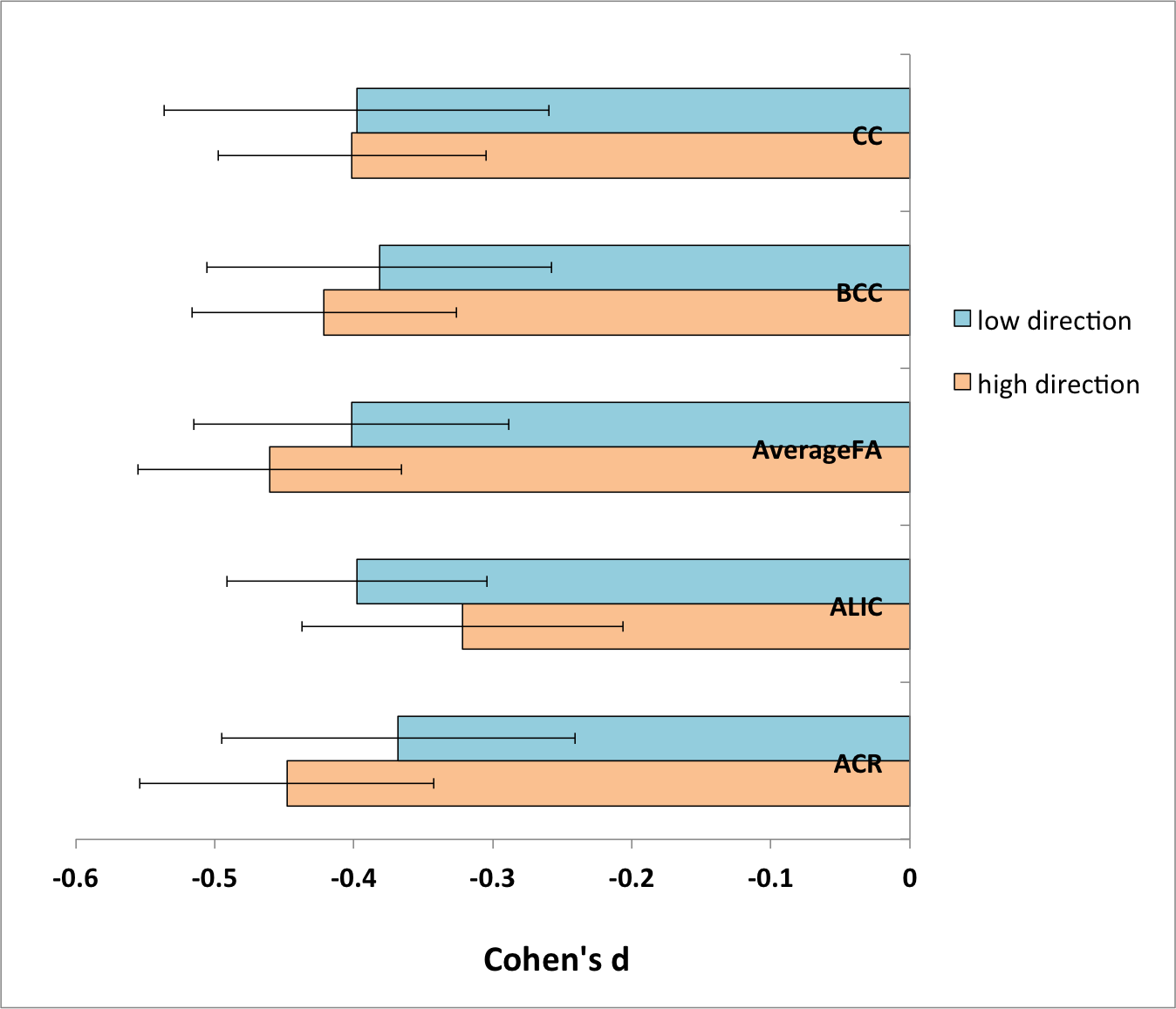


**Supplementary Figure 9:** Cohen’s d effect sizes, after meta-analysis, for FA differences between patients and controls, of five tracts showing the largest effect sizes in the overall meta-analysis, for 14 cohorts with a high number of gradient directions (53+) and 15 cohorts with a low number of gradient directions (<32). Error bars represent the 95% confidence interval.


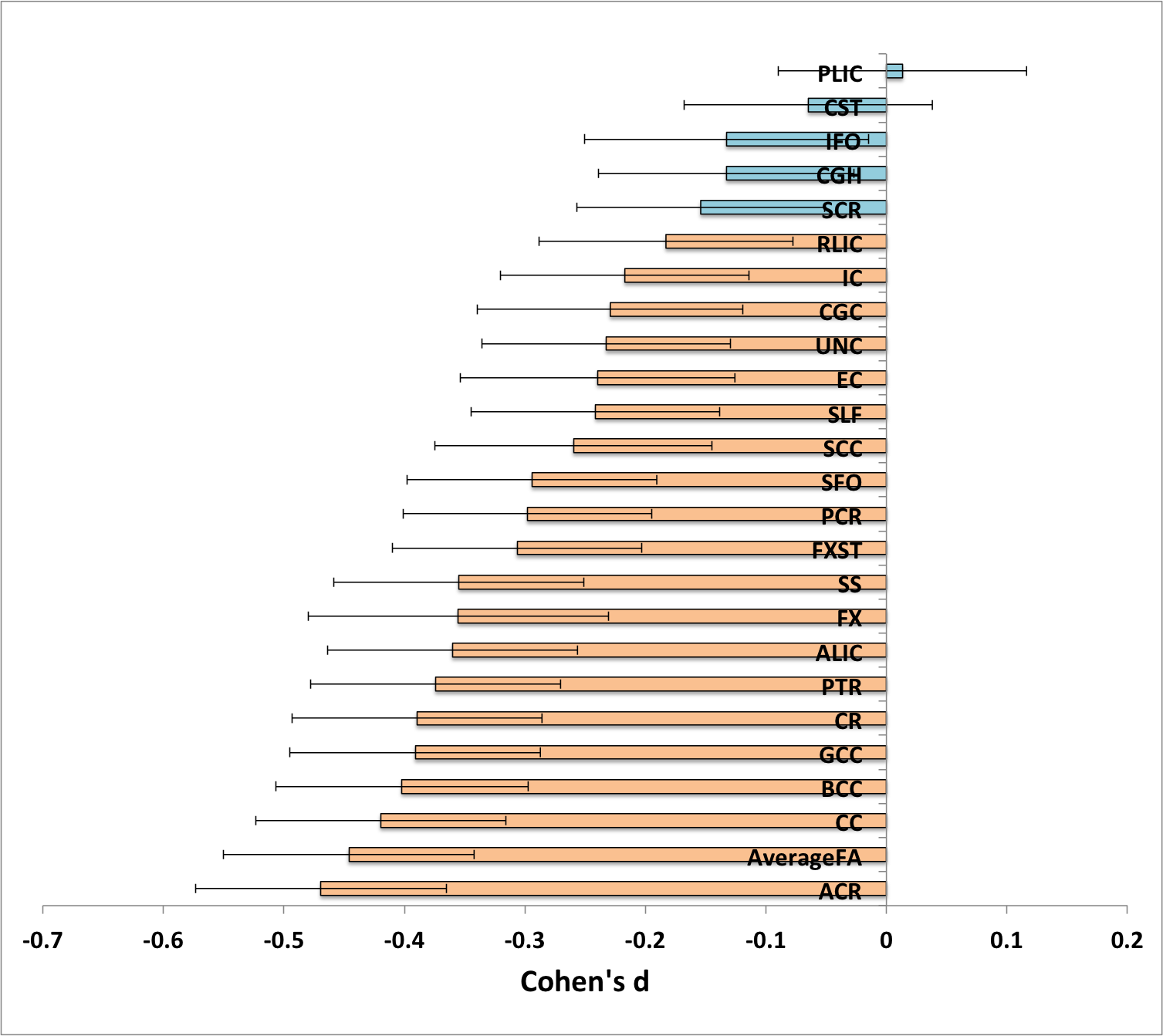


**Supplementary Figure 10:** Cohen’s d effect sizes after meta-analysis for FA differences between schizophrenia patients and healthy controls in females only, including age as a covariate

**
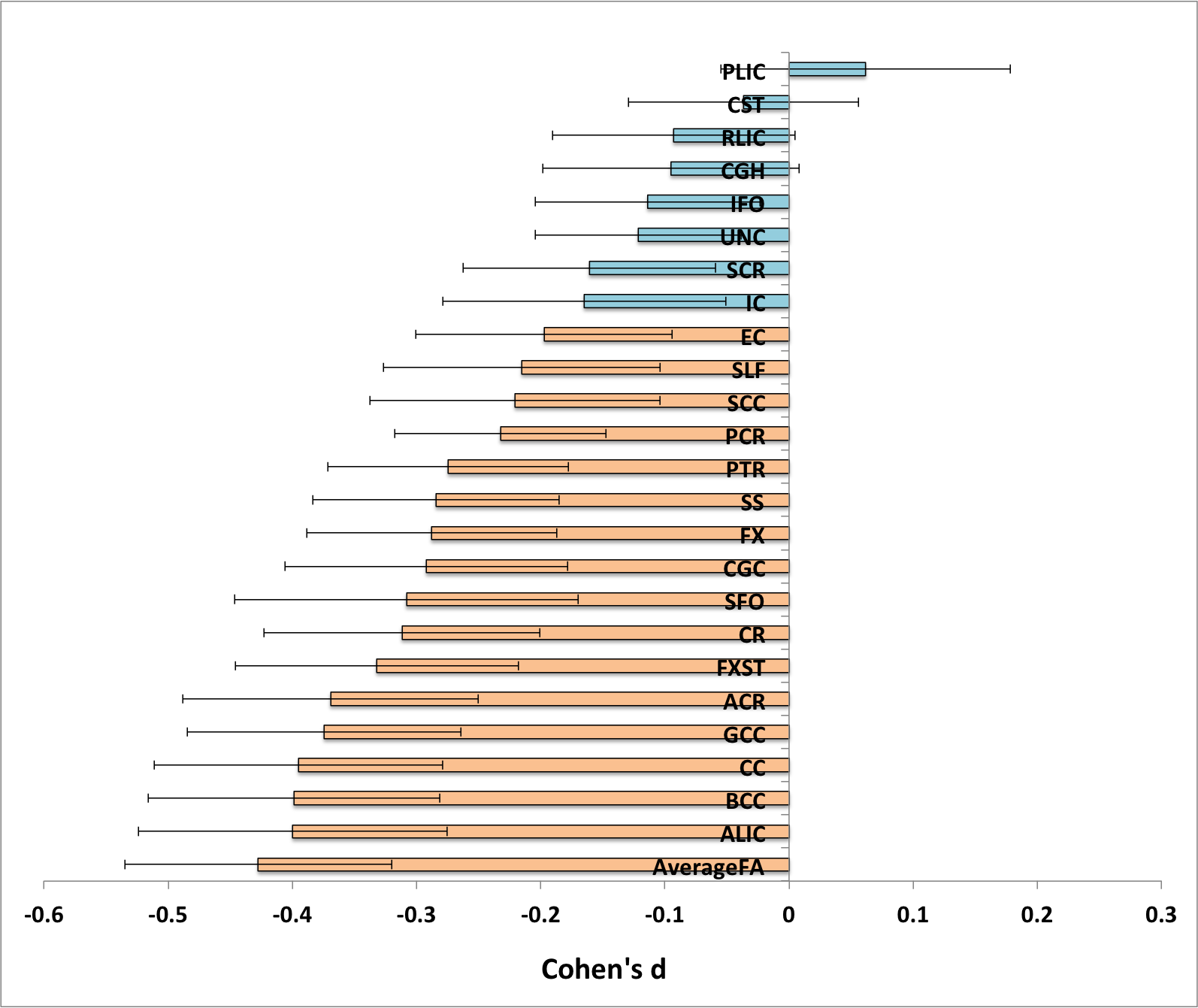
**

**Supplementary Figure 11:** Cohen’s d effect sizes after meta-analysis for FA differences between schizophrenia patients and healthy controls in males only, including age as a covariate


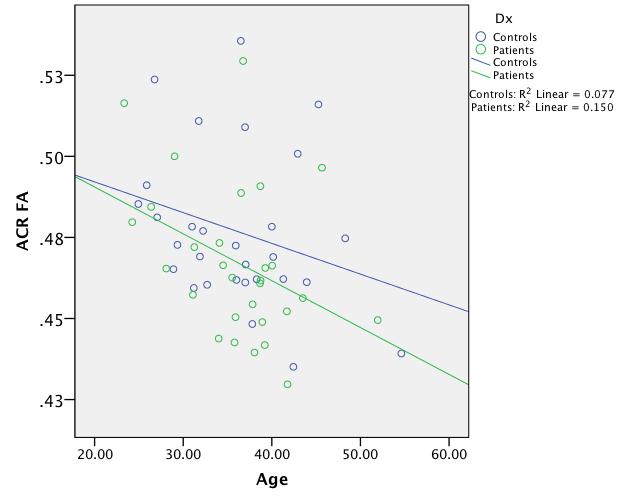

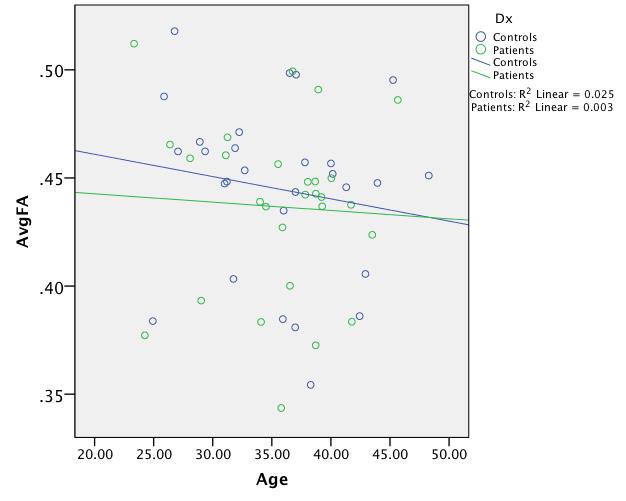


**Supplementary Figure 12:** Scatter plot illustrating age by diagnosis interaction for FA of the ACR and average FA across the entire skeleton.

| Study Cohort | Scanner | Field strength | Acquisitions | Voxel size and slice thickness | Gradient directions and b-value (mm/s2) | b=0 scans |
| --- | --- | --- | --- | --- | --- | --- |
| **ESO study (Prague)** | Siemens TRIO | 3T | 2 | 2x 2×2 mm | 30 at b=900 | 2 |
| **Osaka** | GE | 3T | 1 | 1.0156x1.0156x33.0 mm | 15 at b = 1000 | 1 |
| **HUBIN** | GE | 3T | 1 | 0.94 x 0.94 x 2.90 mm | 60 at b=1000 | 10 |
| **TOP** | GE | 3T | 1 | 1.875 x 1.875 x 2.5 mm | 30 at b=1000 | 1 |
| **Brain and Mind Research Institute (SYD)** | GE MR750 | 3T | 1 | 0.9x2.0x2.0 mm | 69 at b=1159 | 8 |
| **Olin(12 direction)** | Siemens | 3T | 1 | 1.56x1.56x3 mm | 12 at b=1000 | 1 |
| **Olin(32 direction)** | Siemens | 3T | 1 | 1.7x1.7x3 mm | 32 at b = 1000 | 1 |
| **CAMH** | GE | 1.5T | 3 | Voxel size=2x2mm Thickness | 23 at b=1000 | 2 |
| **BWH_FE (CIDAR)** | GE Echospeed | 3T | 1 | 1.7x1.7x1.7 mm | 51 at b=900 | 8 |
| **BWH_chronic (CIDAR)** | GE Echospeed | 3T | 1 | 1.7x1.7x1.7 mm | 51 at b=900 | 8 |
| **Dublin** | Philips Achieva | 3T | 1 | 2x2x2.3mm | 15 at b=800 | 1 |
| **Galway** | Siemens Magnetom Symphony | 1.5T | 1 | 2.5x2.5x2.5 | 64 at b=1300 | 7 |
| **UPENN (12)** | Siemens Trio | 3T | 1 | 1.72x1.72x3mm | 12 at b=1000 | 1 |
| **UPENN (64)** | Siemens Trio | 3T | 1 | 1.875x1.875x2.0mm | 64 at b=1000 | 1 |
| **MPRC (Study1)** | Siemens Alegra | 3T | 1 | 1.7×1.7×4.0 | 12 at b=1000 | 8 |
| **MPRC (Study2)** | Siemens Trio | 3T | 1 | 1.8×1.8×3.0 | 30 at b=1000 | 3 |
| **MPRC (Study3)** | Siemens Trio | 3T | 1 | 1.7×1.7×3.0 | 64 at b=800 | 5 |
| **UMCU** | Philips Achieva | 1.5T | 2 | 2.5x2.5x2.5 | 32 at b = 1000 | 8 |
| **Roma_S_Lucia (Rome)** | Siemens Allegra | 3T | 3 | 1.8x1.8x1.8 mm3 | 30 at b=1000 | 2 |
| **CIAM_SouthAfrica** | Siemens Allegra | 3T | 2 - one AP, one PA | 1.8×1.8×4.0 mm. | 30 at b = 1000 and b = 0 | 2 |
| **Australia (ASRB) site 1** | Siemnes Avanto | 1.5 T | 1, AP, shimmed EPI | 2.4x 2.4x 2.4 mm | 64 at b =1000 | 1 |
| **Australia (ASRB) site 2** | Siemnes Avanto | 1.5 T | 1 AP, shimmed EPI | 2.4x 2.4x 2.4 mm | 64 dx, b =1000 | 1 |
| **Australia (ASRB) site 3** | Siemnes Avanto | 1.5 T | 1 AP, shimmed EPI | 2.4x 2.4x 2.4 mm | 64 dx, b =1000 | 1 |
| **Australia (ASRB) site 4** | Siemnes Avanto | 1.5 T | 1 AP, shimmed EPI | 2.4x 2.4x 2.4 mm | 64 dx, b =1000 | 1 |
| **Australia (ASRB) site 5** | Siemnes Avanto | 1.5 T | 1 AP, shimmed EPI | 2.4x 2.4x 2.4 mm | 64 dx, b =1000 | 1 |
| **FBIRN** | Siemens Trio | 3T | 1 | 2x2x2 mm | 30 at b=800 | 5 |
| **Huilongguan Siemens** | Siemens Trio | 3T | 1 AP, shimmed EPI | 1.8 x1.8 x3.0 mm | 64 dx, b =1000 mm/s2 | 1 |
| **Huilongguan GE** | GE | 3T | 1 AP, shimmed EPI | 1.0 x1.0 x3.0 mm | 32 dx, b =1000 mm/s2 | 3 |
| **Edinburgh** | Siemens Magnetom Verio | 3T | 1 | 2.2x2.5x2.5mm | 56 at b=1000 | 6 |

**Supplementary Table 1.** DTI acquisition protocols for each site

| **Dataset name** | **N cases/controls** | **Mean age (cases)** | **Mean age (controls)** | **std dev age (cases)** | **std dev age (controls)** | **Age range patients** | **Age range controls** | **M/F (cases)** | **M/F (controls)** |
| --- | --- | --- | --- | --- | --- | --- | --- | --- | --- |
| **ESO study (Prague)** | 77/59 | 31.1 | 28.9 | 7.7 | 7.1 | 19-53 | 19-48 | 34/43 | 28/31 |
| **Osaka** | 71/249 | 34 | 31.2 | 12.1 | 13.2 | 18-68 | 18-66 | 36/35 | 138/111 |
| **HUBIN** | 37/45 | 51.95 | 54.62 | 8.21 | 8.69 | 37-64 | 33-69 | 8/29 | 31/14 |
| **TOP** | 76/275 | 28.08 | 31.89 | 7.83 | 7.56 | 18-53 | 18-46 | 48/28 | 161/114 |
| **Brain and Mind Research Institute (SYD)** | 51/73 | 23.34 | 26.77 | 4.53 | 23.69 | 18-16 | 18-35 | 37/14 | 31/42 |
| **Olin(12 direction)** | 57/100 | 34.5 | 31 | 11.82 | 11.75 | 18-56 | 18-77 | 36/21 | 53/47 |
| **Olin(33 direction)** | 63/83 | 35.9 | 36 | 13.11 | 13.14 | 18-64 | 20-70 | 47/16 | 62/21 |
| **CAMH** | 113/134 | 41.77 | 42.43 | 16.4 | 19.2 | 18-77 | 18-86 | 70/43 | 66/68 |
| **BWH_FE (CIDAR)** | 18/18 | 36.762 | 36.502 | 13.891 | 12.681 | 18-53 | 18-57 | 3/15 | 8/10 |
| **BWH_chronic (CIDAR)** | 37/33 | 45.66 | 45.26 | 9 | 6.56 | 27-56 | 29-53 | 7/30 | 6/27 |
| **Dublin** | 49/140 | 43.5 | 32.24 | 10.9 | 11.58 | 22-61 | 19-64 | 35/14 | 68/72 |
| **Galway** | 44/88 | 34.09 | 35.93 | 10.4 | 10..97 | 19-58 | 18-57 | 33/11 | 55/33 |
| **Upenn (12)** | 15/14 | 35.53 | 29.35 | 8.8 | 6.23 | 21-50 | 21-42 | 10/5 | 8/6 |
| **Upenn (64)** | 44/37 | 39.2 | 32.7 | 10.02 | 9.6 | 22-60 | 19-58 | 24/20 | 18/19 |
| **MPRC (Study1)** | 59/48 | 38.71 | 36.98 | 12.25 | 13.13 | 21-62 | 19-61 | 49/10 | 33/15 |
| **MPRC (Study2)** | 82/76 | 36.53 | 42.92 | 12.29 | 12.44 | 18-63 | 18-64 | 58/24 | 31/45 |
| **MPRC (Study3)** | 103/168 | 35.79 | 38.29 | 12.7 | 15.15 | 18-59 | 18-77 | 72/31 | 74/94 |
| **UMCU** | 128/87 | 26.38 | 27.06 | 5.7 | 8 | 18-43 | 18-45 | 106/22 | 44/45 |
| **Roma_S_Lucia** | 84/148 | 39.261 | 48.282 | 11.588 | 15.919 | 18-66 | 19-78 | 56/28 | 76/72 |
| **CIAM_SouthAfrica** | 12/25 | 31.25 | 25.88 | 7.65 | 4.39 | 20-40 | 19-34 | 8/4 | 12/13 |
| **Australia (ASRB) site 1** | 121/33 | 38.67 | 40.15 | 10.98 | 14.05 | 20-65 | 18-63 | 89/32 | 16/17 |
| **Australia (ASRB) site 2** | 85/79 | 37.83 | 41.3 | 10.43 | 13.78 | 20-63 | 19-65 | 54/31 | 41/38 |
| **Australia (ASRB) site 3** | 17/18 | 41.7 | 43.94 | 8.72 | 13.64 | 27-58 | 19-62 | 12/5 | 9/9 |
| **Australia (ASRB) site 4** | 39/29 | 38.72 | 37 | 10.38 | 13.73 | 20-59 | 18-65 | 28/11 | 15/14 |
| **Australia (ASRB) site 5** | 64/38 | 40.04 | 40 | 10.41 | 14.89 | 20-64 | 18-62 | 42/22 | 18/20 |
| **FBIRN** | 143/146 | 38.93 | 37.05 | 11.78 | 10.91 | 18-62 | 19-60 | 109/34 | 104/42 |
| **Huilongang Siemens** | 154/36 | 29.02 | 31.75 | 5.62 | 6.51 | 18-40 | 19-40 | 84/70 | 20/16 |
| **Huilongang GE** | 90/44 | 24.25 | 24.93 | 6.11 | 5.64 | 18-43 | 18-37 | 48/42 | 25/19 |
| **Edinburgh** | 28/35 | 38.04 | 37.8 | 9.63 | 14.8 | 23-57 | 20-67 | 17/11 | 18/17 |

**Supplementary Table 2:** ENIGMA-Schizophrenia DTI site demographics

| **Site** | **PANSS Pos** | **PANSS Neg** | **PANSS Total** | **SANS Total** | **SAPS Total** | **Mean age at onset** | **Mean duration of illness** | **Mean CPZ (Woods, 2005)** | **Number of patients on Typical/Atypical/Both/Neither** |
| --- | --- | --- | --- | --- | --- | --- | --- | --- | --- |
| **ESO study (Prague)** | 19.8 | 16.2 | 74.3 | n/a | n/a | n/a | 5.5 months | 306.9 | 1/65/1/4 |
| **Osaka** | 19.2 | 20.6 | 84.6 | n/a | n/a | 22.8 | 11.2 | 731.3 | 3/62/8/7 |
| **HUBIN** | n/a | n/a | n/a | 28.4 +/- 14.2 | 9 +/- 7.9 | 23.6 +/- 4.22 | 28.35 +/- 8.51 | 348.58 +/- 296.09 | 11/15/8/3 |
| **TOP** | 13.83 +/- 4.88 | 14.54 +/- 4.96 | 57.59 +/- 13.29 | n/a | n/a | 21.27 +/- 6.28 | 6.85 +/- 6.55 | 340.75 +/- 310.9 | 2/50/2/2 |
| **Brain and Mind Research Institute (SYD)** | NA | NA | NA | NA | NA | 18.16y | 5.48y | 339.6 | 0/46/0/18 |
| **Olin (12 direction)** | NA | NA | NA | NA | NA | 21.9(6.1) | 9.3(8.0) | NA | NA |
| **Olin (33 direction)** | NA | NA | NA | NA | NA | NA | NA | NA | Na |
| **CAMH** | 13.8(6.0) | 13.2(5.7) | 51.8(15.23) | NA | NA | 24.9(9.2) | 16.8(15.1) year | 272.6(291.4) | 7/78/8/20 |
| **BWH_FE (CIDAR)** | na | na | 86.353 +/-30.723 | 21.46 +/-12.69 | 22.98 +/-22.25 | 22.17+/-4.93 | 13.09 +/-12.37 year | 416.784 +/- 400.899 | NA |
| **BWH_chronic (CIDAR)** | NA | NA | NA | 25.05 | 26.97 | 23.52 | 22.22 | 430.22 | NA |
| **Dublin** | NA | NA | NA | 3.46 | 2.38 | 23 | 19.56 | 339 |  |
| **Galway** | 13.76 | 22.92 | 22.92 | NA | NA | 23.72 | 10.48 | 482.83 | NA |
| **Upenn (12)** | NA | NA | NA | 13.4 | 14.26 | 20.07 | 15.21 | 379.166 | 0/4/0/0 |
| **Upenn (64)** | NA | NA | NA | 21.29 | 13.77 | 21.59 | 18.07 | 18.07 | 4/4/2/0 |
| **MPRC (Study1)** | NA | NA | NA | NA | NA | NA | NA | NA | NA |
| **MPRC (Study2)** | NA | NA | NA | NA | NA | NA | NA | NA | NA |
| **MPRC (Study3)** | NA | NA | NA | NA | NA | NA | NA | NA | NA |
| **UMCU** | 15.43 | 15.64 | 62.88 | n/a | n/a | 21.9 | 3.67 | NA | 11/102/2/143 |
| **Roma_S_Lucia** | 23.233 | 20.791 | 92.651 | 9.702 | 9.06 | 25.261 | 14y | 416.942 | 15/44/23/6 |
| **CIAM_SouthAfrica** | 12.6(4.9) | 15.2(6.4) | 54.2(17.5) | n/a | n/a | 21.9(6.1) | 9.3(8.0) | NA | 4/6/1/1 |
| **Australia (ASRB) site 1** | NA | NA | NA | NA | NA | 22.9 | 15.7 | NA | NA |
| **Australia (ASRB) site 2** | NA | NA | NA | NA | NA | 23.3 | 14.5 | NA | NA |
| **Australia (ASRB) site 3** | NA | NA | NA | NA | NA | 23.4 | 18.2 | NA | NA |
| **Australia (ASRB) site 4** | NA | NA | NA | NA | NA | 23 | 15.8 | NA | NA |
| **Australia (ASRB) site 5** | NA | NA | NA | NA | NA | 22.8 | 17.3 | NA | NA |
| **FBIRN** | 15.17 | 14.63 | 14.63 | 20.52 | 15.79 | 21.96 | 17.05 | 380.65 | 16/106/6/0 |
| **Huilongguan Siemens** | NA | NA | NA | NA | NA | NA | NA | NA | 55/0/11/0 |
| **Huilongguan** | NA | NA | NA | NA | NA | NA | NA | 301.28 | NA |
| **Edinburgh** | 12.29 | 13.07 | 13.07 | 23.64 | NA | 24.25 | 13.58 | 434.97 | 0/23/0/3 |

**Supplementary Table 3: ENIGMA-Schizophrenia DTI clinical information**

| ROI | Cohen’s d | SE | P-value | CI lowerbound | CI upperbound | i^2^ |
| --- | --- | --- | --- | --- | --- | --- |
| ACR | -0.40 | 0.045 | 9.18x10^19^ | -0.491 | -0.313 | 43.83 |
| ACR_L | -0.36 | 0.045 | 1.27x10^-15^ | -0.451 | -0.274 | 43.80 |
| ACR_R | -0.39 | 0.046 | 4.28x10^-18^ | -0.484 | -0.306 | 44.19 |
| ALIC | -0.37 | 0.046 | 2.27x10^-15^ | -0.459 | -0.277 | 46.30 |
| ALIC_L | -0.35 | 0.045 | 8.14x10^-15^ | -0.442 | -0.264 | 44.01 |
| ALIC_R | -0.34 | 0.049 | 5.69x10^-12^ | -0.435 | -0.242 | 52.10 |
| AverageFA | -0.42 | 0.042 | 4.50x10^-24^ | -0.506 | -0.342 | 34.65 |
| BCC | -0.39 | 0.045 | 2.51x10^-18^ | -0.483 | -0.306 | 43.24 |
| CC | -0.40 | 0.045 | 8.36x10^-19^ | -0.485 | -0.309 | 42.51 |
| CGC | -0.27 | 0.045 | 2.95x10^-9^ | -0.354 | -0.178 | 42.87 |
| CGC_L | -0.23 | 0.044 | 1.57x10^-7^ | -0.318 | -0.145 | 41.50 |
| CGC_R | -0.28 | 0.045 | 6.38x10^-10^ | -0.367 | -0.190 | 43.57 |
| CGH | -0.11 | 0.041 | 0.0082 | -0.190 | -0.028 | 33.73 |
| CGH_L | -0.09 | 0.043 | 0.034 | -0.175 | -0.007 | 38.60 |
| CGH_R | -0.11 | 0.038 | 0.0048 | -0.183 | -0.033 | 24.40 |
| CR | -0.33 | 0.040 | 3.16x10^-17^ | -0.412 | -0.257 | 27.89 |
| CR_L | -0.32 | 0.040 | 1.96x10^-15^ | -0.393 | -0.238 | 28.37 |
| CR_R | -0.33 | 0.041 | 6.74x10^-16^ | -0.406 | -0.248 | 30.87 |
| CST | -0.04 | 0.038 | 0.24 | -0.120 | 0.030 | 24.20 |
| CST_L | 0.00 | 0.035 | 0.99 | -0.067 | 0.068 | 9.75 |
| CST_R | -0.09 | 0.042 | 0.033 | -0.170 | -0.007 | 29.99 |
| EC | -0.21 | 0.040 | 1.41x10^-7^ | -0.292 | -0.133 | 31.01 |
| EC_L | -0.20 | 0.043 | 2.67x10^-6^ | -0.287 | -0.118 | 38.81 |
| EC_R | -0.19 | 0.042 | 3.09x10^-6^ | -0.277 | -0.113 | 35.13 |
| FX | -0.31 | 0.045 | 6.76x10^-12^ | -0.397 | -0.221 | 43.25 |
| FX_ST_L | -0.29 | 0.041 | 2.27x10^-12^ | -0.372 | -0.210 | 33.90 |
| FX_ST_R | -0.28 | 0.044 | 8.40x10^-11^ | -0.371 | -0.199 | 40.38 |
| FXST | -0.32 | 0.042 | 8.28x10^-14^ | -0.400 | -0.233 | 36.39 |
| GCC | -0.37 | 0.042 | 1.24x10^-18^ | -0.453 | -0.288 | 35.22 |
| IC | -0.18 | 0.042 | 1.79x10^-5^ | -0.264 | -0.098 | 36.25 |
| IC_L | -0.18 | 0.041 | 1.48x10^-5^ | -0.261 | -0.098 | 34.11 |
| IC_R | -0.17 | 0.045 | 0.00025 | -0.255 | -0.077 | 44.56 |
| IFO | -0.11 | 0.038 | 0.00355 | -0.185 | -0.036 | 22.70 |
| IFO_L | -0.07 | 0.042 | 0.1 | -0.152 | 0.014 | 36.50 |
| IFO_R | -0.12 | 0.036 | 0.00095 | -0.189 | -0.048 | 15.48 |
| PCR | -0.25 | 0.036 | 2.40x10^-12^ | -0.324 | -0.183 | 15.76 |
| PCR_L | -0.27 | 0.04 | 3.47x10^-13^ | -0.340 | -0.196 | 18.42 |
| PCR_R | -0.21 | 0.04 | 2.50x10^-9^ | -0.277 | -0.140 | 11.42 |
| PLIC | 0.04 | 0.05 | 0.37 | -0.049 | 0.132 | 46.20 |
| PLIC_L | 0.05 | 0.05 | 0.3 | -0.041 | 0.133 | 42.67 |
| PLIC_R | 0.03 | 0.05 | 0.53 | -0.064 | 0.124 | 50.41 |
| PTR | -0.31 | 0.04 | 1.20x10^-18^ | -0.377 | -0.239 | 11.78 |
| PTR_L | -0.27 | 0.04 | 1.67x10^-15^ | -0.341 | -0.206 | 8.55 |
| PTR_R | -0.28 | 0.04 | 3.70x10^-15^ | -0.356 | -0.214 | 16.06 |
| RLIC | -0.13 | 0.04 | 0.0021 | -0.207 | -0.046 | 33.10 |
| RLIC_L | -0.14 | 0.04 | 9.15x10^-5^ | -0.216 | -0.072 | 18.80 |
| RLIC_R | -0.09 | 0.05 | 0.049 | -0.181 | 0.000 | 46.34 |
| SCC | -0.22 | 0.05 | 4.39x10^-6^ | -0.317 | -0.128 | 50.94 |
| SCR | -0.15 | 0.03 | 6.91x10^-6^ | -0.220 | -0.087 | 7.84 |
| SCR_L | -0.13 | 0.03 | 0.00013 | -0.197 | -0.064 | 7.56 |
| SCR_R | -0.16 | 0.04 | 1.81x10^-5^ | -0.233 | -0.087 | 20.65 |
| SFO | -0.29 | 0.06 | 3.96x10^-8^ | -0.399 | -0.189 | 59.68 |
| SFO_L | -0.27 | 0.05 | 9.49x10^-10^ | -0.363 | -0.187 | 43.16 |
| SFO_R | -0.26 | 0.06 | 1.61x10^-5^ | -0.374 | -0.140 | 67.57 |
| SLF | -0.22 | 0.04 | 5.61x10^-8^ | -0.294 | -0.138 | 35.17 |
| SLF_L | -0.22 | 0.04 | 3.33x10^-8^ | -0.297 | -0.141 | 36.18 |
| SLF_R | -0.19 | 0.04 | 1.44x10^-6^ | -0.266 | -0.112 | 31.05 |
| SS | -0.30 | 0.05 | 4.91x10^-14^ | -0.383 | -0.225 | 44.32 |
| SS_L | -0.31 | 0.05 | 1.23x10^-12^ | -0.401 | -0.228 | 52.65 |
| SS_R | -0.24 | 0.04 | 1.90x10^-11^ | -0.310 | -0.170 | 28.06 |
| UNC | -0.16 | 0.03 | 8.78x10^-7^ | -0.229 | -0.098 | 5.02 |
| UNC_L | -0.14 | 0.03 | 1.10x10^-5^ | -0.208 | -0.080 | 1.15 |
| UNC_R | -0.16 | 0.03 | 4.25x10^-6^ | -0.221 | -0.089 | 7.00 |

**Supplementary Table 4.** Lateralized results for case/control FA differences

| **ROI** | **Meta b SANS** | **Meta b SAPS** | **SE SANS** | **SE SAPS** | **P-value SANS** | **P value SAPS** |
| --- | --- | --- | --- | --- | --- | --- |
| **ACR** | -0.00021 | -0.00037 | 0.00017 | 0.00031 | 0.21 | 0.231 |
| **ALIC** | -0.00024 | -0.00039 | 0.00010 | 0.00022 | 0.01 | 0.073 |
| **Average FA** | -0.00018 | -0.00018 | 0.00012 | 0.00007 | 0.12 | 0.009 |
| **BCC** | -0.00019 | -0.00032 | 0.00019 | 0.00019 | 0.33 | 0.082 |
| **CC** | -0.00027 | -0.00032 | 0.00011 | 0.00011 | 0.01 | 0.004 |
| **CGC** | -0.00037 | -0.00026 | 0.00017 | 0.00013 | 0.03 | 0.057 |
| **CGH** | -0.00024 | -0.00007 | 0.00018 | 0.00061 | 0.18 | 0.907 |
| **CR** | -0.00017 | -0.00019 | 0.00012 | 0.00015 | 0.17 | 0.186 |
| **CST** | 0.00005 | -0.00022 | 0.00037 | 0.00015 | 0.89 | 0.160 |
| **EC** | -0.00008 | -0.00004 | 0.00023 | 0.00009 | 0.72 | 0.648 |
| **FX** | -0.00007 | -0.00027 | 0.00026 | 0.00026 | 0.80 | 0.302 |
| **FXST** | -0.00009 | -0.00016 | 0.00028 | 0.00012 | 0.75 | 0.186 |
| **GCC** | -0.00033 | -0.00036 | 0.00012 | 0.00013 | 0.01 | 0.004 |
| **IC** | -0.00019 | -0.00019 | 0.00008 | 0.00008 | 0.02 | 0.013 |
| **IFO** | 0.00017 | -0.00009 | 0.00015 | 0.00014 | 0.27 | 0.519 |
| **PCR** | -0.00014 | -0.00016 | 0.00014 | 0.00015 | 0.31 | 0.302 |
| **PLIC** | -0.00012 | -0.00009 | 0.00010 | 0.00011 | 0.25 | 0.441 |
| **PTR** | -0.00037 | -0.00021 | 0.00012 | 0.00015 | 0.001 | 0.157 |
| **RLIC** | -0.00014 | -0.00021 | 0.00023 | 0.00009 | 0.54 | 0.026 |
| **SCC** | -0.00018 | -0.00030 | 0.00011 | 0.00012 | 0.11 | 0.016 |
| **SCR** | -0.00006 | -0.00003 | 0.00012 | 0.00009 | 0.61 | 0.740 |
| **SFO** | -0.00001 | -0.00036 | 0.00031 | 0.00037 | 0.97 | 0.329 |
| **SLF** | -0.00025 | -0.00015 | 0.00015 | 0.00010 | 0.10 | 0.105 |
| **SS** | -0.00018 | -0.00010 | 0.00022 | 0.00010 | 0.41 | 0.331 |
| **UNC** | -0.00042 | -0.00030 | 0.00032 | 0.00046 | 0.18 | 0.517 |

**Supplementary Table 5:** Partial correlation betas (meta b), and p-values for symptom severity as measured by SAPS and SANS scores

| **ROI** | **Meta b PANSSTOT** | **Meta b PANSSNEG** | **SE PANSSTOT** | **SE PANSSNEG** | **P-value PANSSTOT** | **P value PANSSNEG** |
| --- | --- | --- | --- | --- | --- | --- |
| **ACR** | -0.00011 | -0.00026 | 0.00007 | 0.00024 | 0.129 | 0.282 |
| **ALIC** | -0.00017 | -0.00059 | 0.00006 | 0.00018 | 0.003 | 0.0014 |
| **Average FA** | -0.00013 | -0.0003 | 0.00007 | 0.00021 | 0.08 | 0.152 |
| **BCC** | -0.0002 | -0.00073 | 0.00011 | 0.00042 | 0.067 | 0.081 |
| **CC** | -0.00014 | -0.00047 | 0.00007 | 0.00026 | 0.04 | 0.064 |
| **CGC** | -0.00019 | -0.00026 | 0.00012 | 0.00027 | 0.107 | 0.335 |
| **CGH** | -0.00011 | -0.00023 | 0.00011 | 0.0004 | 0.318 | 0.56 |
| **CR** | -0.00015 | -0.00029 | 0.00007 | 0.00021 | 0.035 | 0.16 |
| **CST** | -0.00011 | -0.00059 | 0.00011 | 0.00032 | 0.316 | 0.061 |
| **EC** | -0.00008 | -0.0002 | 0.00006 | 0.00016 | 0.136 | 0.197 |
| **FX** | -0.0001 | -0.00043 | 0.00018 | 0.0004 | 0.594 | 0.274 |
| **FXST** | -0.00008 | -0.00007 | 0.0001 | 0.00026 | 0.436 | 0.781 |
| **GCC** | -0.00009 | -0.00026 | 0.00007 | 0.00024 | 0.191 | 0.283 |
| **IC** | -0.00009 | -0.00026 | 0.00005 | 0.00014 | 0.048 | 0.077 |
| **IFO** | -0.00002 | -0.00031 | 0.00009 | 0.00015 | 0.821 | 0.042 |
| **PCR** | -0.0002 | -0.0002 | 0.00008 | 0.00015 | 0.009 | 0.169 |
| **PLIC** | -0.00006 | 0.00019 | 0.00006 | 0.00027 | 0.279 | 0.489 |
| **PTR** | -0.00014 | -0.00047 | 0.00007 | 0.00023 | 0.06 | 0.044 |
| **RLIC** | -0.00006 | -0.00026 | 0.00006 | 0.00017 | 0.284 | 0.128 |
| **SCC** | -0.00014 | -0.00059 | 0.00007 | 0.00021 | 0.041 | 0.006 |
| **SCR** | -0.00018 | -0.00005 | 0.00008 | 0.00018 | 0.029 | 0.8 |
| **SFO** | -0.00004 | -0.00035 | 0.00009 | 0.00019 | 0.629 | 0.072 |
| **SLF** | -0.00016 | -0.00041 | 0.0001 | 0.00027 | 0.098 | 0.135 |
| **SS** | -0.00011 | -0.00019 | 0.00007 | 0.00042 | 0.101 | 0.65 |
| **UNC** | -0.00022 | -0.00027 | 0.00013 | 0.00019 | 0.092 | 0.166 |

**Supplementary Table 6:** Partial correlation betas (meta b), and p-values for symptom severity as measured by PANSS total and PANSS negative scores

| **ROI** | **Cohen’s d** | **N controls** | **N patients** | **N80** |
| --- | --- | --- | --- | --- |
| **AverageFA** | -0.42 | 2359 | 1963 | 90 |
| **ACR** | -0.40 | 2359 | 1963 | 100 |
| **CC** | -0.40 | 2359 | 1963 | 100 |
| **BCC** | -0.39 | 2359 | 1963 | 105 |
| **GCC** | -0.37 | 2359 | 1963 | 116 |
| **ALIC** | -0.37 | 2359 | 1963 | 116 |
| **CR** | -0.33 | 2359 | 1963 | 146 |
| **FXST** | -0.32 | 2359 | 1963 | 155 |
| **FX** | -0.31 | 2359 | 1963 | 165 |
| **PTR** | -0.31 | 2359 | 1963 | 165 |
| **SS** | -0.30 | 2359 | 1963 | 176 |
| **SFO** | -0.29 | 2359 | 1963 | 188 |
| **CGC** | -0.27 | 2359 | 1963 | 217 |
| **PCR** | -0.25 | 2359 | 1963 | 253 |
| **SCC** | -0.22 | 2359 | 1963 | 326 |
| **SLF** | -0.22 | 2359 | 1963 | 326 |
| **EC** | -0.21 | 2359 | 1963 | 357 |
| **IC** | -0.18 | 2359 | 1963 | 486 |
| **UNC** | -0.16 | 2359 | 1963 | 615 |
| **SCR** | -0.15 | 2359 | 1963 | 699 |
| **RLIC** | -0.13 | 2359 | 1963 | 930 |
| **IFO** | -0.11 | 2359 | 1963 | 1299 |
| **CGH** | -0.10 | 2359 | 1963 | 1571 |
| **CST** | -0.04 | 2359 | 1963 | 9813 |
| **PLIC** | 0.05 | 2359 | 1963 | 6281 |

**Supplementary Table 7.** N80: The total number of samples required, per group, to achieve 80% power to detect group differences using a t-test at the threshold of *p*<0.05 (two-tailed).

| ADNI 3-month | N=19 (baseline, 3 months, 6 months) | | |
| --- | --- | --- | --- |
| ROI (FA) | **ICC** | **CI lowerbound** | **CI upperbound** |
| ACR | 0.989 | 0.976 | 0.995 |
| ALIC | 0.981 | 0.96 | 0.992 |
| Average_FA | 0.986 | 0.967 | 0.995 |
| BCC | 0.977 | 0.948 | 0.99 |
| CC | 0.979 | 0.95 | 0.991 |
| CGC | 0.926 | 0.843 | 0.969 |
| CGH | 0.941 | 0.871 | 0.976 |
| CR | 0.992 | 0.983 | 0.997 |
| CST | 0.947 | 0.886 | 0.978 |
| EC | 0.982 | 0.963 | 0.993 |
| FX | 0.954 | 0.902 | 0.981 |
| FXST | 0.962 | 0.919 | 0.984 |
| GCC | 0.974 | 0.945 | 0.989 |
| IC | 0.983 | 0.965 | 0.993 |
| IFO | 0.951 | 0.893 | 0.979 |
| PCR | 0.993 | 0.985 | 0.997 |
| PLIC | 0.95 | 0.893 | 0.979 |
| PTR | 0.989 | 0.976 | 0.995 |
| RLIC | 0.987 | 0.97 | 0.995 |
| SCC | 0.959 | 0.909 | 0.983 |
| SCR | 0.991 | 0.98 | 0.996 |
| SFO | 0.972 | 0.94 | 0.988 |
| SLF | 0.985 | 0.966 | 0.994 |
| SS | 0.987 | 0.972 | 0.994 |
| UNC | 0.988 | 0.973 | 0.995 |

Supplementary Table 8: Intraclass correlation coefficients of FA measures for 19 healthy individuals scanned at baseline, 3 months and 6 months follow-up.

| ADNI 6-month | N=19 (baseline, 3 months, 6 months) | |  |
| --- | --- | --- | --- |
| ROI (AD) | **ICC** | **CI lowerbound** | **CI upperbound** |
| ACR | 0.957 | 0.908 | 0.982 |
| ALIC | 0.987 | 0.972 | 0.995 |
| Average_FA | 0.99 | 0.979 | 0.996 |
| BCC | 0.976 | 0.948 | 0.99 |
| CC | 0.978 | 0.954 | 0.991 |
| CGC | 0.956 | 0.906 | 0.982 |
| CGH | 0.952 | 0.897 | 0.98 |
| CR | 0.988 | 0.975 | 0.995 |
| CST | 0.938 | 0.868 | 0.974 |
| EC | 0.992 | 0.982 | 0.997 |
| FX | 0.978 | 0.953 | 0.991 |
| FXST | 0.956 | 0.907 | 0.982 |
| GCC | 0.969 | 0.934 | 0.987 |
| IC | 0.984 | 0.965 | 0.993 |
| IFO | 0.982 | 0.962 | 0.993 |
| PCR | 0.996 | 0.991 | 0.998 |
| PLIC | 0.97 | 0.936 | 0.988 |
| PTR | 0.987 | 0.973 | 0.995 |
| RLIC | 0.978 | 0.953 | 0.991 |
| SCC | 0.979 | 0.955 | 0.991 |
| SCR | 0.993 | 0.984 | 0.997 |
| SFO | 0.989 | 0.977 | 0.996 |
| SLF | 0.991 | 0.982 | 0.996 |
| SS | 0.979 | 0.956 | 0.991 |
| UNC | 0.991 | 0.98 | 0.996 |

Supplementary Table 11: Intraclass correlation coefficients of AD (axial diffusivity) measures for 19 healthy individuals scanned at baseline, 3 months and 6 months follow-up.

| ADNI 6-month v 3-month | N=19 (baseline, 3 months, 6 months) | |  |
| --- | --- | --- | --- |
| ROI (RD) | **ICC** | **CI lowerbound** | **CI upperbound** |
| ACR | 0.986 | 0.97 | 0.994 |
| ALIC | 0.993 | 0.984 | 0.997 |
| Average_FA | 0.992 | 0.983 | 0.997 |
| BCC | 0.993 | 0.984 | 0.997 |
| CC | 0.99 | 0.974 | 0.996 |
| CGC | 0.981 | 0.958 | 0.992 |
| CGH | 0.968 | 0.932 | 0.987 |
| CR | 0.993 | 0.985 | 0.997 |
| CST | 0.892 | 0.766 | 0.955 |
| EC | 0.994 | 0.987 | 0.997 |
| FX | 0.983 | 0.963 | 0.993 |
| FXST | 0.971 | 0.938 | 0.988 |
| GCC | 0.983 | 0.96 | 0.993 |
| IC | 0.991 | 0.981 | 0.996 |
| IFO | 0.954 | 0.902 | 0.981 |
| PCR | 0.997 | 0.993 | 0.999 |
| PLIC | 0.965 | 0.925 | 0.985 |
| PTR | 0.991 | 0.981 | 0.996 |
| RLIC | 0.99 | 0.979 | 0.996 |
| SCC | 0.973 | 0.94 | 0.989 |
| SCR | 0.992 | 0.982 | 0.997 |
| SFO | 0.988 | 0.975 | 0.995 |
| SLF | 0.994 | 0.987 | 0.997 |
| SS | 0.992 | 0.983 | 0.997 |
| UNC | 0.993 | 0.985 | 0.997 |

Supplementary Table 12: Intraclass correlation coefficients of RD (radial diffusivity) measures for 19 healthy individuals scanned at baseline, 3 months and 6 months follow-up.

| ADNI 6-month v 3-month | N=19 (baseline, 3 months, 6 months) | | |
| --- | --- | --- | --- |
| ROI (MD) | **ICC** | **CI lowerbound** | **CI upperbound** |
| ACR | 0.96 | 0.914 | 0.983 |
| ALIC | 0.991 | 0.982 | 0.996 |
| Average_FA | 0.966 | 0.928 | 0.986 |
| BCC | 0.957 | 0.908 | 0.982 |
| CC | 0.944 | 0.881 | 0.977 |
| CGC | 0.957 | 0.908 | 0.982 |
| CGH | 0.945 | 0.884 | 0.977 |
| CR | 0.983 | 0.964 | 0.993 |
| CST | 0.854 | 0.687 | 0.939 |
| EC | 0.99 | 0.979 | 0.996 |
| FX | 0.757 | 0.475 | 0.899 |
| FXST | 0.757 | 0.475 | 0.899 |
| GCC | 0.963 | 0.921 | 0.985 |
| IC | 0.987 | 0.973 | 0.995 |
| IFO | 0.964 | 0.923 | 0.985 |
| PCR | 0.996 | 0.991 | 0.998 |
| PLIC | 0.969 | 0.934 | 0.987 |
| PTR | 0.974 | 0.945 | 0.989 |
| RLIC | 0.979 | 0.956 | 0.991 |
| SCC | 0.913 | 0.815 | 0.964 |
| SCR | 0.712 | 0.138 | 0.899 |
| SFO | 0.983 | 0.963 | 0.993 |
| SLF | 0.983 | 0.963 | 0.993 |
| SS | 0.979 | 0.955 | 0.991 |
| UNC | 0.991 | 0.98 | 0.996 |

Supplementary Table 13: Intraclass correlation coefficients of MD (mean diffusivity) measures for 19 healthy individuals scanned at baseline, 3 months and 6 months follow-up.

| ADNI 3-month | N=19 (baseline, 3 months) | |  |
| --- | --- | --- | --- |
| ROI (FA) | **ICC** | **CI lowerbound** | **CI upperbound** |
| ACR | 0.981 | 0.95 | 0.992 |
| ALIC | 0.978 | 0.944 | 0.991 |
| Average_FA | 0.978 | 0.941 | 0.992 |
| BCC | 0.967 | 0.906 | 0.988 |
| CC | 0.964 | 0.894 | 0.987 |
| CGC | 0.905 | 0.759 | 0.963 |
| CGH | 0.931 | 0.797 | 0.975 |
| CR | 0.987 | 0.962 | 0.995 |
| CST | 0.88 | 0.688 | 0.954 |
| EC | 0.968 | 0.918 | 0.988 |
| FX | 0.925 | 0.805 | 0.971 |
| FXST | 0.937 | 0.836 | 0.976 |
| GCC | 0.952 | 0.876 | 0.981 |
| IC | 0.98 | 0.949 | 0.992 |
| IFO | 0.948 | 0.864 | 0.98 |
| PCR | 0.988 | 0.963 | 0.995 |
| PLIC | 0.951 | 0.873 | 0.981 |
| PTR | 0.978 | 0.943 | 0.991 |
| RLIC | 0.978 | 0.944 | 0.991 |
| SCC | 0.913 | 0.763 | 0.967 |
| SCR | 0.982 | 0.954 | 0.993 |
| SFO | 0.978 | 0.943 | 0.991 |
| SLF | 0.975 | 0.923 | 0.991 |
| SS | 0.981 | 0.951 | 0.993 |
| UNC | 0.974 | 0.914 | 0.991 |

Supplementary Table 14: Intraclass correlation coefficients of FA measures for 19 healthy individuals scanned at baseline and 3 months.

| ADNI 3-month | N=19 (baseline, 3 months) | |  |
| --- | --- | --- | --- |
| ROI (AD) | **ICC** | **CI-lowerbound** | **CI-upperbound** |
| ACR | 0.954 | 0.88 | 0.982 |
| ALIC | 0.978 | 0.944 | 0.982 |
| Average_FA | 0.987 | 0.966 | 0.995 |
| BCC | 0.96 | 0.898 | 0.985 |
| CC | 0.972 | 0.927 | 0.989 |
| CGC | 0.902 | 0.749 | 0.962 |
| CGH | 0.932 | 0.803 | 0.975 |
| CR | 0.99 | 0.975 | 0.996 |
| CST | 0.891 | 0.719 | 0.958 |
| EC | 0.99 | 0.974 | 0.996 |
| FX | 0.972 | 0.921 | 0.99 |
| FXST | 0.934 | 0.831 | 0.974 |
| GCC | 0.964 | 0.909 | 0.986 |
| IC | 0.973 | 0.93 | 0.99 |
| IFO | 0.973 | 0.93 | 0.99 |
| PCR | 0.995 | 0.986 | 0.998 |
| PLIC | 0.95 | 0.872 | 0.981 |
| PTR | 0.982 | 0.954 | 0.993 |
| RLIC | 0.965 | 0.91 | 0.986 |
| SCC | 0.972 | 0.929 | 0.989 |
| SCR | 0.993 | 0.982 | 0.997 |
| SFO | 0.978 | 0.943 | 0.992 |
| SLF | 0.985 | 0.962 | 0.994 |
| SS | 0.972 | 0.929 | 0.989 |
| UNC | 0.985 | 0.962 | 0.994 |

Supplementary Table 15: Intraclass correlation coefficients of AD (axial diffusivity) measures for 19 healthy individuals scanned at baseline and 3 months.

| ADNI 3-month | N=19 (baseline, 3 months) | |  |
| --- | --- | --- | --- |
| ROI (MD) | **ICC** | **CI lowerbound** | **CI upperbound** |
| ACR | 0.938 | 0.839 | 0.976 |
| ALIC | 0.984 | 0.959 | 0.994 |
| Average_FA | 0.947 | 0.861 | 0.979 |
| BCC | 0.915 | 0.779 | 0.968 |
| CC | 0.904 | 0.75 | 0.963 |
| CGC | 0.93 | 0.817 | 0.973 |
| CGH | 0.927 | 0.815 | 0.972 |
| CR | 0.977 | 0.94 | 0.991 |
| CST | 0.797 | 0.479 | 0.922 |
| EC | 0.986 | 0.965 | 0.995 |
| FX | 0.419 | -0.589 | 0.781 |
| FXST | 0.958 | 0.892 | 0.984 |
| GCC | 0.943 | 0.854 | 0.978 |
| IC | 0.984 | 0.958 | 0.994 |
| IFO | 0.96 | 0.896 | 0.984 |
| PCR | 0.997 | 0.993 | 0.999 |
| PLIC | 0.964 | 0.908 | 0.986 |
| PTR | 0.962 | 0.901 | 0.985 |
| RLIC | 0.964 | 0.909 | 0.986 |
| SCC | 0.893 | 0.721 | 0.959 |
| SCR | 0.973 | 0.93 | 0.99 |
| SFO | 0.969 | 0.919 | 0.988 |
| SLF | 0.971 | 0.925 | 0.989 |
| SS | 0.968 | 0.918 | 0.988 |
| UNC | 0.982 | 0.955 | 0.993 |

Supplementary Table 16: Intraclass correlation coefficients of MD (mean diffusivity) measures for 19 healthy individuals scanned at baseline and 3 months.

| ADNI 3-month | N=19 (baseline, 3 months) | |  |
| --- | --- | --- | --- |
| ROI (RD) | **ICC** | **CI lowerbound** | **CI upperbound** |
| ACR | 0.981 | 0.952 | 0.993 |
| ALIC | 0.986 | 0.964 | 0.994 |
| Average_FA | 0.988 | 0.97 | 0.995 |
| BCC | 0.99 | 0.973 | 0.996 |
| CC | 0.983 | 0.95 | 0.994 |
| CGC | 0.971 | 0.927 | 0.989 |
| CGH | 0.962 | 0.902 | 0.985 |
| CR | 0.989 | 0.97 | 0.996 |
| CST | 0.811 | 0.503 | 0.927 |
| EC | 0.991 | 0.978 | 0.997 |
| FX | 0.976 | 0.939 | 0.991 |
| FXST | 0.966 | 0.913 | 0.987 |
| GCC | 0.975 | 0.936 | 0.99 |
| IC | 0.988 | 0.97 | 0.995 |
| IFO | 0.955 | 0.884 | 0.983 |
| PCR | 0.995 | 0.986 | 0.998 |
| PLIC | 0.962 | 0.903 | 0.985 |
| PTR | 0.982 | 0.954 | 0.993 |
| RLIC | 0.983 | 0.956 | 0.994 |
| SCC | 0.94 | 0.838 | 0.977 |
| SCR | 0.983 | 0.956 | 0.994 |
| SFO | 0.98 | 0.949 | 0.992 |
| SLF | 0.988 | 0.969 | 0.995 |
| SS | 0.99 | 0.973 | 0.996 |
| UNC | 0.985 | 0.958 | 0.994 |

Supplementary Table 17: Intraclass correlation coefficients of RD (radial diffusivity) measures for 19 healthy individuals scanned at baseline and 3 months.

| ADNI 6-month | N=19 (baseline, 6 months) | |  |
| --- | --- | --- | --- |
| ROI (FA) | **ICC** | **CI lowerbound** | **CI upperbound** |
| ACR | 0.986 | 0.95 | 0.995 |
| ALIC | 0.976 | 0.934 | 0.991 |
| Average_FA | 0.977 | 0.855 | 0.993 |
| BCC | 0.964 | 0.878 | 0.988 |
| CC | 0.969 | 0.816 | 0.991 |
| CGC | 0.85 | 0.618 | 0.942 |
| CGH | 0.923 | 0.704 | 0.974 |
| CR | 0.988 | 0.964 | 0.996 |
| CST | 0.949 | 0.871 | 0.98 |
| EC | 0.968 | 0.919 | 0.988 |
| FX | 0.923 | 0.805 | 0.97 |
| FXST | 0.932 | 0.828 | 0.974 |
| GCC | 0.966 | 0.898 | 0.988 |
| IC | 0.975 | 0.937 | 0.991 |
| IFO | 0.934 | 0.816 | 0.975 |
| PCR | 0.99 | 0.971 | 0.996 |
| PLIC | 0.91 | 0.766 | 0.965 |
| PTR | 0.985 | 0.959 | 0.994 |
| RLIC | 0.984 | 0.885 | 0.995 |
| SCC | 0.935 | 0.787 | 0.977 |
| SCR | 0.988 | 0.971 | 0.996 |
| SFO | 0.947 | 0.864 | 0.98 |
| SLF | 0.973 | 0.918 | 0.99 |
| SS | 0.98 | 0.946 | 0.992 |
| UNC | 0.98 | 0.948 | 0.992 |

Supplementary Table 18: Intraclass correlation coefficients of FA measures for 19 healthy individuals scanned at baseline and 6 months.

| ADNI 3-month | N=19 (baseline, 6 months) | |  |
| --- | --- | --- | --- |
| ROI (AD) | **ICC** | **CI-lowerbound** | **CI-upperbound** |
| ACR | 0.91 | 0.766 | 0.965 |
| ALIC | 0.977 | 0.94 | 0.991 |
| Average_FA | 0.979 | 0.947 | 0.992 |
| BCC | 0.972 | 0.929 | 0.989 |
| CC | 0.966 | 0.912 | 0.987 |
| CGC | 0.94 | 0.845 | 0.977 |
| CGH | 0.935 | 0.834 | 0.975 |
| CR | 0.975 | 0.936 | 0.99 |
| CST | 0.912 | 0.769 | 0.966 |
| EC | 0.985 | 0.961 | 0.994 |
| FX | 0.967 | 0.907 | 0.988 |
| FXST | 0.93 | 0.82 | 0.973 |
| GCC | 0.948 | 0.867 | 0.98 |
| IC | 0.977 | 0.939 | 0.991 |
| IFO | 0.974 | 0.932 | 0.99 |
| PCR | 0.992 | 0.977 | 0.997 |
| PLIC | 0.957 | 0.89 | 0.983 |
| PTR | 0.978 | 0.945 | 0.992 |
| RLIC | 0.966 | 0.913 | 0.987 |
| SCC | 0.952 | 0.877 | 0.981 |
| SCR | 0.985 | 0.959 | 0.995 |
| SFO | 0.978 | 0.945 | 0.992 |
| SLF | 0.984 | 0.958 | 0.994 |
| SS | 0.963 | 0.904 | 0.986 |
| UNC | 0.983 | 0.957 | 0.993 |

Supplementary Table 19: Intraclass correlation coefficients of AD (axial diffusivity) measures for 19 healthy individuals scanned at baseline and 3 months.

| ADNI 3-month | N=19 (baseline, 6 months) | |  |
| --- | --- | --- | --- |
| ROI (MD) | **ICC** | **CI lowerbound** | **CI upperbound** |
| ACR | 0.959 | 0.895 | 0.984 |
| ALIC | 0.986 | 0.965 | 0.995 |
| Average_FA | 0.982 | 0.954 | 0.993 |
| BCC | 0.988 | 0.97 | 0.996 |
| CC | 0.978 | 0.929 | 0.992 |
| CGC | 0.961 | 0.894 | 0.985 |
| CGH | 0.955 | 0.883 | 0.982 |
| CR | 0.984 | 0.956 | 0.994 |
| CST | 0.848 | 0.602 | 0.942 |
| EC | 0.988 | 0.97 | 0.995 |
| FX | 0.979 | 0.922 | 0.993 |
| FXST | 0.933 | 0.828 | 0.974 |
| GCC | 0.965 | 0.894 | 0.987 |
| IC | 0.982 | 0.955 | 0.993 |
| IFO | 0.939 | 0.845 | 0.976 |
| PCR | 0.994 | 0.981 | 0.998 |
| PLIC | 0.941 | 0.847 | 0.977 |
| PTR | 0.983 | 0.957 | 0.993 |
| RLIC | 0.981 | 0.95 | 0.993 |
| SCC | 0.946 | 0.844 | 0.98 |
| SCR | 0.987 | 0.962 | 0.995 |
| SFO | 0.989 | 0.971 | 0.996 |
| SLF | 0.99 | 0.975 | 0.996 |
| SS | 0.983 | 0.956 | 0.993 |
| UNC | 0.987 | 0.965 | 0.995 |

Supplementary Table 20: Intraclass correlation coefficients of MD (mean diffusivity) measures for 19 healthy individuals scanned at baseline and 3 months.

| ADNI 3-month | N=19 (baseline, 6 months) | |  |
| --- | --- | --- | --- |
| ROI (RD) | **ICC** | **CI lowerbound** | **CI upperbound** |
| ACR | 0.977 | 0.937 | 0.991 |
| ALIC | 0.99 | 0.972 | 0.996 |
| Average_FA | 0.982 | 0.949 | 0.994 |
| BCC | 0.987 | 0.945 | 0.996 |
| CC | 0.98 | 0.875 | 0.994 |
| CGC | 0.959 | 0.869 | 0.985 |
| CGH | 0.958 | 0.892 | 0.984 |
| CR | 0.987 | 0.959 | 0.995 |
| CST | 0.827 | 0.549 | 0.934 |
| EC | 0.989 | 0.971 | 0.996 |
| FX | 0.98 | 0.925 | 0.993 |
| FXST | 0.935 | 0.835 | 0.975 |
| GCC | 0.972 | 0.87 | 0.991 |
| IC | 0.983 | 0.956 | 0.993 |
| IFO | 0.919 | 0.794 | 0.969 |
| PCR | 0.994 | 0.982 | 0.998 |
| PLIC | 0.924 | 0.801 | 0.971 |
| PTR | 0.985 | 0.962 | 0.994 |
| RLIC | 0.985 | 0.962 | 0.994 |
| SCC | 0.945 | 0.823 | 0.981 |
| SCR | 0.987 | 0.965 | 0.995 |
| SFO | 0.987 | 0.966 | 0.995 |
| SLF | 0.99 | 0.974 | 0.996 |
| SS | 0.988 | 0.969 | 0.995 |
| UNC | 0.988 | 0.967 | 0.995 |

Supplementary Table 21: Intraclass correlation coefficients of RD (radial diffusivity) measures for 19 healthy individuals scanned at baseline and 3 months.

| ADNI 6-month v 3-month | N=19 (3 months, 6months) | |  |
| --- | --- | --- | --- |
| ROI (FA) | **ICC** | **CI lowerbound** | **CI upperbound** |
| ACR | 0.984 | 0.958 | 0.994 |
| ALIC | 0.961 | 0.899 | 0.985 |
| Average_FA | 0.985 | 0.96 | 0.994 |
| BCC | 0.964 | 0.908 | 0.986 |
| CC | 0.972 | 0.928 | 0.989 |
| CGC | 0.919 | 0.792 | 0.969 |
| CGH | 0.889 | 0.713 | 0.957 |
| CR | 0.991 | 0.976 | 0.996 |
| CST | 0.94 | 0.844 | 0.977 |
| EC | 0.985 | 0.961 | 0.994 |
| FX | 0.949 | 0.869 | 0.98 |
| FXST | 0.964 | 0.908 | 0.986 |
| GCC | 0.968 | 0.919 | 0.988 |
| IC | 0.97 | 0.922 | 0.988 |
| IFO | 0.899 | 0.724 | 0.962 |
| PCR | 0.992 | 0.98 | 0.997 |
| PLIC | 0.918 | 0.788 | 0.968 |
| PTR | 0.986 | 0.965 | 0.995 |
| RLIC | 0.981 | 0.947 | 0.993 |
| SCC | 0.966 | 0.913 | 0.987 |
| SCR | 0.988 | 0.97 | 0.995 |
| SFO | 0.945 | 0.859 | 0.979 |
| SLF | 0.985 | 0.961 | 0.994 |
| SS | 0.979 | 0.948 | 0.992 |
| UNC | 0.992 | 0.979 | 0.997 |

Supplementary Table 22: Intraclass correlation coefficients of FA measures for 19 healthy individuals scanned at 3 months and 6 months after baseline.

| ADNI 3-month | N=19 (3 months, 6months) | |  |
| --- | --- | --- | --- |
| ROI (AD) | **ICC** | **CI-lowerbound** | **CI-upperbound** |
| ACR | 0.95 | 0.871 | 0.981 |
| ALIC | 0.986 | 0.965 | 0.995 |
| Average_FA | 0.99 | 0.974 | 0.996 |
| BCC | 0.958 | 0.891 | 0.984 |
| CC | 0.958 | 0.891 | 0.984 |
| CGC | 0.963 | 0.905 | 0.986 |
| CGH | 0.922 | 0.802 | 0.97 |
| CR | 0.983 | 0.957 | 0.993 |
| CST | 0.927 | 0.813 | 0.972 |
| EC | 0.988 | 0.968 | 0.995 |
| FX | 0.964 | 0.906 | 0.986 |
| FXST | 0.945 | 0.823 | 0.981 |
| GCC | 0.952 | 0.877 | 0.981 |
| IC | 0.978 | 0.942 | 0.991 |
| IFO | 0.974 | 0.933 | 0.99 |
| PCR | 0.994 | 0.986 | 0.998 |
| PLIC | 0.961 | 0.897 | 0.985 |
| PTR | 0.982 | 0.955 | 0.993 |
| RLIC | 0.973 | 0.931 | 0.99 |
| SCC | 0.981 | 0.952 | 0.993 |
| SCR | 0.989 | 0.973 | 0.996 |
| SFO | 0.995 | 0.987 | 0.998 |
| SLF | 0.993 | 0.981 | 0.997 |
| SS | 0.974 | 0.932 | 0.99 |
| UNC | 0.99 | 0.975 | 0.996 |

Supplementary Table 23: Intraclass correlation coefficients of AD (axial diffusivity) measures for 19 healthy individuals scanned at 3 months and 6 months after baseline.

| ADNI 3-month | N=19 (3 months, 6months) | |  |
| --- | --- | --- | --- |
| ROI (MD) | **ICC** | **CI lowerbound** | **CI upperbound** |
| ACR | 0.927 | 0.809 | 0.972 |
| ALIC | 0.991 | 0.977 | 0.997 |
| Average_FA | 0.918 | 0.787 | 0.968 |
| BCC | 0.896 | 0.73 | 0.96 |
| CC | 0.862 | 0.642 | 0.947 |
| CGC | 0.915 | 0.779 | 0.967 |
| CGH | 0.879 | 0.687 | 0.954 |
| CR | 0.965 | 0.909 | 0.987 |
| CST | 0.734 | 0.309 | 0.897 |
| EC | 0.981 | 0.952 | 0.993 |
| FX | 0.36 | -0.661 | 0.754 |
| FXST | 0.94 | 0.845 | 0.977 |
| GCC | 0.927 | 0.811 | 0.972 |
| IC | 0.977 | 0.94 | 0.991 |
| IFO | 0.941 | 0.847 | 0.977 |
| PCR | 0.991 | 0.977 | 0.997 |
| PLIC | 0.956 | 0.886 | 0.983 |
| PTR | 0.938 | 0.84 | 0.976 |
| RLIC | 0.965 | 0.909 | 0.986 |
| SCC | 0.772 | 0.408 | 0.912 |
| SCR | 0.966 | 0.911 | 0.987 |
| SFO | 0.964 | 0.908 | 0.986 |
| SLF | 0.959 | 0.893 | 0.984 |
| SS | 0.957 | 0.888 | 0.983 |
| UNC | 0.988 | 0.969 | 0.995 |

Supplementary Table 24: Intraclass correlation coefficients of MD (mean diffusivity) measures for 19 healthy individuals scanned at 3 months and 6 months after baseline.

| ADNI 3-month | N=19 (3 months, 6months) | |  |
| --- | --- | --- | --- |
| ROI (RD) | **ICC** | **CI lowerbound** | **CI upperbound** |
| ACR | 0.979 | 0.947 | 0.992 |
| ALIC | 0.991 | 0.976 | 0.996 |
| Average_FA | 0.995 | 0.985 | 0.998 |
| BCC | 0.992 | 0.979 | 0.997 |
| CC | 0.993 | 0.982 | 0.998 |
| CGC | 0.986 | 0.952 | 0.995 |
| CGH | 0.937 | 0.838 | 0.976 |
| CR | 0.994 | 0.986 | 0.998 |
| CST | 0.892 | 0.718 | 0.959 |
| EC | 0.991 | 0.978 | 0.997 |
| FX | 0.967 | 0.916 | 0.987 |
| FXST | 0.97 | 0.923 | 0.988 |
| GCC | 0.976 | 0.937 | 0.991 |
| IC | 0.988 | 0.969 | 0.995 |
| IFO | 0.919 | 0.794 | 0.969 |
| PCR | 0.998 | 0.995 | 0.999 |
| PLIC | 0.96 | 0.896 | 0.984 |
| PTR | 0.992 | 0.978 | 0.997 |
| RLIC | 0.988 | 0.966 | 0.995 |
| SCC | 0.991 | 0.976 | 0.996 |
| SCR | 0.992 | 0.979 | 0.997 |
| SFO | 0.981 | 0.952 | 0.993 |
| SLF | 0.994 | 0.984 | 0.998 |
| SS | 0.986 | 0.964 | 0.995 |
| UNC | 0.995 | 0.988 | 0.998 |

Supplementary Table 25: Intraclass correlation coefficients of RD (radial diffusivity) measures for 19 healthy individuals scanned at 3 months and 6 months after baseline.

| (baseline, 24 hours, 72 hours) | |  |  |
| --- | --- | --- | --- |
| ROI (FA) | **ICC** | **CI-lowerbound** | **CI-upperbound** |
| ACR | 0.932 | 0.801 | 0.982 |
| ALIC | 0.849 | 0.576 | 0.959 |
| AverageFA | 0.974 | 0.926 | 0.993 |
| BCC | 0.864 | 0.618 | 0.963 |
| CC | 0.877 | 0.654 | 0.966 |
| CGC | 0.898 | 0.711 | 0.972 |
| CGH | 0.744 | 0.309 | 0.929 |
| CR | 0.904 | 0.727 | 0.974 |
| CST | 0.75 | 0.304 | 0.931 |
| EC | 0.892 | 0.697 | 0.971 |
| FX | 0.681 | 0.076 | 0.914 |
| FXST | 0.809 | 0.461 | 0.948 |
| GCC | 0.924 | 0.784 | 0.979 |
| IC | 0.825 | 0.513 | 0.952 |
| IFO | 0.877 | 0.645 | 0.967 |
| PCR | 0.873 | 0.644 | 0.965 |
| PLIC | 0.799 | 0.434 | 0.945 |
| PTR | 0.919 | 0.771 | 0.978 |
| RLIC | 0.886 | 0.679 | 0.969 |
| SCC | 0.895 | 0.704 | 0.971 |
| SCR | 0.899 | 0.715 | 0.972 |
| SFO | 0.626 | 0.046 | 0.893 |
| SLF | 0.902 | 0.723 | 0.973 |
| SS | 0.934 | 0.812 | 0.982 |
| UNC | 0.956 | 0.876 | 0.988 |

Supplementary Table 26: Intraclass correlation coefficients for FA measures of N=10 healthy individuals, age 22-28 years, scanned at baseline, 24 hours and 72 hours

| (baseline, 24 hours) | |  |  |
| --- | --- | --- | --- |
| ROI (FA) | **ICC** | **CI-lowerbound** | **CI-upperbound** |
| ACR | 0.934 | 0.738 | 0.983 |
| ALIC | 0.881 | 0.515 | 0.971 |
| AverageFA | 0.962 | 0.846 | 0.991 |
| BCC | 0.867 | 0.478 | 0.967 |
| CC | 0.885 | 0.53 | 0.971 |
| CGC | 0.937 | 0.739 | 0.984 |
| CGH | 0.754 | -0.078 | 0.94 |
| CR | 0.937 | 0.755 | 0.984 |
| CST | 0.917 | 0.676 | 0.979 |
| EC | 0.933 | 0.727 | 0.984 |
| FX | 0.142 | -3.532 | 0.799 |
| FXST | 0.683 | -0.324 | 0.925 |
| GCC | 0.903 | 0.598 | 0.976 |
| IC | 0.889 | 0.538 | 0.973 |
| IFO | 0.833 | 0.29 | 0.959 |
| PCR | 0.932 | 0.725 | 0.983 |
| PLIC | 0.92 | 0.669 | 0.98 |
| PTR | 0.959 | 0.841 | 0.99 |
| RLIC | 0.893 | 0.557 | 0.974 |
| SCC | 0.924 | 0.691 | 0.981 |
| SCR | 0.939 | 0.765 | 0.985 |
| SFO | 0.813 | 0.312 | 0.952 |
| SLF | 0.926 | 0.696 | 0.982 |
| SS | 0.946 | 0.779 | 0.987 |
| UNC | 0.965 | 0.864 | 0.991 |

Supplementary Table 27: Intraclass correlation coefficients for FA measures of N=10 healthy individuals, age 22-28 years, scanned at baseline and 24 hours .

| (baseline, 72 hours) | |  |  |
| --- | --- | --- | --- |
| ROI (FA) | **ICC** | **CI-lowerbound** | **CI-upperbound** |
| ACR | 0.876 | 0.459 | 0.97 |
| ALIC | 0.774 | 0.151 | 0.943 |
| AverageFA | 0.987 | 0.921 | 0.997 |
| BCC | 0.819 | 0.331 | 0.954 |
| CC | 0.838 | 0.397 | 0.959 |
| CGC | 0.869 | 0.47 | 0.968 |
| CGH | 0.656 | -0.15 | 0.91 |
| CR | 0.824 | 0.326 | 0.956 |
| CST | 0.625 | -0.378 | 0.905 |
| EC | 0.827 | 0.361 | 0.956 |
| FX | 0.768 | 0.107 | 0.942 |
| FXST | 0.802 | 0.276 | 0.95 |
| GCC | 0.896 | 0.611 | 0.974 |
| IC | 0.72 | 0.016 | 0.928 |
| IFO | 0.888 | 0.58 | 0.972 |
| PCR | 0.824 | 0.353 | 0.955 |
| PLIC | 0.605 | -0.439 | 0.899 |
| PTR | 0.872 | 0.524 | 0.968 |
| RLIC | 0.86 | 0.473 | 0.965 |
| SCC | 0.859 | 0.476 | 0.964 |
| SCR | 0.805 | 0.277 | 0.951 |
| SFO | 0.506 | -0.37 | 0.862 |
| SLF | 0.885 | 0.142 | 0.939 |
| SS | 0.896 | 0.607 | 0.974 |
| UNC | 0.956 | 0.827 | 0.989 |

Supplementary Table 28: Intraclass correlation coefficients for FA measures of N=10 healthy individuals, age 22-28 years, scanned at baseline and 72 hours .

| (24 hours, 72 hours) | |  |  |
| --- | --- | --- | --- |
| ROI (FA) | **ICC** | **CI-lowerbound** | **CI-upperbound** |
| ACR | 0.898 | 0.589 | 0.975 |
| ALIC | 0.719 | 0.018 | 0.927 |
| AverageFA | 0.933 | 0.746 | 0.983 |
| BCC | 0.758 | 0.068 | 0.939 |
| CC | 0.772 | 0.135 | 0.942 |
| CGC | 0.764 | 0.14 | 0.94 |
| CGH | 0.612 | -0.246 | 0.897 |
| CR | 0.831 | 0.378 | 0.957 |
| CST | 0.486 | -0.769 | 0.867 |
| EC | 0.786 | 0.222 | 0.946 |
| FX | 0.498 | -0.878 | 0.873 |
| FXST | 0.69 | -0.187 | 0.922 |
| GCC | 0.871 | 0.518 | 0.967 |
| IC | 0.691 | -0.092 | 0.921 |
| IFO | 0.746 | -0.006 | 0.937 |
| PCR | 0.741 | 0.022 | 0.935 |
| PLIC | 0.662 | -0.227 | 0.914 |
| PTR | 0.81 | 0.239 | 0.953 |
| RLIC | 0.779 | 0.195 | 0.944 |
| SCC | 0.788 | 0.185 | 0.947 |
| SCR | 0.824 | 0.35 | 0.955 |
| SFO | 0.301 | -1.408 | 0.819 |
| SLF | 0.763 | 0.142 | 0.939 |
| SS | 0.876 | 0.532 | 0.969 |
| UNC | 0.88 | 0.55 | 0.97 |

Supplementary Table 29: Intraclass correlation coefficients for FA measures of N=10 healthy individuals, age 22-28 years, scanned at 24 hours and 72 hours after baseline.
