# Supplementary material for: Widespread white matter microstructural differences in schizophrenia across 4322 individuals: results from the ENIGMA Schizophrenia DTI Working Group
Source: Mol Psychiatry. 2017 Oct 17;23(5):1261–9. doi: 10.1038/mp.2017.170 (PMC5984078; doi:10.1038/mp.2017.170)
Supplement: Supplementary Information [file mp2017170x2.pdf]

Supplementary Information

Supplementary Note 1. Further description of ENGMA-DTI protocols
Supplementary note 2. Controlling for core and periphery FA
Supplementary note 3. Meta-analysis description
Supplementary Note 4. Power analysis
Supplementary Note 5. Diffusivity analysis
Supplementary Note 6. Sex-specific effect sizes: Male/female cases vs controls
Supplementary Note 7. Acknowledgements and conflicts of interest

Supplementary Figure 1: Map of ENIGMA-Schizophrenia DTI sites
Supplementary Figure 2: Cohen's *d* effect sizes, after meta-analysis, for FA differences between individuals with schizophrenia and healthy controls, **covarying for average FA**. Age, sex, age×sex, age^2 and age^2×sex, included as covariates. Error bars represent the 95% confidence interval. Orange bars represent significance after Bonferroni correction threshold of 0.05/25 = 0.002.
Supplementary Figure 3: Cohen's *d* effect sizes, after meta-analysis, for FA differences between individuals with schizophrenia and healthy controls, **covarying for core FA**. Age, sex, age×sex, age^2 and age^2×sex, included as covariates. Error bars represent the 95% confidence interval. Orange bars represent significance after Bonferroni correction threshold of 0.05/25 = 0.002.
Supplementary figure 4: Cohen's *d* effect sizes, after meta-analysis, for FA differences between individuals with schizophrenia and healthy controls, **covarying for periphery FA**. Age, sex, age×sex, age^2 and age^2×sex, included as covariates. Error bars represent the 95% confidence interval.

Supplementary figure 5: Meta-analysis forest plots for each ROI
Supplementary figure 6. Cohen's *d* effect sizes, after meta-analysis, for MD differences between schizophrenia patients and healthy controls, including age, sex, age×sex, age^2 and age^2×sex, as covariates. Error bars represent the 95% confidence interval. Significant regions ($p < 0.05/25 = 0.002$) are indicated with an asterisk (*).

Supplementary figure 7. Cohen's *d* effect sizes, after meta-analysis, for RD differences between schizophrenia patients and healthy controls, including age, sex, age×sex, age^2 and age^2×sex, as covariates. Error bars represent the 95% confidence interval. Significant regions ($p < 0.05/25 = 0.002$) are indicated with an asterisk (*).

Supplementary table 1: DTI acquisition protocols for each site
Supplementary table 2: ENIGMA-Schizophrenia DTI site demographics
Supplementary table 3: ENIGMA-Schizophrenia DTI clinical information
Supplementary table 4. Lateralized results for case/control FA differences
Supplementary table 5: Partial correlation betas (meta b), and p-values for symptom severity as measured by SAPS and SANS scores
Supplementary table 6: Female only. Cohen's d effect sizes, standard error (SE), p-values, confidence intervals (CI) after meta-analysis for FA differences between schizophrenia and healthy control females.
Supplementary table 7: Male only. Cohen's d effect sizes, standard error (SE), p-values, confidence intervals (CI) after meta-analysis for FA differences between schizophrenia and healthy control males.
Supplementary table 8: Lateralized meta-analysis results after covarying for average FA
Supplementary table 9: Lateralized meta-analysis results after covarying for core FA
Supplementary table 10 Lateralized meta-analysis results after covarying for peripheral FA
Supplementary table 11:. N80: The total number of samples required, per group, to achieve 80% power to detect group differences using a t-test at the threshold of p<0.05 (two-tailed).
Supplementary table 12. Lateralized results for MD differences in 1,954 healthy controls and 1,255 schizophrenia patients
Supplementary table 13. Lateralized results for RD differences in 1,956 healthy controls and 1,252 schizophrenia patients
Supplementary table 14: Intraclass correlation coefficients of FA measures for 19 healthy individuals scanned at baseline, 3 months and 6 months follow-up.
Supplementary table 15: Intraclass correlation coefficients of AD (axial diffusivity) measures for 19 healthy individuals scanned at baseline, 3 months and 6 months follow-up.
Supplementary table 16: Intraclass correlation coefficients of RD (radial diffusivity) measures for 19 healthy individuals scanned at baseline, 3 months and 6 months follow-up.

Supplementary table 17: Intraclass correlation coefficients of MD (mean diffusivity) measures for 19 healthy individuals scanned at baseline, 3 months and 6 months follow-up.

Supplementary table 18: Intraclass correlation coefficients of FA measures for 19 healthy individuals scanned at baseline and 3 months.

Supplementary table 19: Intraclass correlation coefficients of AD (axial diffusivity) measures for 19 healthy individuals scanned at baseline and 3 months.

Supplementary table 20: Intraclass correlation coefficients of MD (mean diffusivity) measures for 19 healthy individuals scanned at baseline and 3 months.

Supplementary table 21: Intraclass correlation coefficients of RD (radial diffusivity) measures for 19 healthy individuals scanned at baseline and 3 months.

Supplementary table 22: Intraclass correlation coefficients of FA measures for 19 healthy individuals scanned at baseline and 6 months.

Supplementary table 23: Intraclass correlation coefficients of AD (axial diffusivity) measures for 19 healthy individuals scanned at baseline and 3 months.

Supplementary table 24: Intraclass correlation coefficients of MD (mean diffusivity) measures for 19 healthy individuals scanned at baseline and 3 months.

Supplementary table 25: Intraclass correlation coefficients of RD (radial diffusivity) measures for 19 healthy individuals scanned at baseline and 3 months.

Supplementary table 26: Intraclass correlation coefficients of FA measures for 19 healthy individuals scanned at 3 months and 6 months after baseline.

Supplementary table 27: Intraclass correlation coefficients of AD (axial diffusivity) measures for 19 healthy individuals scanned at 3 months and 6 months after baseline.

Supplementary table 28: Intraclass correlation coefficients of MD (mean diffusivity) measures for 19 healthy individuals scanned at 3 months and 6 months after baseline.

Supplementary table 29: Intraclass correlation coefficients of RD (radial diffusivity) measures for 19 healthy individuals scanned at 3 months and 6 months after baseline.

Supplementary table 30: Intraclass correlation coefficients for FA measures of N=10 healthy individuals, age 22-28 years, scanned at baseline, 24 hours and 72 hours

Supplementary table 31: Intraclass correlation coefficients for FA measures of N=10 healthy individuals, age 22-28 years, scanned at baseline and 24 hours .
Supplementary table 32: Intraclass correlation coefficients for FA measures of N=10 healthy individuals, age 22-28 years, scanned at baseline and 72 hours .
Supplementary table 33: Intraclass correlation coefficients for FA measures of N=10 healthy individuals, age 22-28 years, scanned at 24 hours and 72 hours after baseline.

Supplementary Note 1: Further detail of ENIGMA-DTI protocols

Details on study type, scanner and acquisition parameters are provided in supplementary table 3. Preprocessing, including eddy current correction, EPI induced distortion correction, and tensor fitting, was carried out at each site. Image analysis was conducted at each site using tract-based spatial statistics (TBSS) as part of FSL software (Smith et al., 2006).

Individual subject FA maps were aligned to the custom ENIGMA-DTI FA template derived from 400 adult participants scanned across four sites designed for for optimal multi-site harmonization (Jahanshad et al., 2013). FA voxels were then projected onto the ENIGMA-DTI template skeleton. This creates a unique FA skeleton in the same space for each individual in each cohort. The same projection used for the FA images also projects the non-FA (mean axial and radial) images onto the skeleton. Voxels along the individual skeletons were averaged across white matter ROIs. A total of 25 bilateral ROIs were delineated based on the JHU WM atlas, an established WM parcellation derived using deterministic tractography (Hua et al, 2008). A whole-brain WM skeleton was defined according to the tract-based spatial statistic methodology (Smith et al, 2006) and ROI-averaged measures of FA, MD, AD and RD were then calculated by averaging each of these voxel measures over all skeleton voxels encapsulated by a particular ROI. This ensured that voxels at the periphery of a fiber bundle, where residual registration misalignment is typically maximal, were excluded from the ROI average. In other words, ROI averaging was performed based on the core of each fiber bundle, as defined by the WM skeleton.

The multi-subject JHU white matter parcellation atlas (Mori et al., 2008) was used to parcellate regions of interest from the ENIGMA template in MNI space. As certain ROIs may be susceptible to the effects of field of view (FOV) and partial voluming, leading to unstable and unreliable estimates, a reliability analysis based on multiple cohorts with longitudinal data was conducted for each ROI (see supplementary material).

A total of twenty-five bilateral white matter ROIs were extracted from the skeletonized FA images and averaged. Table 1 lists 25 ROIs (some partially overlapping) that were extracted from the skeletonized images, including 5 midsagittal regions (no lateralized

components), and 19 lateralized regions (left and right are averaged to obtain bilateral FA) (Supplementary material). The overall average FA values were calculated by averaging values for the entire white matter skeleton.

ENIGMA-DTI QA/QC protocol consists of visual inspection of the images before and after registration to the ENIGMA template, as well as calculating the average skeleton projection distance. The distance of voxel projection to the ENIGMA skeleton can assess the registration quality between individual images and ENIGMA-DTI template. Higher projection distance may indicate problems with aligning individual brain to the template. After ROI extraction, histograms of FA and diffusivity measures are computed for each ROI.

Abbreviation	Full tract name
AverageFA	Full skeleton average FA
ACR (L+R)	Anterior *corona radiata*
ALIC (L+R)	Anterior limb of internal capsule
BCC	Body of *corpus callosum*
CC (BCC+GCC+SCC)	Corpus callosum
CGC (L+R)	Cingulum (cingulate gyrus)
CGH (L+R)	Cingulum (hippocampal portion)
CR (L+R)	*Corona radiata*
CST (L+R)	Corticospinal tract
EC (L+R)	External capsule

FX	*Fornix*
FXST (L+R)	*Fornix* (cres) / *Stria terminalis*
GCC	***Genu*** of *corpus callosum*
IC (L+R)	Internal capsule
IFO (L+R)	Inferior fronto-occipital fasciculus
PCR (L+R)	Posterior *corona radiata*
PLIC (L+R)	Posterior limb of internal capsule
PTR (L+R)	Posterior thalamic radiation
RLIC (L+R)	Retrolenticular part of internal capsule
SCC	***Splenium*** of *corpus callosum*
SCR (L+R)	Superior *corona radiata*
SFO (L+R)	Superior fronto-occipital fasciculus
SLF (L+R)	Superior longitudinal fasciculus
SS (L+R)	Sagittal *stratum*
UNC (L+R)	*Uncinate* fasciculus

Supplementary figure 1. Map of ENIGMA-Schizophrenia DTI contributing sites. For Australia, ASRB sites should also include Brisbane, Newcastle and Perth.

Supplementary Note 2: Covarying for core and periphery FA

To tease apart regional WM effects from the global differences, post-hoc analysis on a sub-sample of available data from 17 cohorts (1,361 healthy controls and 1,226 schizophrenia patients) was carried out in individual tract ROIs as above but additionally covarying for 1) average FA across the entire skeleton, 2) average FA within the core of the skeleton within which ROIs are defined, or 3) the average FA within the periphery of the skeleton, areas on the skeleton not included in the ROIs of the JHU atlas. Core FA was calculated by computing the weighted average of all non-overlapping ROIs. Periphery FA was calculated by subtracting weighted core FA (N core voxels/N periphery voxels x core FA) from the weighted average FA voxels (N average FA voxels/N periphery FA voxels x averageFA).

Supplementary Note 3: Meta-analysis description

Using this a random-effects inverse-variance weighted meta-analysis, results from statistical tests performed at each individual site were combined and the overall statistical effect size was calculated as the weighted average of the effects from the individual cohorts as determined by the standard error of the effect.

Supplementary Note 4: Power analysis

A post-hoc power analysis, conducted using G*Power v3.1, revealed that the current sample of 1,984 individuals with schizophrenia and 2,391 healthy controls achieved 80% power to detect Cohen's *d* effect sizes as small as d=0.085 at the standard alpha level of p<0.05 (two-tailed), and 80% power to detect Cohen's *d* effect sizes of d=0.12 at the study's Bonferroni-corrected threshold of p<0.002. Across all ROIs, we estimated N80: the total number of samples required, per group, to achieve 80% power to detect group differences using a t-test at the threshold of p<0.05 (two-tailed). N80 ranged from 104-13,581 (See supplementary table 5).

Supplementary Note 5: Diffusivity analysis

Available diffusivity images, including mean, radial and axial diffusivity, were skeletonized and relevant ROI information was extracted according to the ENIGMA-DTI template. A random effects, inverse variance weighted meta-analysis was conducted to combine results. A random effects, inverse variance weighted meta-analysis was conducted to combine results.

Supplementary Note 6: Sex-Specific Effect Sizes: Male / Female Cases vs Controls

We observed significant differences in effect size when analyzing males and females separately, with females showing significantly larger effects for decreased FA (paired t=-5.21, p=0.0001).

For females, 20 of the 25 ROIs showed significantly lower FA for patients at the p=0.002 significance threshold (See supplementary table 6).
For males, 14 of the 25 ROIs showed significantly decreased FA for patients at the p=0.002 significance threshold (See supplementary table 7).

Supplementary Note 7: Acknowledgements and conflicts of interest

The **ENIGMA-Schizophrenia working** group gratefully acknowledges support from the NIH BD2K award, U54EB020403.
TOP: The TOP study was supported by the Research Council of Norway (#213837, #223273, #229129), South-East Norway Health Authority (#2013-123), South-Eastern Norway Regional Health Authority (#2014097) and KG Jebsen Foundation. The research leading to these results has received funding from the European Community's Seventh Framework Programme (FP7/2007-2013) under grant agreement n° 602450 (IMAGEMEND).
Osaka: Some computations were performed at the Research Center for Computational Science, Okazaki, Japan.
Brain and Mind Research Institute: This study was supported by the following National Health and Medical Research Council funding sources: Programme Grant (no. 566529), Centres of Clinical Research Excellence Grant (no. 264611), Australia Fellowship (no. 511921) and Clinical Research Fellowship (no. 402864).
Zora Kikinis (CIDAR): Work supported by grant R21MH106793

Paul Klauser (ASRB) was supported by the Swiss National Science Foundation (SNSF) and the Swiss Society for Medicine and Biology Scholarships (148384) and the National Center for Competence in Research — SYNAPSY — funded by the SNSF.
Robert W. McCarley is upported by NIMH 5P50MH080272
Edinburgh: Supported by Wellcome Trust 104036/Z/14/Z and by Sackler Foundation
Raquelle I. Mesholam-Gately Supported by NIMH 5P50MH080272
Roberto Roiz “We thank IDIVAL Neuroimaging Unit for its help in the technical execution of this work”
Peter Savadjiev was supported by NARSAD Young Investigator Award Number 22591, from the Brain and Behavior Research Foundation.
Ulrich Schall was supported by the National Health & Medical Research Council of Australia and received infrastructure funding of the New South Wales State Government through the Hunter Medical Research Institute and the Schizophrenia Research Institute.
Martha E. Shenton (CIDAR) is supported by VA Merit Award (I01 RX00928)
Gianfranco Spalletta (Rome) was funded by the Italian Ministry of Health grant RC 12-13-14-15/A
FBIRN This work was supported by the National Center for Research Resources at the National Institutes of Health (grant numbers: NIH 1 U24 U24 RR021992 (Function Biomedical Informatics Research Network), NIH 1 U24 RR025736-01 (Biomedical Informatics Research Network Coordinating Center; http://www.birncommunity.org), and he National Institute Of Biomedical Imaging And Bioengineering (NIBIB) of the National Institutes of Health under Award Number U54EB020403. The funding sources had no role in the study design, data collection, analysis, and interpretation of the data. We are thankful to Liv McMillan for overall study coordination, Harry Mangalam, Joseph Farran, and Adam Brenner, for administering the University of California, Irvine High-Performance Computing cluster, and to the research subjects for their participation.
Esther Goudzwaard (FBIRN) was supported by the following Dutch Foundations: A.S.C. Academy, Amsterdam Universiteitsfonds, Bekker-la Bastide-Fonds, Schimmel Schuurman - van Outeren Foundation, Scholten-Cordes Foundation.

Drs Pacigo, Xie, Hyde, Chen, O’Donnell and Hibar have private funding unrelated to the content of this paper.

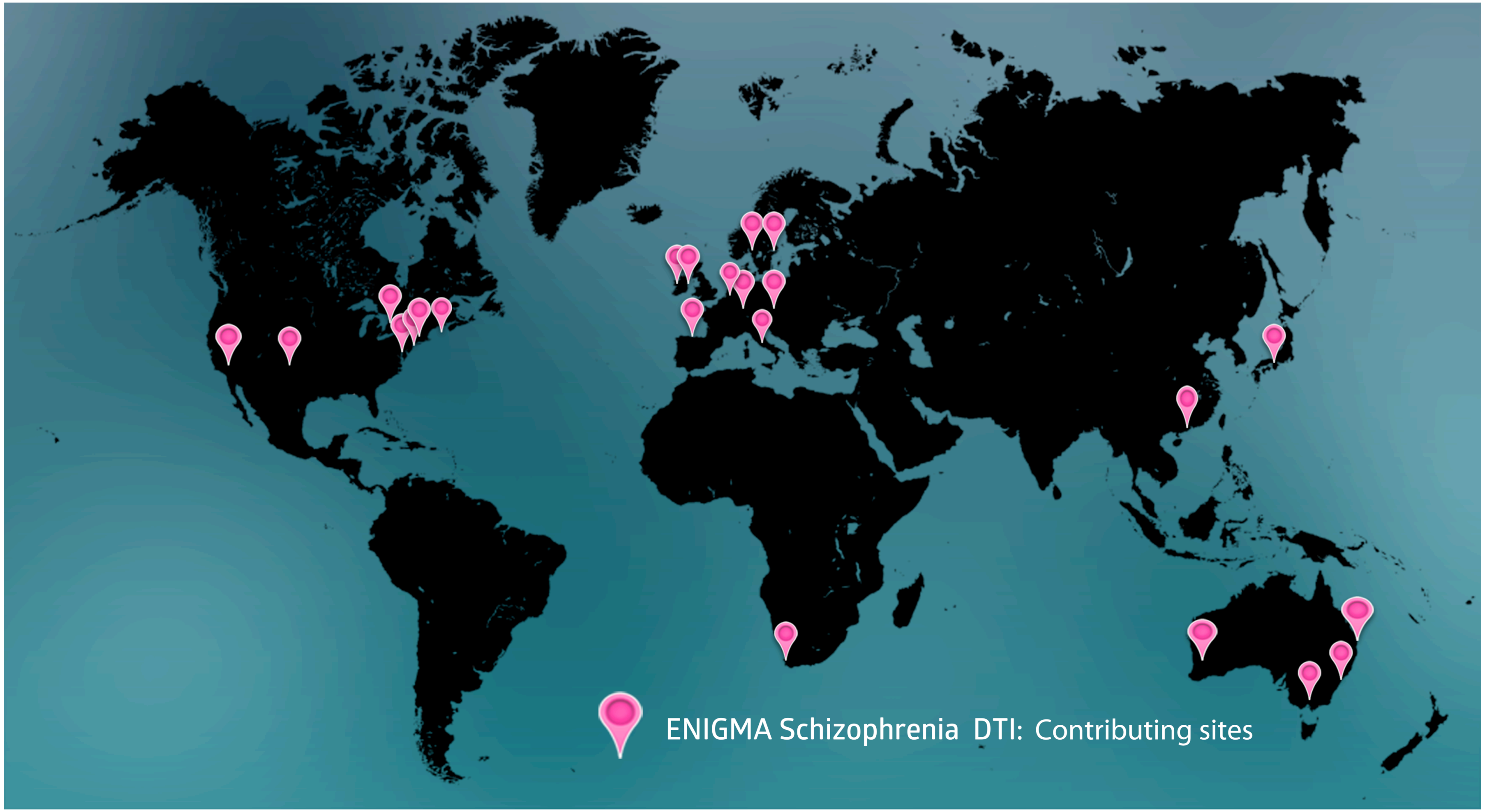

Supplementary figure 1: Map of ENIGMA-Schizophrenia DTI participating sites

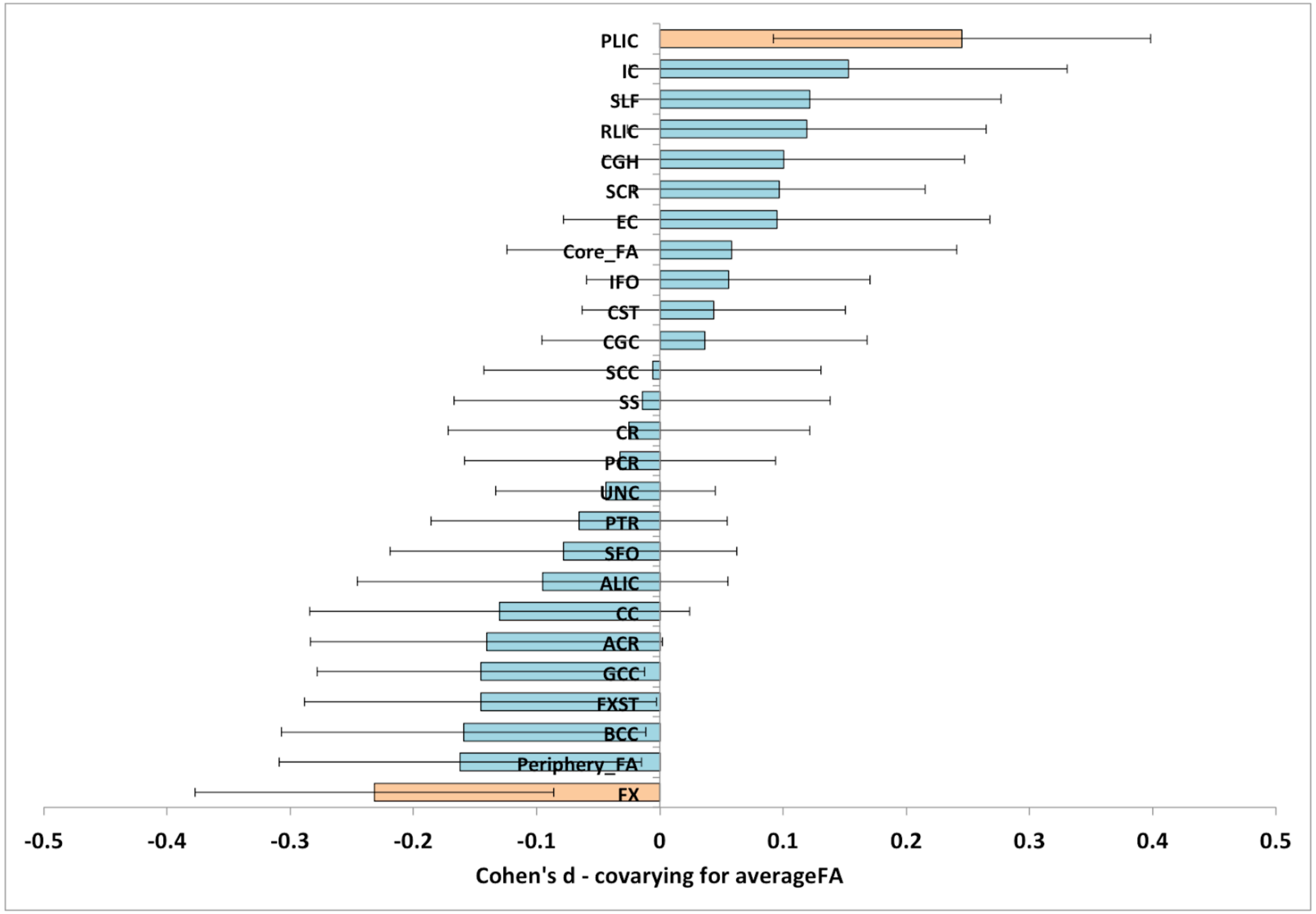

Supplementary figure 2. Cohen's *d* effect sizes, after meta-analysis, for FA differences between individuals with schizophrenia and healthy controls, **covarying for average FA**. Age, sex, age×sex, age and age×sex, included as covariates. Error bars represent the 95% confidence interval. Orange bars represent significance after Bonferroni correction threshold of 0.05/25 = 0.002.

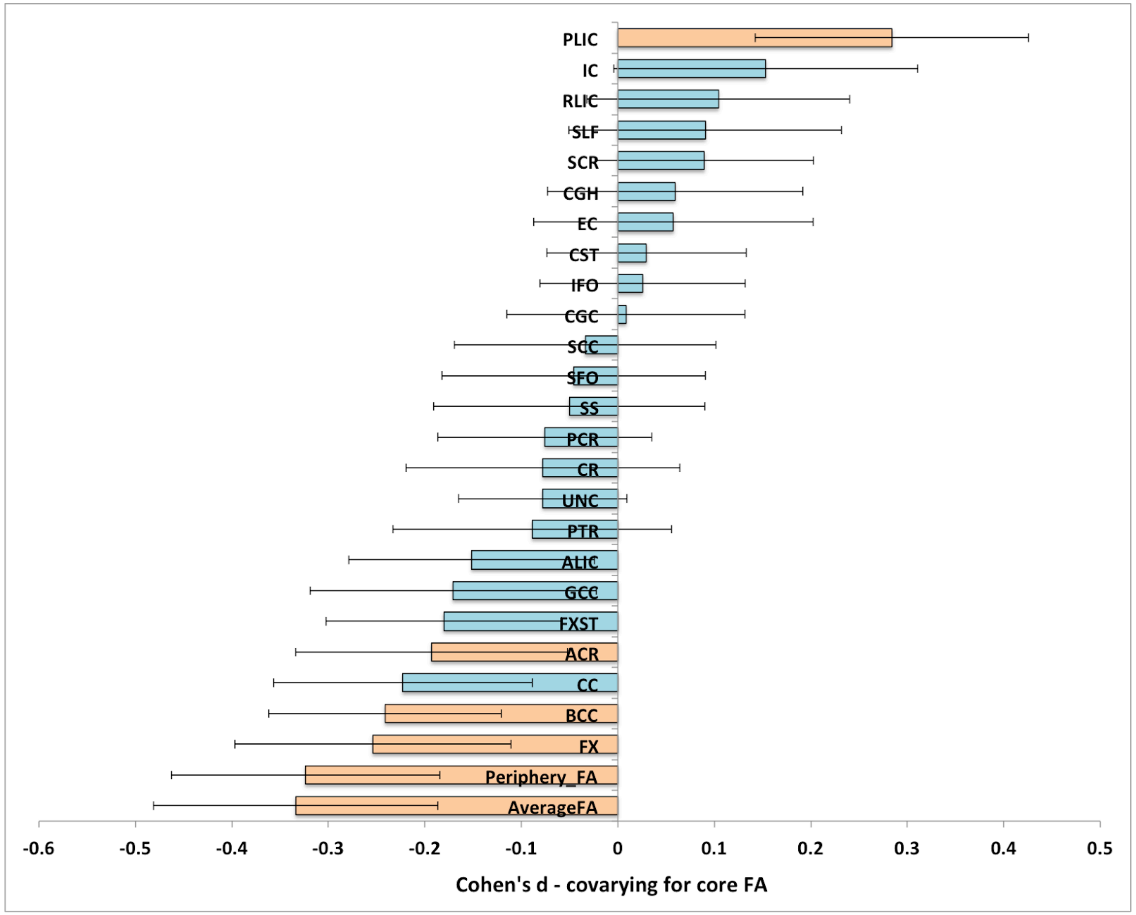

Supplementary figure 3. Cohen's *d* effect sizes, after meta-analysis, for FA differences between individuals with schizophrenia and healthy controls, **covarying for core FA**. Age, sex, age×sex, age and age×sex, included as covariates. Error bars represent the 95% confidence interval. Orange bars represent significance after Bonferroni correction threshold of 0.05/25 = 0.002.

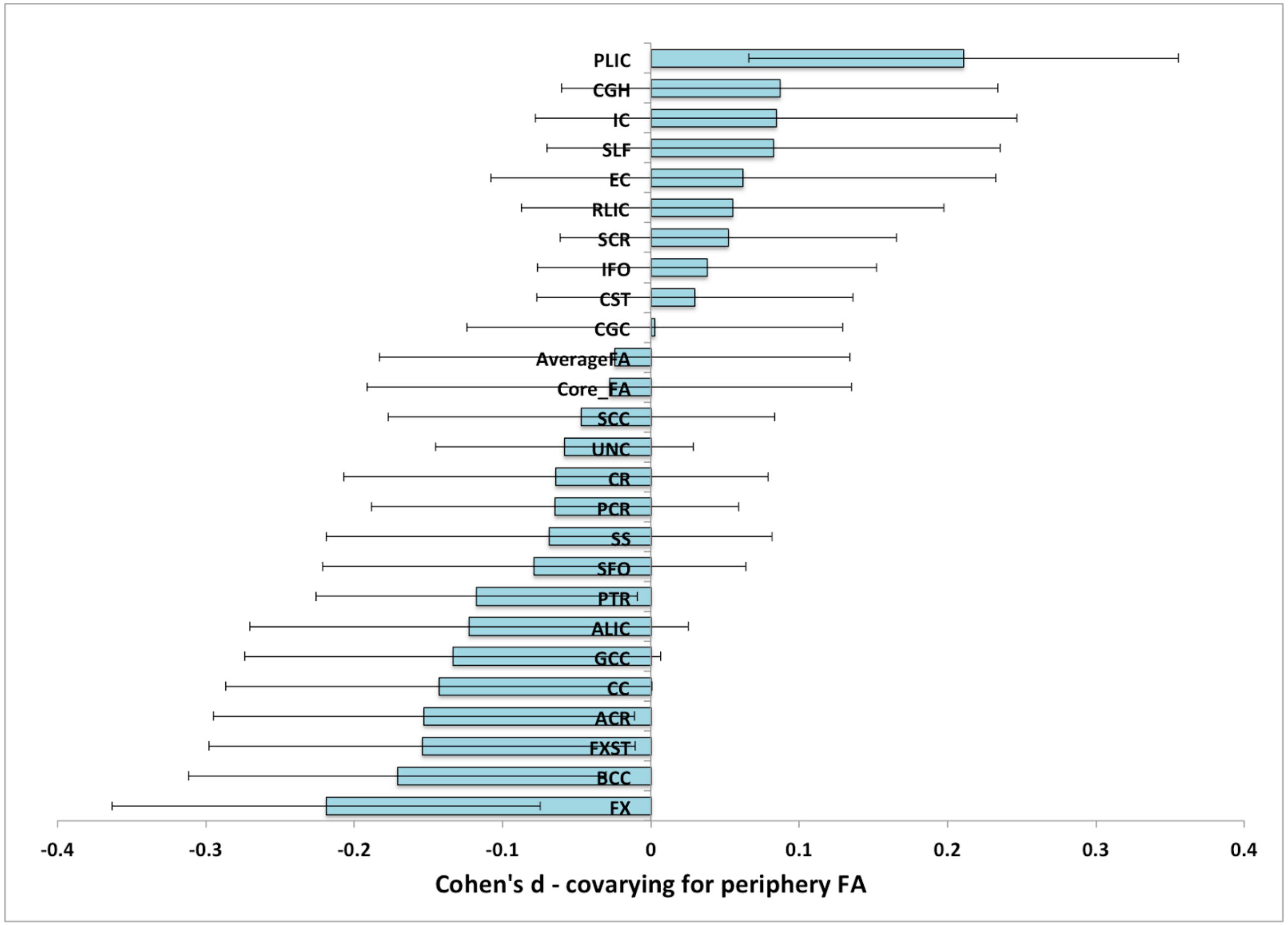

Supplementary figure 4. Cohen's *d* effect sizes, after meta-analysis, for FA differences between individuals with schizophrenia and healthy controls, **covarying for periphery FA**. Age, sex, age×sex, age and age×sex, included as covariates. Error bars represent the 95% confidence interval.

Supplementary figure 5. Meta-analysis forest plots for each ROI

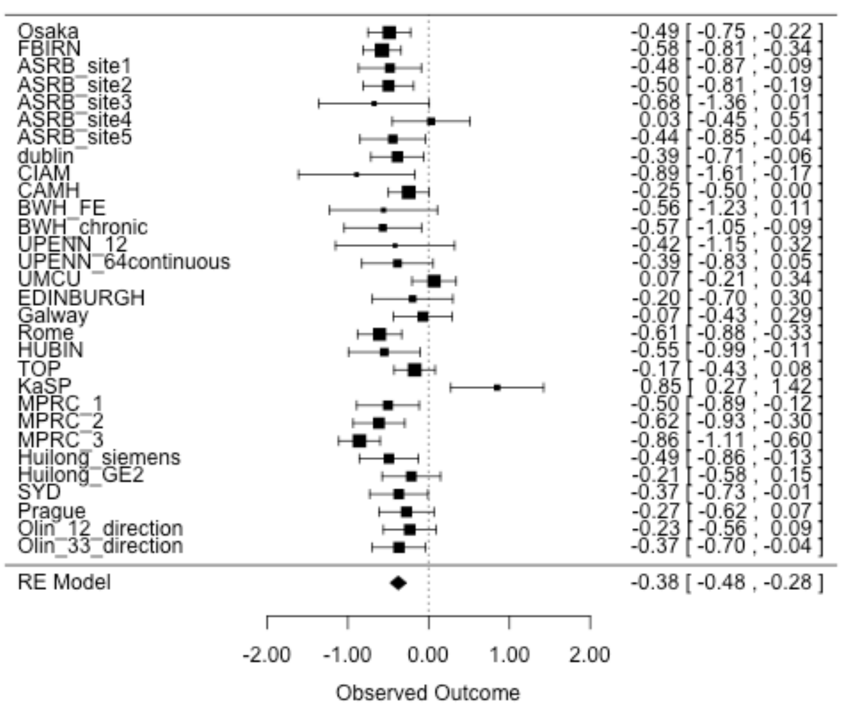

ACR

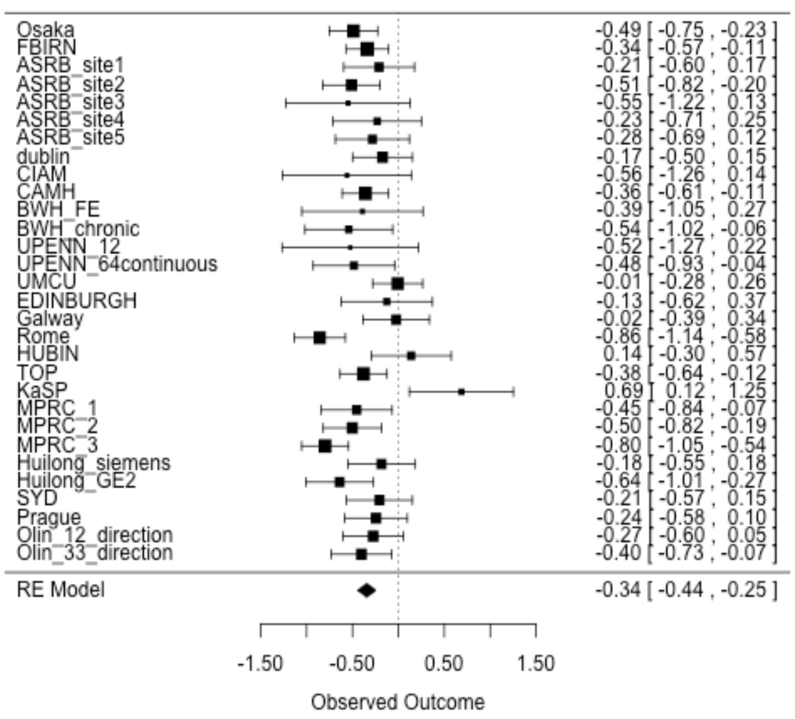

ALIC

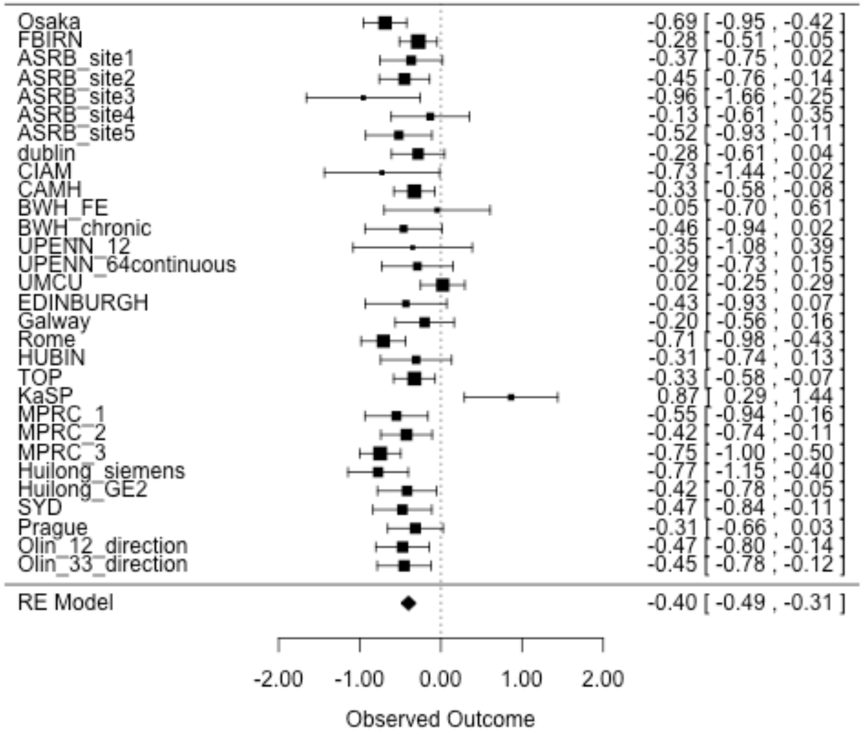

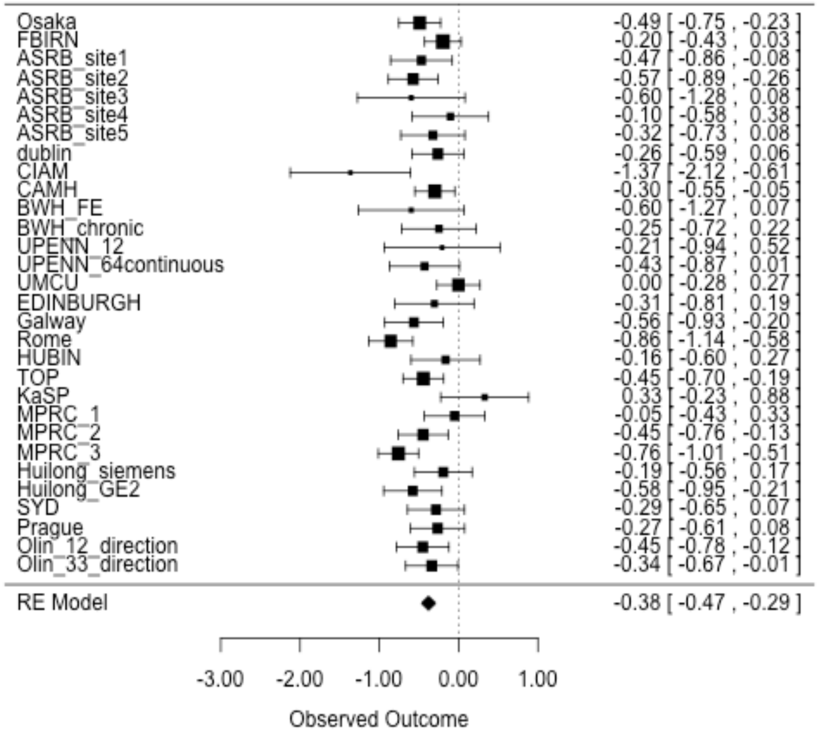

Average FA **BCC**

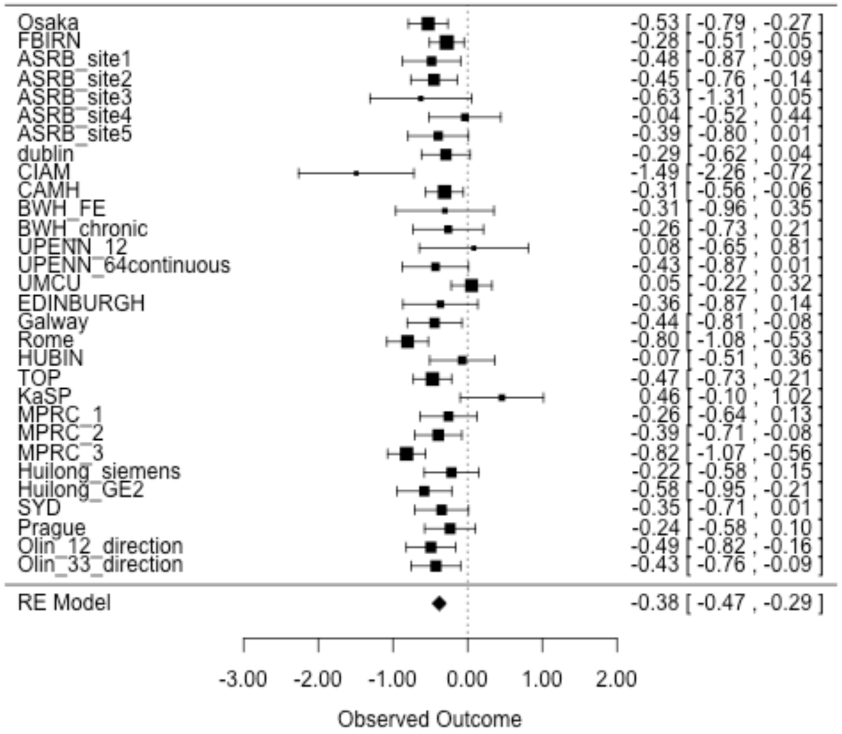
Osaka -0.53 [-0.79 , -0.27]
FBIRN -0.28 [-0.51 , -0.05]
ASRB_site1 -0.48 [-0.87 , -0.09]
ASRB_site2 -0.45 [-0.76 , -0.14]
ASRB_site3 -0.63 [-1.31 , 0.05]
ASRB_site4 -0.04 [-0.52 , 0.44]
ASRB_site5 -0.39 [-0.80 , 0.01]
dublin -0.29 [-0.62 , 0.04]
CIAM -1.49 [-2.26 , -0.72]
CAMH -0.31 [-0.56 , -0.06]
BWH_FE -0.31 [-0.96 , 0.35]
BWH_chronic -0.26 [-0.73 , 0.21]
UPENN_12 0.08 [-0.65 , 0.81]
UPENN_64continuous -0.43 [-0.87 , 0.01]
UMCU 0.05 [-0.22 , 0.32]
EDINBURGH -0.36 [-0.87 , 0.14]
Galway -0.44 [-0.81 , -0.08]
Rome -0.80 [-1.08 , -0.53]
HUBIN -0.07 [-0.51 , 0.36]
TOP -0.47 [-0.73 , -0.21]
KaSP 0.46 [-0.10 , 1.02]
MPRC_1 -0.26 [-0.64 , 0.13]
MPRC_2 -0.39 [-0.71 , -0.08]
MPRC_3 -0.82 [-1.07 , -0.56]
Huilong_siemens -0.22 [-0.58 , 0.15]
Huilong_GE2 -0.58 [-0.95 , -0.21]
SYD -0.35 [-0.71 , 0.01]
Prague -0.24 [-0.58 , 0.10]
Olin_12_direction -0.49 [-0.82 , -0.16]
Olin_33_direction -0.43 [-0.76 , -0.09]
RE Model -0.38 [-0.47 , -0.29]
-3.00 -2.00 -1.00 0.00 1.00 2.00
Observed Outcome

CC

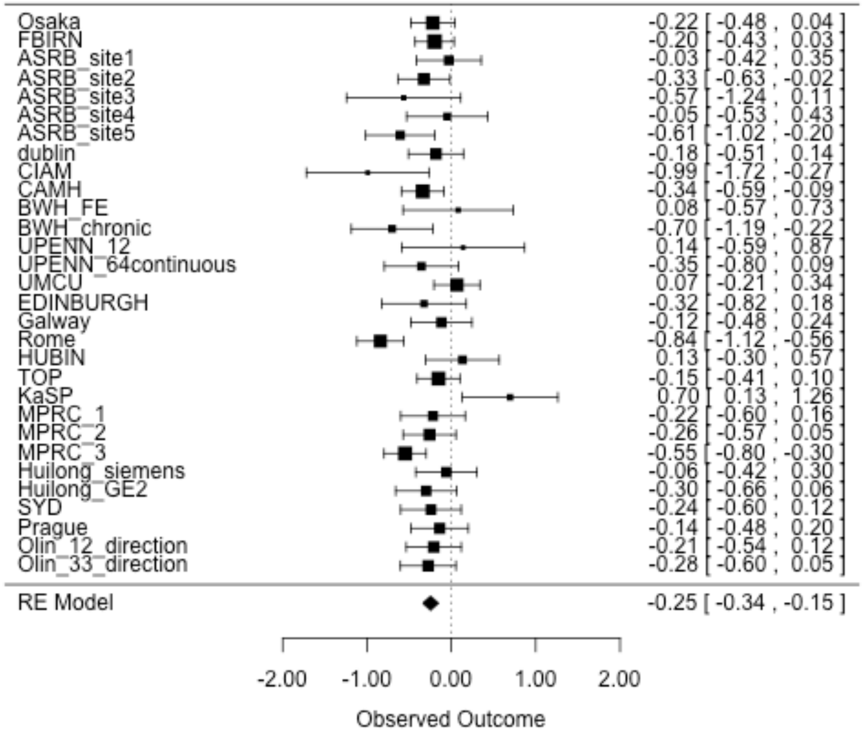
Osaka -0.22 [-0.48 , 0.04]
FBIRN -0.20 [-0.43 , 0.03]
ASRB_site1 -0.03 [-0.42 , 0.35]
ASRB_site2 -0.33 [-0.63 , -0.02]
ASRB_site3 -0.57 [-1.24 , 0.11]
ASRB_site4 -0.05 [-0.53 , 0.43]
ASRB_site5 -0.61 [-1.02 , -0.20]
dublin -0.18 [-0.51 , 0.14]
CIAM -0.99 [-1.72 , -0.27]
CAMH -0.34 [-0.59 , -0.09]
BWH_FE 0.08 [-0.57 , 0.73]
BWH_chronic -0.70 [-1.19 , -0.22]
UPENN_12 0.14 [-0.59 , 0.87]
UPENN_64continuous -0.35 [-0.80 , 0.09]
UMCU 0.07 [-0.21 , 0.34]
EDINBURGH -0.32 [-0.82 , 0.18]
Galway -0.12 [-0.48 , 0.24]
Rome -0.84 [-1.12 , -0.56]
HUBIN 0.13 [-0.30 , 0.57]
TOP -0.15 [-0.41 , 0.10]
KaSP 0.70 [0.13 , 1.26]
MPRC_1 -0.22 [-0.60 , 0.16]
MPRC_2 -0.26 [-0.57 , 0.05]
MPRC_3 -0.55 [-0.80 , -0.30]
Huilong_siemens -0.06 [-0.42 , 0.30]
Huilong_GE2 -0.30 [-0.66 , 0.06]
SYD -0.24 [-0.60 , 0.12]
Prague -0.14 [-0.48 , 0.20]
Olin_12_direction -0.21 [-0.54 , 0.12]
Olin_33_direction -0.28 [-0.60 , 0.05]
RE Model -0.25 [-0.34 , -0.15]
-2.00 -1.00 0.00 1.00 2.00
Observed Outcome

CGC

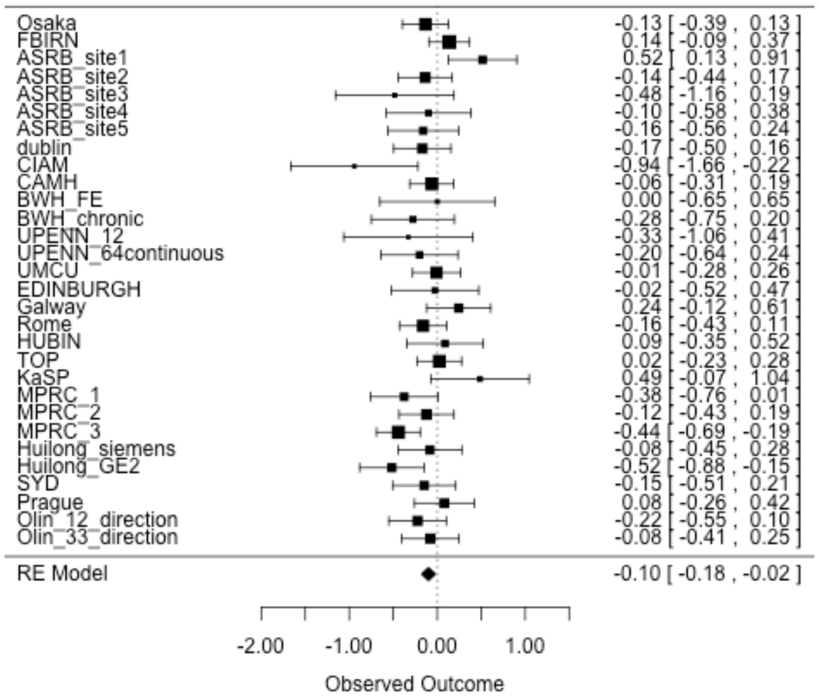
Osaka -0.13 [-0.39 , 0.13]
FBIRN 0.14 [-0.09 , 0.37]
ASRB_site1 0.52 [0.13 , 0.91]
ASRB_site2 -0.14 [-0.44 , 0.17]
ASRB_site3 -0.48 [-1.16 , 0.19]
ASRB_site4 -0.10 [-0.58 , 0.38]
ASRB_site5 -0.16 [-0.56 , 0.24]
dublin -0.17 [-0.50 , 0.16]
CIAM -0.94 [-1.66 , -0.22]
CAMH -0.06 [-0.31 , 0.19]
BWH_FE 0.00 [-0.65 , 0.65]
BWH_chronic -0.28 [-0.75 , 0.20]
UPENN_12 -0.33 [-1.06 , 0.41]
UPENN_64continuous -0.20 [-0.64 , 0.24]
UMCU -0.01 [-0.28 , 0.26]
EDINBURGH -0.02 [-0.52 , 0.47]
Galway 0.24 [-0.12 , 0.61]
Rome -0.16 [-0.43 , 0.11]
HUBIN 0.09 [-0.35 , 0.52]
TOP 0.02 [-0.23 , 0.28]
KaSP 0.49 [-0.07 , 1.04]
MPRC_1 -0.38 [-0.76 , 0.01]
MPRC_2 -0.12 [-0.43 , 0.19]
MPRC_3 -0.44 [-0.69 , -0.19]
Huilong_siemens -0.08 [-0.45 , 0.28]
Huilong_GE2 -0.52 [-0.88 , -0.15]
SYD -0.15 [-0.51 , 0.21]
Prague 0.08 [-0.26 , 0.42]
Olin_12_direction -0.22 [-0.55 , 0.10]
Olin_33_direction -0.08 [-0.41 , 0.25]
RE Model -0.10 [-0.18 , -0.02]
-2.00
-1.00
0.00
1.00
Observed Outcome

CGH

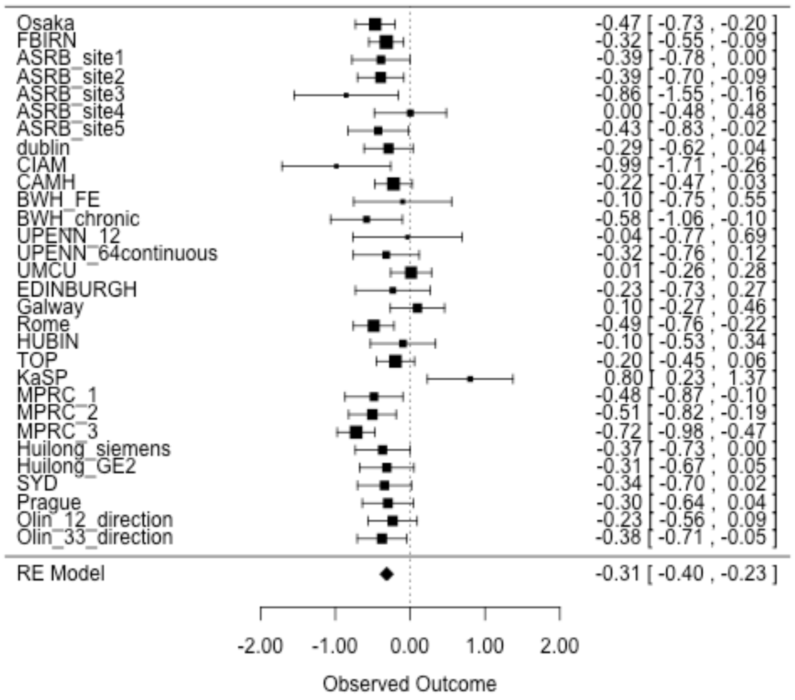
Osaka -0.47 [-0.73 , -0.20]
FBIRN -0.32 [-0.55 , -0.09]
ASRB_site1 -0.39 [-0.78 , 0.00]
ASRB_site2 -0.39 [-0.70 , -0.09]
ASRB_site3 -0.86 [-1.55 , -0.16]
ASRB_site4 0.00 [-0.48 , 0.48]
ASRB_site5 -0.43 [-0.83 , -0.02]
dublin -0.29 [-0.62 , 0.04]
CIAM -0.99 [-1.71 , -0.26]
CAMH -0.22 [-0.47 , 0.03]
BWH_FE -0.10 [-0.75 , 0.55]
BWH_chronic -0.58 [-1.06 , -0.10]
UPENN_12 -0.04 [-0.77 , 0.69]
UPENN_64continuous -0.32 [-0.76 , 0.12]
UMCU 0.01 [-0.26 , 0.28]
EDINBURGH -0.23 [-0.73 , 0.27]
Galway 0.10 [-0.27 , 0.46]
Rome -0.49 [-0.76 , -0.22]
HUBIN -0.10 [-0.53 , 0.34]
TOP -0.20 [-0.45 , 0.06]
KaSP 0.80 [0.23 , 1.37]
MPRC_1 -0.48 [-0.87 , -0.10]
MPRC_2 -0.51 [-0.82 , -0.19]
MPRC_3 -0.72 [-0.98 , -0.47]
Huilong_siemens -0.37 [-0.73 , 0.00]
Huilong_GE2 -0.31 [-0.67 , 0.05]
SYD -0.34 [-0.70 , 0.02]
Prague -0.30 [-0.64 , 0.04]
Olin_12_direction -0.23 [-0.56 , 0.09]
Olin_33_direction -0.38 [-0.71 , -0.05]
RE Model -0.31 [-0.40 , -0.23]
-2.00
-1.00
0.00
1.00
2.00
Observed Outcome

CR

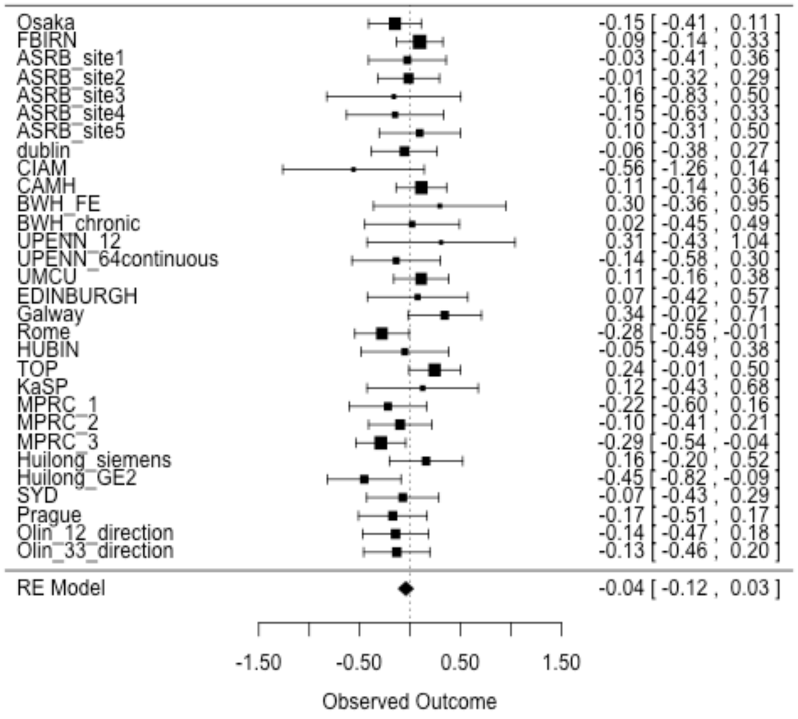
Osaka -0.15 [-0.41 , 0.11]
FBIRN 0.09 [-0.14 , 0.33]
ASRB_site1 -0.03 [-0.41 , 0.36]
ASRB_site2 -0.01 [-0.32 , 0.29]
ASRB_site3 -0.16 [-0.83 , 0.50]
ASRB_site4 -0.15 [-0.63 , 0.33]
ASRB_site5 0.10 [-0.31 , 0.50]
dublin -0.06 [-0.38 , 0.27]
CIAM -0.56 [-1.26 , 0.14]
CAMH 0.11 [-0.14 , 0.36]
BWH_FE 0.30 [-0.36 , 0.95]
BWH_chronic 0.02 [-0.45 , 0.49]
UPENN_12 0.31 [-0.43 , 1.04]
UPENN_64continuous -0.14 [-0.58 , 0.30]
UMCU 0.11 [-0.16 , 0.38]
EDINBURGH 0.07 [-0.42 , 0.57]
Galway 0.34 [-0.02 , 0.71]
Rome -0.28 [-0.55 , -0.01]
HUBIN -0.05 [-0.49 , 0.38]
TOP 0.24 [-0.01 , 0.50]
KaSP 0.12 [-0.43 , 0.68]
MPRC_1 -0.22 [-0.60 , 0.16]
MPRC_2 -0.10 [-0.41 , 0.21]
MPRC_3 -0.29 [-0.54 , -0.04]
Huilong_siemens 0.16 [-0.20 , 0.52]
Huilong_GE2 -0.45 [-0.82 , -0.09]
SYD -0.07 [-0.43 , 0.29]
Prague -0.17 [-0.51 , 0.17]
Olin_12_direction -0.14 [-0.47 , 0.18]
Olin_33_direction -0.13 [-0.46 , 0.20]
RE Model -0.04 [-0.12 , 0.03]
-1.50
-0.50
0.50
1.50
Observed Outcome

CST

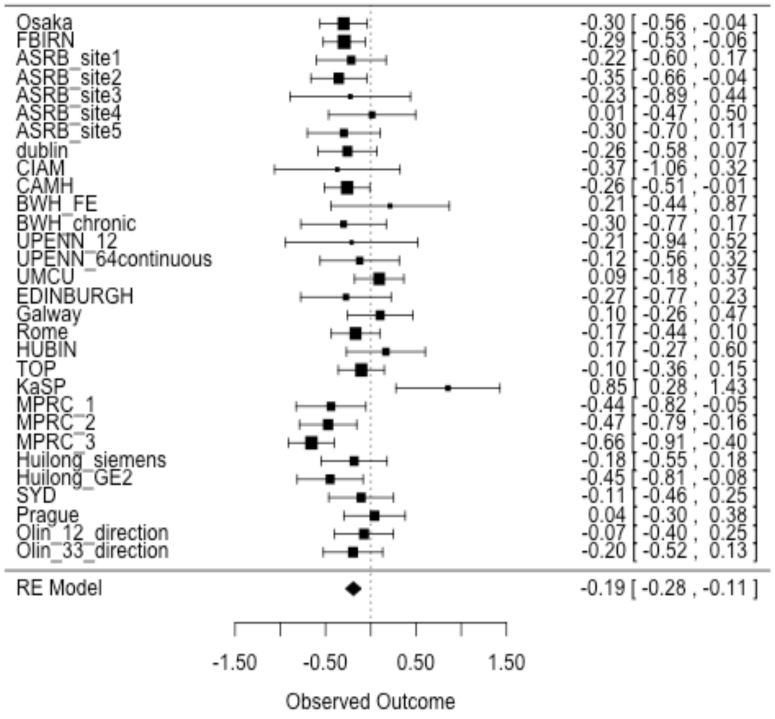
Osaka -0.30 [-0.56 , -0.04]
FBIRN -0.29 [-0.53 , -0.06]
ASRB_site1 -0.22 [-0.60 , 0.17]
ASRB_site2 -0.35 [-0.66 , -0.04]
ASRB_site3 -0.23 [-0.89 , 0.44]
ASRB_site4 0.01 [-0.47 , 0.50]
ASRB_site5 -0.30 [-0.70 , 0.11]
dublin -0.26 [-0.58 , 0.07]
CIAM -0.37 [-1.06 , 0.32]
CAMH -0.26 [-0.51 , -0.01]
BWH_FE 0.21 [-0.44 , 0.87]
BWH_chronic -0.30 [-0.77 , 0.17]
UPENN_12 -0.21 [-0.94 , 0.52]
UPENN_64continuous -0.12 [-0.56 , 0.32]
UMCU 0.09 [-0.18 , 0.37]
EDINBURGH -0.27 [-0.77 , 0.23]
Galway 0.10 [-0.26 , 0.47]
Rome -0.17 [-0.44 , 0.10]
HUBIN 0.17 [-0.27 , 0.60]
TOP -0.10 [-0.36 , 0.15]
KaSP 0.85 [0.28 , 1.43]
MPRC_1 -0.44 [-0.82 , -0.05]
MPRC_2 -0.47 [-0.79 , -0.16]
MPRC_3 -0.66 [-0.91 , -0.40]
Huilong_siemens -0.18 [-0.55 , 0.18]
Huilong_GE2 -0.45 [-0.81 , -0.08]
SYD -0.11 [-0.46 , 0.25]
Prague 0.04 [-0.30 , 0.38]
Olin_12_direction -0.07 [-0.40 , 0.25]
Olin_33_direction -0.20 [-0.52 , 0.13]
RE Model -0.19 [-0.28 , -0.11]
-1.50
-0.50
0.50
1.50
Observed Outcome

EC

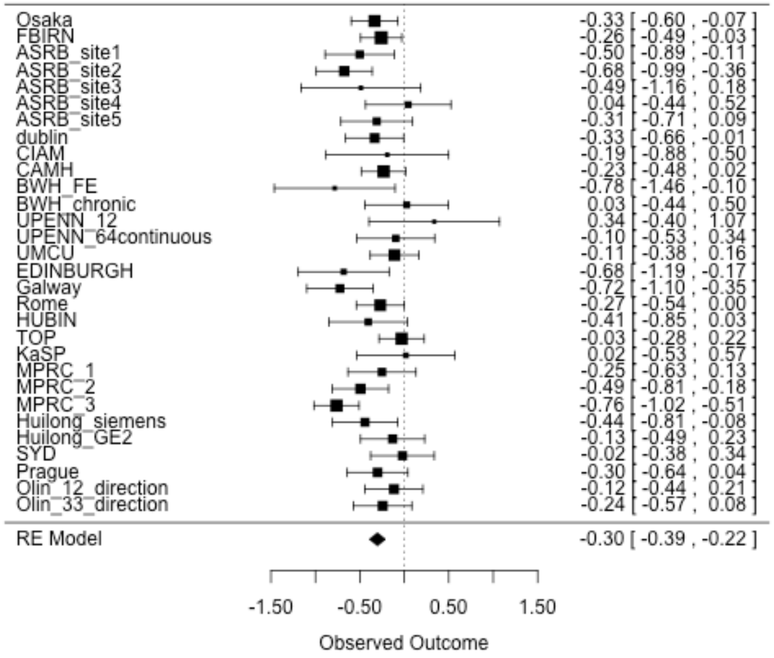
Osaka -0.33 [-0.60 , -0.07]
FBIRN -0.26 [-0.49 , -0.03]
ASRB_site1 -0.50 [-0.89 , -0.11]
ASRB_site2 -0.68 [-0.99 , -0.36]
ASRB_site3 -0.49 [-1.16 , 0.18]
ASRB_site4 0.04 [-0.44 , 0.52]
ASRB_site5 -0.31 [-0.71 , 0.09]
dublin -0.33 [-0.66 , -0.01]
CIAM -0.19 [-0.88 , 0.50]
CAMH -0.23 [-0.48 , 0.02]
BWH_FE -0.78 [-1.46 , -0.10]
BWH_chronic 0.03 [-0.44 , 0.50]
UPENN_12 0.34 [-0.40 , 1.07]
UPENN_64continuous -0.10 [-0.53 , 0.34]
UMCU -0.11 [-0.38 , 0.16]
EDINBURGH -0.68 [-1.19 , -0.17]
Galway -0.72 [-1.10 , -0.35]
Rome -0.27 [-0.54 , 0.00]
HUBIN -0.41 [-0.85 , 0.03]
TOP -0.03 [-0.28 , 0.22]
KaSP 0.02 [-0.53 , 0.57]
MPRC_1 -0.25 [-0.63 , 0.13]
MPRC_2 -0.49 [-0.81 , -0.18]
MPRC_3 -0.76 [-1.02 , -0.51]
Huilong_siemens -0.44 [-0.81 , -0.08]
Huilong_GE2 -0.13 [-0.49 , 0.23]
SYD -0.02 [-0.38 , 0.34]
Prague -0.30 [-0.64 , 0.04]
Olin_12_direction -0.12 [-0.44 , 0.21]
Olin_33_direction -0.24 [-0.57 , 0.08]
RE Model -0.30 [-0.39 , -0.22]
-1.50 -0.50 0.50 1.50
Observed Outcome

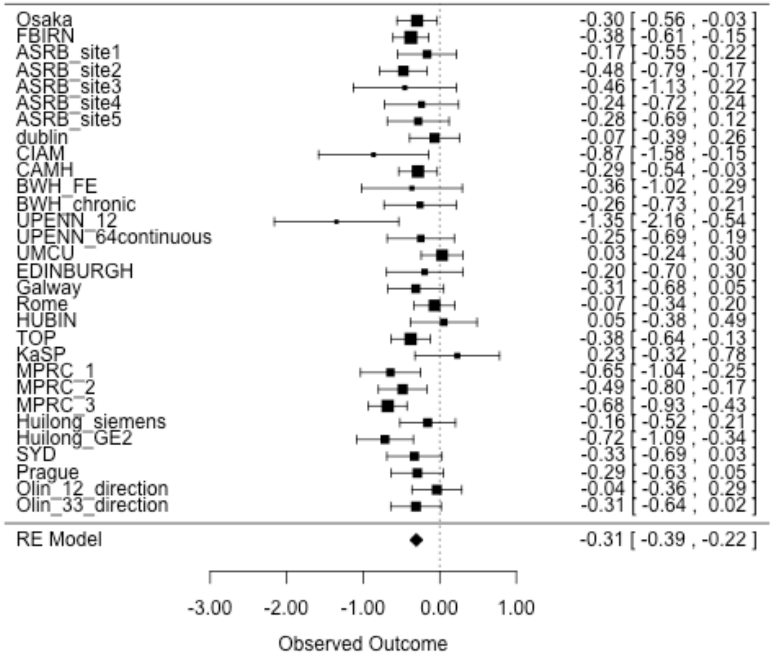
Osaka -0.30 [-0.56 , -0.03]
FBIRN -0.38 [-0.61 , -0.15]
ASRB_site1 -0.17 [-0.55 , 0.22]
ASRB_site2 -0.48 [-0.79 , -0.17]
ASRB_site3 -0.46 [-1.13 , 0.22]
ASRB_site4 -0.24 [-0.72 , 0.24]
ASRB_site5 -0.28 [-0.69 , 0.12]
dublin -0.07 [-0.39 , 0.26]
CIAM -0.87 [-1.58 , -0.15]
CAMH -0.29 [-0.54 , -0.03]
BWH_FE -0.36 [-1.02 , 0.29]
BWH_chronic -0.26 [-0.73 , 0.21]
UPENN_12 -1.35 [-2.16 , -0.54]
UPENN_64continuous -0.25 [-0.69 , 0.19]
UMCU 0.03 [-0.24 , 0.30]
EDINBURGH -0.20 [-0.70 , 0.30]
Galway -0.31 [-0.68 , 0.05]
Rome -0.07 [-0.34 , 0.20]
HUBIN 0.05 [-0.38 , 0.49]
TOP -0.38 [-0.64 , -0.13]
KaSP 0.23 [-0.32 , 0.78]
MPRC_1 -0.65 [-1.04 , -0.25]
MPRC_2 -0.49 [-0.80 , -0.17]
MPRC_3 -0.68 [-0.93 , -0.43]
Huilong_siemens -0.16 [-0.52 , 0.21]
Huilong_GE2 -0.72 [-1.09 , -0.34]
SYD -0.33 [-0.69 , 0.03]
Prague -0.29 [-0.63 , 0.05]
Olin_12_direction -0.04 [-0.36 , 0.29]
Olin_33_direction -0.31 [-0.64 , 0.02]
RE Model -0.31 [-0.39 , -0.22]
-3.00 -2.00 -1.00 0.00 1.00
Observed Outcome

FX

FXST

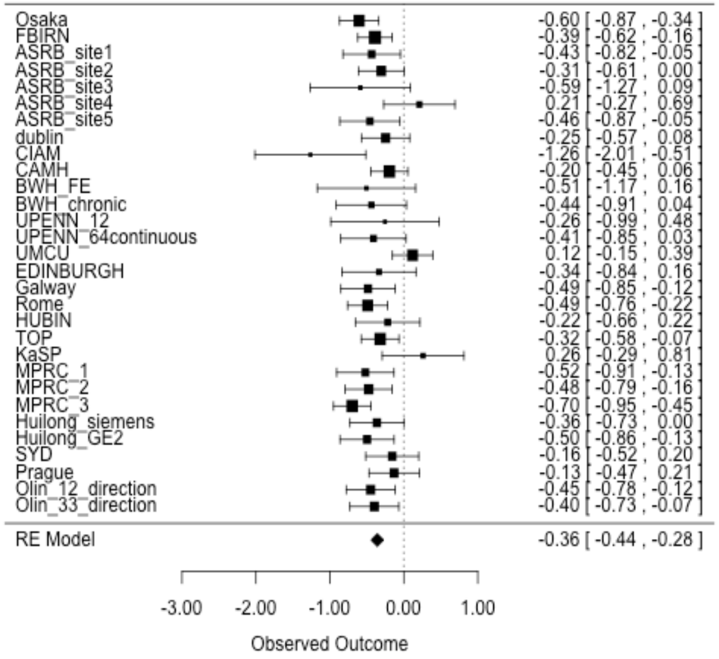
Osaka -0.60 [-0.87 , -0.34]
FBIRN -0.39 [-0.62 , -0.16]
ASRB_site1 -0.43 [-0.82 , -0.05]
ASRB_site2 -0.31 [-0.61 , 0.00]
ASRB_site3 -0.59 [-1.27 , 0.09]
ASRB_site4 0.21 [-0.27 , 0.69]
ASRB_site5 -0.46 [-0.87 , -0.05]
dublin -0.25 [-0.57 , 0.08]
CIAM -1.26 [-2.01 , -0.51]
CAMH -0.20 [-0.45 , 0.06]
BWH_FE -0.51 [-1.17 , 0.16]
BWH_chronic -0.44 [-0.91 , 0.04]
UPENN_12 -0.26 [-0.99 , 0.48]
UPENN_64continuous -0.41 [-0.85 , 0.03]
UMCU 0.12 [-0.15 , 0.39]
EDINBURGH -0.34 [-0.84 , 0.16]
Galway -0.49 [-0.85 , -0.12]
Rome -0.49 [-0.76 , -0.22]
HUBIN -0.22 [-0.66 , 0.22]
TOP -0.32 [-0.58 , -0.07]
KaSP 0.26 [-0.29 , 0.81]
MPRC_1 -0.52 [-0.91 , -0.13]
MPRC_2 -0.48 [-0.79 , -0.16]
MPRC_3 -0.70 [-0.95 , -0.45]
Huilong_siemens -0.36 [-0.73 , 0.00]
Huilong_GE2 -0.50 [-0.86 , -0.13]
SYD -0.16 [-0.52 , 0.20]
Prague -0.13 [-0.47 , 0.21]
Olin_12_direction -0.45 [-0.78 , -0.12]
Olin_33_direction -0.40 [-0.73 , -0.07]
RE Model -0.36 [-0.44 , -0.28]
-3.00 -2.00 -1.00 0.00 1.00
Observed Outcome

GCC

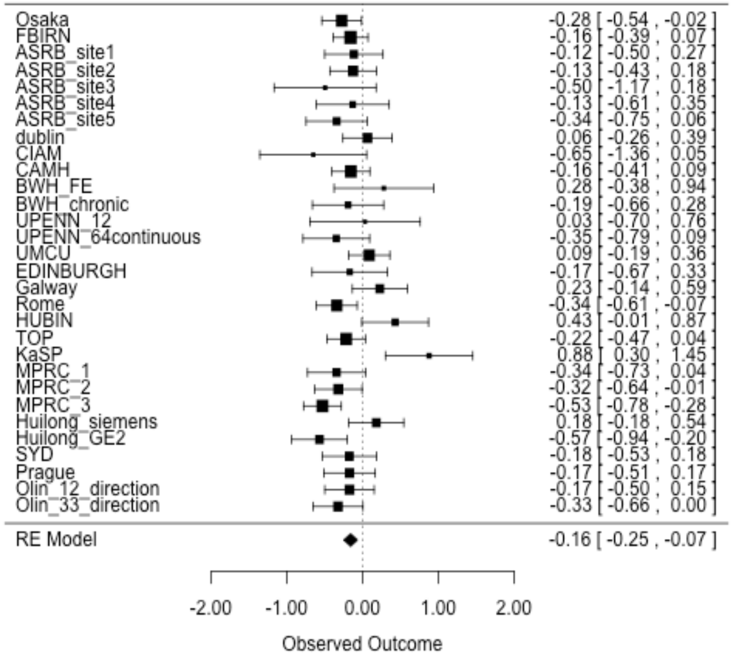
Osaka -0.28 [-0.54 , -0.02]
FBIRN -0.16 [-0.39 , 0.07]
ASRB_site1 -0.12 [-0.50 , 0.27]
ASRB_site2 -0.13 [-0.43 , 0.18]
ASRB_site3 -0.50 [-1.17 , 0.18]
ASRB_site4 -0.13 [-0.61 , 0.35]
ASRB_site5 -0.34 [-0.75 , 0.06]
dublin 0.06 [-0.26 , 0.39]
CIAM -0.65 [-1.36 , 0.05]
CAMH -0.16 [-0.41 , 0.09]
BWH_FE 0.28 [-0.38 , 0.94]
BWH_chronic -0.19 [-0.66 , 0.28]
UPENN_12 0.03 [-0.70 , 0.76]
UPENN_64continuous -0.35 [-0.79 , 0.09]
UMCU 0.09 [-0.19 , 0.36]
EDINBURGH -0.17 [-0.67 , 0.33]
Galway 0.23 [-0.14 , 0.59]
Rome -0.34 [-0.61 , -0.07]
HUBIN 0.43 [-0.01 , 0.87]
TOP -0.22 [-0.47 , 0.04]
KaSP 0.88 [0.30 , 1.45]
MPRC_1 -0.34 [-0.73 , 0.04]
MPRC_2 -0.32 [-0.64 , -0.01]
MPRC_3 -0.53 [-0.78 , -0.28]
Huilong_siemens 0.18 [-0.18 , 0.54]
Huilong_GE2 -0.57 [-0.94 , -0.20]
SYD -0.18 [-0.53 , 0.18]
Prague -0.17 [-0.51 , 0.17]
Olin_12_direction -0.17 [-0.50 , 0.15]
Olin_33_direction -0.33 [-0.66 , 0.00]
RE Model -0.16 [-0.25 , -0.07]
-2.00 -1.00 0.00 1.00 2.00
Observed Outcome

IC

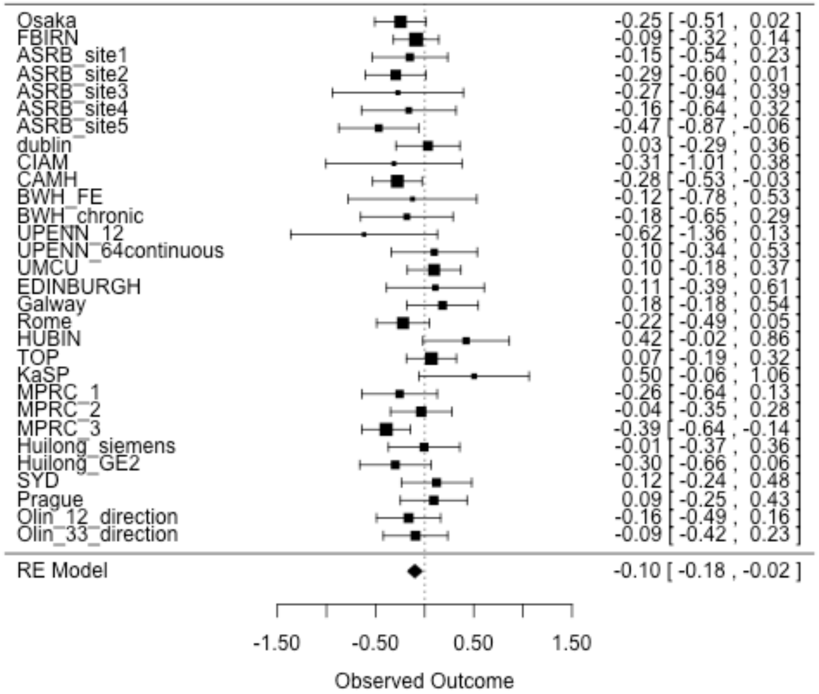
Osaka -0.25 [-0.51 , 0.02]
FBIRN -0.09 [-0.32 , 0.14]
ASRB_site1 -0.15 [-0.54 , 0.23]
ASRB_site2 -0.29 [-0.60 , 0.01]
ASRB_site3 -0.27 [-0.94 , 0.39]
ASRB_site4 -0.16 [-0.64 , 0.32]
ASRB_site5 -0.47 [-0.87 , -0.06]
dublin 0.03 [-0.29 , 0.36]
CIAM -0.31 [-1.01 , 0.38]
CAMH -0.28 [-0.53 , -0.03]
BWH_FE -0.12 [-0.78 , 0.53]
BWH_chronic -0.18 [-0.65 , 0.29]
UPENN_12 -0.62 [-1.36 , 0.13]
UPENN_64continuous 0.10 [-0.34 , 0.53]
UMCU 0.10 [-0.18 , 0.37]
EDINBURGH 0.11 [-0.39 , 0.61]
Galway 0.18 [-0.18 , 0.54]
Rome -0.22 [-0.49 , 0.05]
HUBIN 0.42 [-0.02 , 0.86]
TOP 0.07 [-0.19 , 0.32]
KaSP 0.50 [-0.06 , 1.06]
MPRC_1 -0.26 [-0.64 , 0.13]
MPRC_2 -0.04 [-0.35 , 0.28]
MPRC_3 -0.39 [-0.64 , -0.14]
Huilong_siemens -0.01 [-0.37 , 0.36]
Huilong_GE2 -0.30 [-0.66 , 0.06]
SYD 0.12 [-0.24 , 0.48]
Prague 0.09 [-0.25 , 0.43]
Olin_12_direction -0.16 [-0.49 , 0.16]
Olin_33_direction -0.09 [-0.42 , 0.23]
RE Model -0.10 [-0.18 , -0.02]
-1.50 -0.50 0.50 1.50
Observed Outcome

IFO

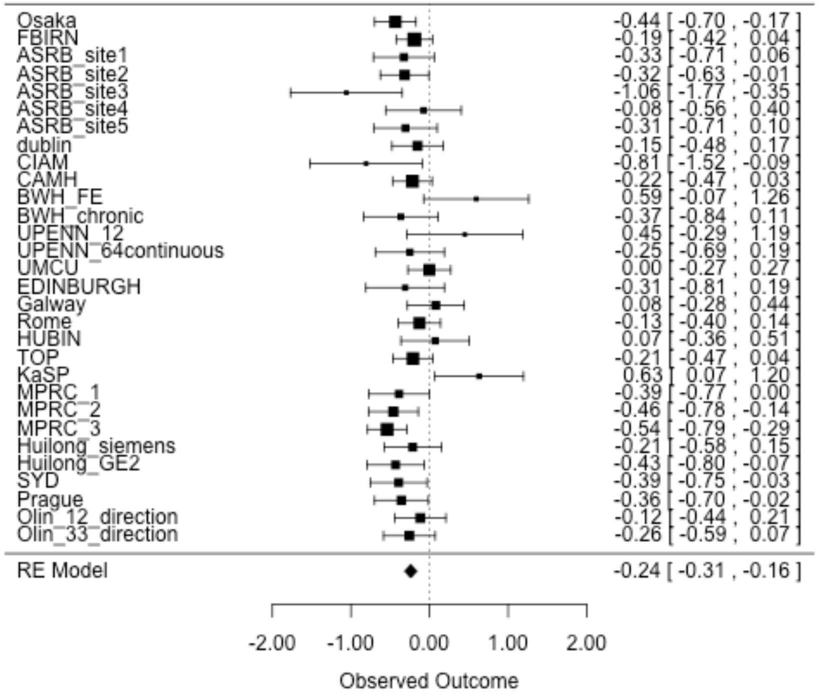
Osaka -0.44 [-0.70 , -0.17]
FBIRN -0.19 [-0.42 , 0.04]
ASRB_site1 -0.33 [-0.71 , 0.06]
ASRB_site2 -0.32 [-0.63 , -0.01]
ASRB_site3 -1.06 [-1.77 , -0.35]
ASRB_site4 -0.08 [-0.56 , 0.40]
ASRB_site5 -0.31 [-0.71 , 0.10]
dublin -0.15 [-0.48 , 0.17]
CIAM -0.81 [-1.52 , -0.09]
CAMH -0.22 [-0.47 , 0.03]
BWH_FE 0.59 [-0.07 , 1.26]
BWH_chronic -0.37 [-0.84 , 0.11]
UPENN_12 0.45 [-0.29 , 1.19]
UPENN_64continuous -0.25 [-0.69 , 0.19]
UMCU 0.00 [-0.27 , 0.27]
EDINBURGH -0.31 [-0.81 , 0.19]
Galway 0.08 [-0.28 , 0.44]
Rome -0.13 [-0.40 , 0.14]
HUBIN 0.07 [-0.36 , 0.51]
TOP -0.21 [-0.47 , 0.04]
KaSP 0.63 [0.07 , 1.20]
MPRC_1 -0.39 [-0.77 , 0.00]
MPRC_2 -0.46 [-0.78 , -0.14]
MPRC_3 -0.54 [-0.79 , -0.29]
Huilong_siemens -0.21 [-0.58 , 0.15]
Huilong_GE2 -0.43 [-0.80 , -0.07]
SYD -0.39 [-0.75 , -0.03]
Prague -0.36 [-0.70 , -0.02]
Olin_12_direction -0.12 [-0.44 , 0.21]
Olin_33_direction -0.26 [-0.59 , 0.07]
RE Model -0.24 [-0.31 , -0.16]
-2.00 -1.00 0.00 1.00 2.00
Observed Outcome

PCR

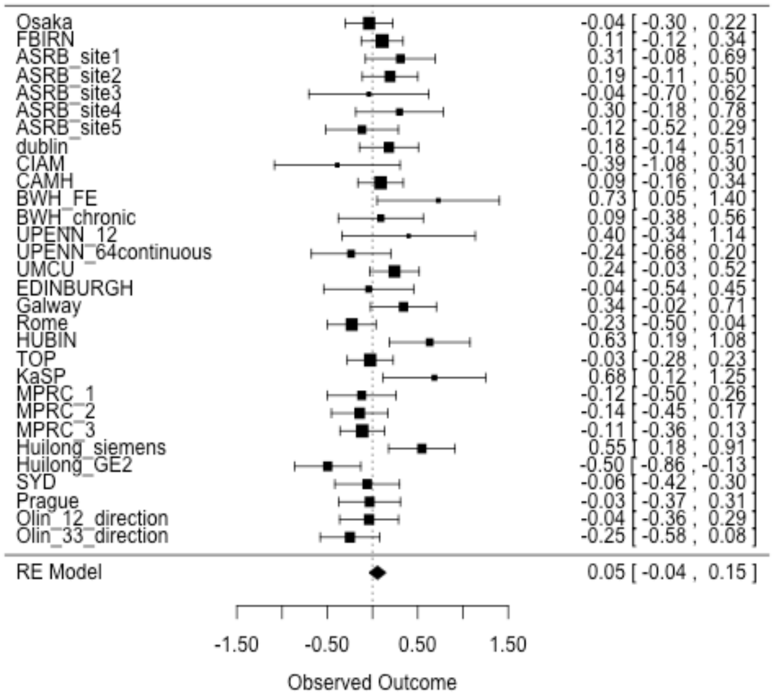

Osaka -0.04 [-0.30 , 0.22]
FBIRN 0.11 [-0.12 , 0.34]
ASRB_site1 0.31 [-0.08 , 0.69]
ASRB_site2 0.19 [-0.11 , 0.50]
ASRB_site3 -0.04 [-0.70 , 0.62]
ASRB_site4 0.30 [-0.18 , 0.78]
ASRB_site5 -0.12 [-0.52 , 0.29]
dublin 0.18 [-0.14 , 0.51]
CIAM -0.39 [-1.08 , 0.30]
CAMH 0.09 [-0.16 , 0.34]
BWH_FE 0.73 [0.05 , 1.40]
BWH_chronic 0.09 [-0.38 , 0.56]
UPENN_12 0.40 [-0.34 , 1.14]
UPENN_64continuous -0.24 [-0.68 , 0.20]
UMCU 0.24 [-0.03 , 0.52]
EDINBURGH -0.04 [-0.54 , 0.45]
Galway 0.34 [-0.02 , 0.71]
Rome -0.23 [-0.50 , 0.04]
HUBIN 0.63 [0.19 , 1.08]
TOP -0.03 [-0.28 , 0.23]
KaSP 0.68 [0.12 , 1.25]
MPRC_1 -0.12 [-0.50 , 0.26]
MPRC_2 -0.14 [-0.45 , 0.17]
MPRC_3 -0.11 [-0.36 , 0.13]
Huilong_siemens 0.55 [0.18 , 0.91]
Huilong_GE2 -0.50 [-0.86 , -0.13]
SYD -0.06 [-0.42 , 0.30]
Prague -0.03 [-0.37 , 0.31]
Olin_12_direction -0.04 [-0.36 , 0.29]
Olin_33_direction -0.25 [-0.58 , 0.08]
RE Model 0.05 [-0.04 , 0.15]
-1.50 -0.50 0.50 1.50
Observed Outcome

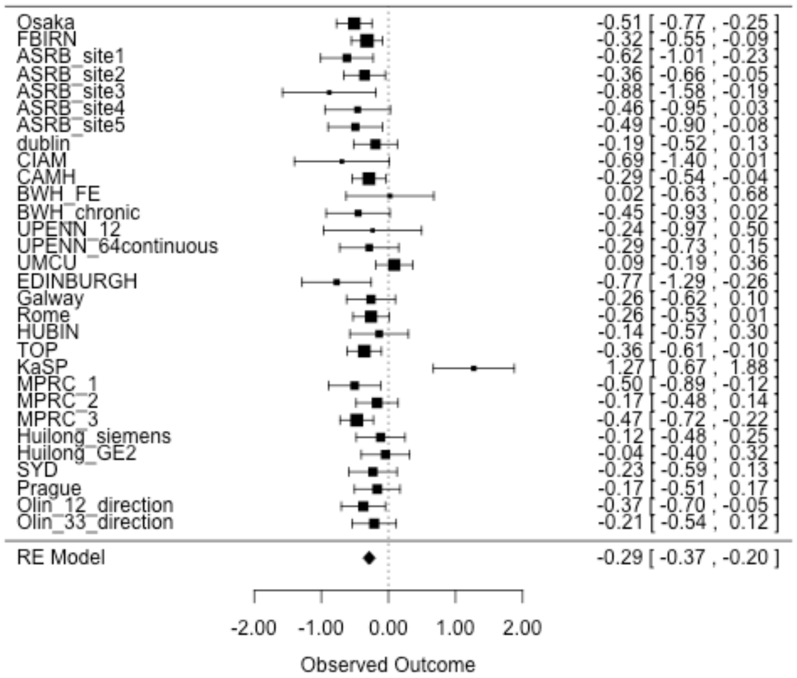

Osaka -0.51 [-0.77 , -0.25]
FBIRN -0.32 [-0.55 , -0.09]
ASRB_site1 -0.62 [-1.01 , -0.23]
ASRB_site2 -0.36 [-0.66 , -0.05]
ASRB_site3 -0.88 [-1.58 , -0.19]
ASRB_site4 -0.46 [-0.95 , 0.03]
ASRB_site5 -0.49 [-0.90 , -0.08]
dublin -0.19 [-0.52 , 0.13]
CIAM -0.69 [-1.40 , 0.01]
CAMH -0.29 [-0.54 , -0.04]
BWH_FE 0.02 [-0.63 , 0.68]
BWH_chronic -0.45 [-0.93 , 0.02]
UPENN_12 -0.24 [-0.97 , 0.50]
UPENN_64continuous -0.29 [-0.73 , 0.15]
UMCU 0.09 [-0.19 , 0.36]
EDINBURGH -0.77 [-1.29 , -0.26]
Galway -0.26 [-0.62 , 0.10]
Rome -0.26 [-0.53 , 0.01]
HUBIN -0.14 [-0.57 , 0.30]
TOP -0.36 [-0.61 , -0.10]
KaSP 1.27 [0.67 , 1.88]
MPRC_1 -0.50 [-0.89 , -0.12]
MPRC_2 -0.17 [-0.48 , 0.14]
MPRC_3 -0.47 [-0.72 , -0.22]
Huilong_siemens -0.12 [-0.48 , 0.25]
Huilong_GE2 -0.04 [-0.40 , 0.32]
SYD -0.23 [-0.59 , 0.13]
Prague -0.17 [-0.51 , 0.17]
Olin_12_direction -0.37 [-0.70 , -0.05]
Olin_33_direction -0.21 [-0.54 , 0.12]
RE Model -0.29 [-0.37 , -0.20]
-2.00 -1.00 0.00 1.00 2.00
Observed Outcome

PLIC **PTR**

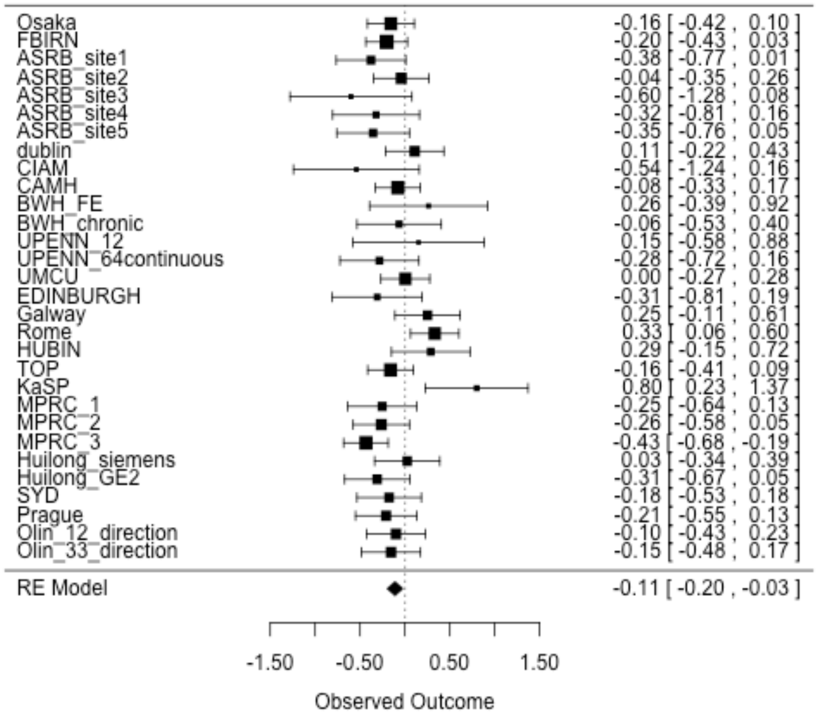
Osaka -0.16 [-0.42 , 0.10]
FBIRN -0.20 [-0.43 , 0.03]
ASRB_site1 -0.38 [-0.77 , 0.01]
ASRB_site2 -0.04 [-0.35 , 0.26]
ASRB_site3 -0.60 [-1.28 , 0.08]
ASRB_site4 -0.32 [-0.81 , 0.16]
ASRB_site5 -0.35 [-0.76 , 0.05]
dublin 0.11 [-0.22 , 0.43]
CIAM -0.54 [-1.24 , 0.16]
CAMH -0.08 [-0.33 , 0.17]
BWH_FE 0.26 [-0.39 , 0.92]
BWH_chronic -0.06 [-0.53 , 0.40]
UPENN_12 0.15 [-0.58 , 0.88]
UPENN_64continuous -0.28 [-0.72 , 0.16]
UMCU 0.00 [-0.27 , 0.28]
EDINBURGH -0.31 [-0.81 , 0.19]
Galway 0.25 [-0.11 , 0.61]
Rome 0.33 [0.06 , 0.60]
HUBIN 0.29 [-0.15 , 0.72]
TOP -0.16 [-0.41 , 0.09]
KaSP 0.80 [0.23 , 1.37]
MPRC_1 -0.25 [-0.64 , 0.13]
MPRC_2 -0.26 [-0.58 , 0.05]
MPRC_3 -0.43 [-0.68 , -0.19]
Huilong_siemens 0.03 [-0.34 , 0.39]
Huilong_GE2 -0.31 [-0.67 , 0.05]
SYD -0.18 [-0.53 , 0.18]
Prague -0.21 [-0.55 , 0.13]
Olin_12_direction -0.10 [-0.43 , 0.23]
Olin_33_direction -0.15 [-0.48 , 0.17]
RE Model -0.11 [-0.20 , -0.03]
-1.50
-0.50
0.50
1.50
Observed Outcome

RLIC

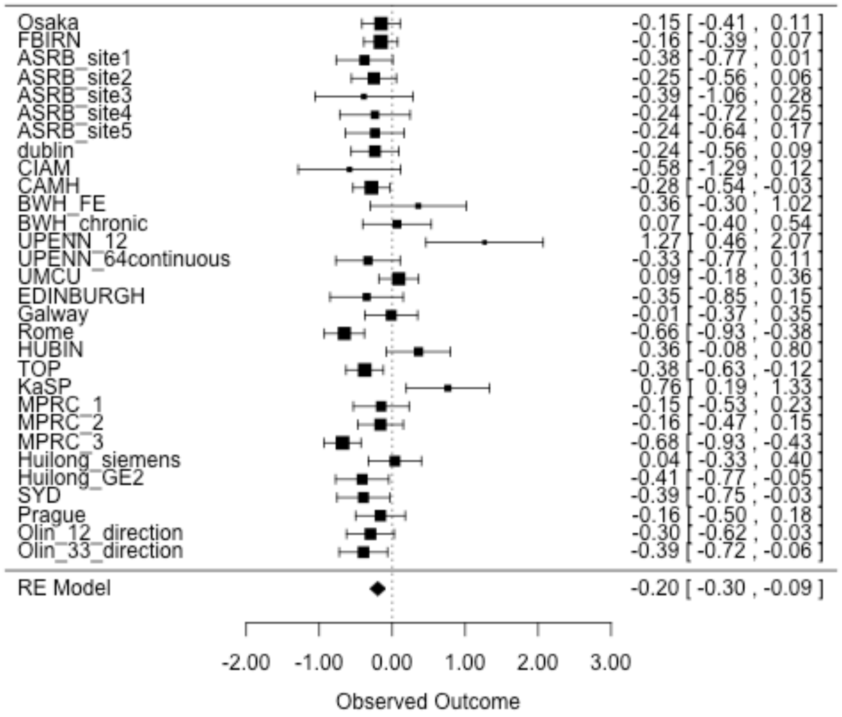
Osaka -0.15 [-0.41 , 0.11]
FBIRN -0.16 [-0.39 , 0.07]
ASRB_site1 -0.38 [-0.77 , 0.01]
ASRB_site2 -0.25 [-0.56 , 0.06]
ASRB_site3 -0.39 [-1.06 , 0.28]
ASRB_site4 -0.24 [-0.72 , 0.25]
ASRB_site5 -0.24 [-0.64 , 0.17]
dublin -0.24 [-0.56 , 0.09]
CIAM -0.58 [-1.29 , 0.12]
CAMH -0.28 [-0.54 , -0.03]
BWH_FE 0.36 [-0.30 , 1.02]
BWH_chronic 0.07 [-0.40 , 0.54]
UPENN_12 1.27 [0.46 , 2.07]
UPENN_64continuous -0.33 [-0.77 , 0.11]
UMCU 0.09 [-0.18 , 0.36]
EDINBURGH -0.35 [-0.85 , 0.15]
Galway -0.01 [-0.37 , 0.35]
Rome -0.66 [-0.93 , -0.38]
HUBIN 0.36 [-0.08 , 0.80]
TOP -0.38 [-0.63 , -0.12]
KaSP 0.76 [0.19 , 1.33]
MPRC_1 -0.15 [-0.53 , 0.23]
MPRC_2 -0.16 [-0.47 , 0.15]
MPRC_3 -0.68 [-0.93 , -0.43]
Huilong_siemens 0.04 [-0.33 , 0.40]
Huilong_GE2 -0.41 [-0.77 , -0.05]
SYD -0.39 [-0.75 , -0.03]
Prague -0.16 [-0.50 , 0.18]
Olin_12_direction -0.30 [-0.62 , 0.03]
Olin_33_direction -0.39 [-0.72 , -0.06]
RE Model -0.20 [-0.30 , -0.09]
-2.00
-1.00
0.00
1.00
2.00
3.00
Observed Outcome

SCC

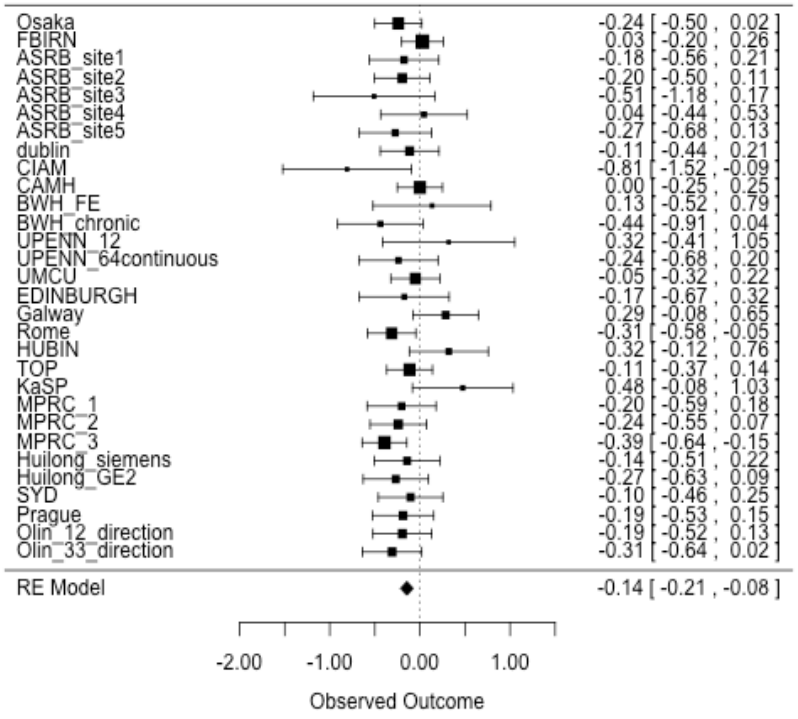

Osaka -0.24 [-0.50 , 0.02]
FBIRN 0.03 [-0.20 , 0.26]
ASRB_site1 -0.18 [-0.56 , 0.21]
ASRB_site2 -0.20 [-0.50 , 0.11]
ASRB_site3 -0.51 [-1.18 , 0.17]
ASRB_site4 0.04 [-0.44 , 0.53]
ASRB_site5 -0.27 [-0.68 , 0.13]
dublin -0.11 [-0.44 , 0.21]
CIAM -0.81 [-1.52 , -0.09]
CAMH 0.00 [-0.25 , 0.25]
BWH_FE 0.13 [-0.52 , 0.79]
BWH_chronic -0.44 [-0.91 , 0.04]
UPENN_12 0.32 [-0.41 , 1.05]
UPENN_64continuous -0.24 [-0.68 , 0.20]
UMCU -0.05 [-0.32 , 0.22]
EDINBURGH -0.17 [-0.67 , 0.32]
Galway 0.29 [-0.08 , 0.65]
Rome -0.31 [-0.58 , -0.05]
HUBIN 0.32 [-0.12 , 0.76]
TOP -0.11 [-0.37 , 0.14]
KaSP 0.48 [-0.08 , 1.03]
MPRC_1 -0.20 [-0.59 , 0.18]
MPRC_2 -0.24 [-0.55 , 0.07]
MPRC_3 -0.39 [-0.64 , -0.15]
Huilong_siemens -0.14 [-0.51 , 0.22]
Huilong_GE2 -0.27 [-0.63 , 0.09]
SYD -0.10 [-0.46 , 0.25]
Prague -0.19 [-0.53 , 0.15]
Olin_12_direction -0.19 [-0.52 , 0.13]
Olin_33_direction -0.31 [-0.64 , 0.02]
RE Model -0.14 [-0.21 , -0.08]
-2.00 -1.00 0.00 1.00
Observed Outcome

SCR

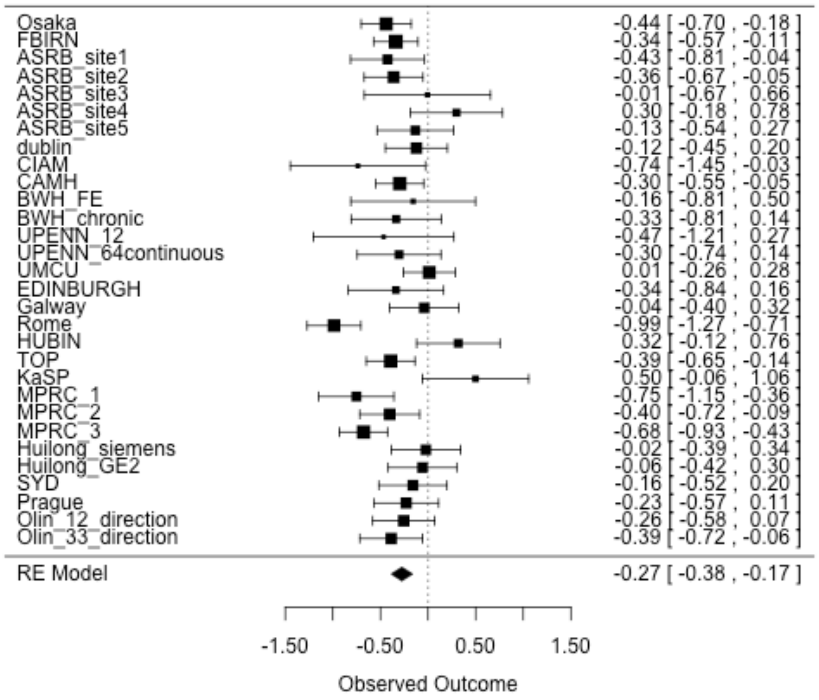

Osaka -0.44 [-0.70 , -0.18]
FBIRN -0.34 [-0.57 , -0.11]
ASRB_site1 -0.43 [-0.81 , -0.04]
ASRB_site2 -0.36 [-0.67 , -0.05]
ASRB_site3 -0.01 [-0.67 , 0.66]
ASRB_site4 0.30 [-0.18 , 0.78]
ASRB_site5 -0.13 [-0.54 , 0.27]
dublin -0.12 [-0.45 , 0.20]
CIAM -0.74 [-1.45 , -0.03]
CAMH -0.30 [-0.55 , -0.05]
BWH_FE -0.16 [-0.81 , 0.50]
BWH_chronic -0.33 [-0.81 , 0.14]
UPENN_12 -0.47 [-1.21 , 0.27]
UPENN_64continuous -0.30 [-0.74 , 0.14]
UMCU 0.01 [-0.26 , 0.28]
EDINBURGH -0.34 [-0.84 , 0.16]
Galway -0.04 [-0.40 , 0.32]
Rome -0.99 [-1.27 , -0.71]
HUBIN 0.32 [-0.12 , 0.76]
TOP -0.39 [-0.65 , -0.14]
KaSP 0.50 [-0.06 , 1.06]
MPRC_1 -0.75 [-1.15 , -0.36]
MPRC_2 -0.40 [-0.72 , -0.09]
MPRC_3 -0.68 [-0.93 , -0.43]
Huilong_siemens -0.02 [-0.39 , 0.34]
Huilong_GE2 -0.06 [-0.42 , 0.30]
SYD -0.16 [-0.52 , 0.20]
Prague -0.23 [-0.57 , 0.11]
Olin_12_direction -0.26 [-0.58 , 0.07]
Olin_33_direction -0.39 [-0.72 , -0.06]
RE Model -0.27 [-0.38 , -0.17]
-1.50 -0.50 0.50 1.50
Observed Outcome

SFO

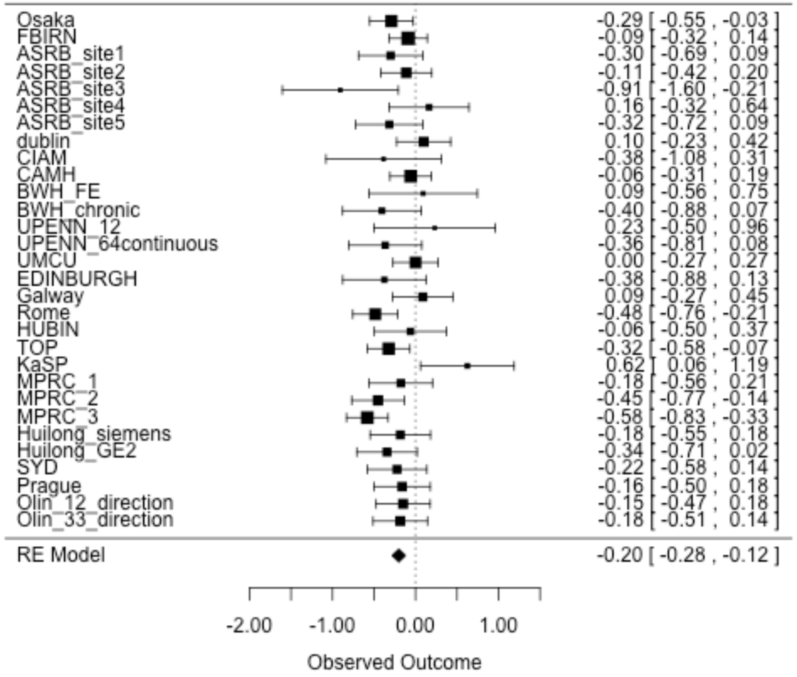
Osaka -0.29 [-0.55 , -0.03]
FBIRN -0.09 [-0.32 , 0.14]
ASRB_site1 -0.30 [-0.69 , 0.09]
ASRB_site2 -0.11 [-0.42 , 0.20]
ASRB_site3 -0.91 [-1.60 , -0.21]
ASRB_site4 0.16 [-0.32 , 0.64]
ASRB_site5 -0.32 [-0.72 , 0.09]
dublin 0.10 [-0.23 , 0.42]
CIAM -0.38 [-1.08 , 0.31]
CAMH -0.06 [-0.31 , 0.19]
BWH_FE 0.09 [-0.56 , 0.75]
BWH_chronic -0.40 [-0.88 , 0.07]
UPENN_12 0.23 [-0.50 , 0.96]
UPENN_64continuous -0.36 [-0.81 , 0.08]
UMCU 0.00 [-0.27 , 0.27]
EDINBURGH -0.38 [-0.88 , 0.13]
Galway 0.09 [-0.27 , 0.45]
Rome -0.48 [-0.76 , -0.21]
HUBIN -0.06 [-0.50 , 0.37]
TOP -0.32 [-0.58 , -0.07]
KaSP 0.62 [0.06 , 1.19]
MPRC_1 -0.18 [-0.56 , 0.21]
MPRC_2 -0.45 [-0.77 , -0.14]
MPRC_3 -0.58 [-0.83 , -0.33]
Huilong_siemens -0.18 [-0.55 , 0.18]
Huilong_GE2 -0.34 [-0.71 , 0.02]
SYD -0.22 [-0.58 , 0.14]
Prague -0.16 [-0.50 , 0.18]
Olin_12_direction -0.15 [-0.47 , 0.18]
Olin_33_direction -0.18 [-0.51 , 0.14]
RE Model -0.20 [-0.28 , -0.12]
-2.00 -1.00 0.00 1.00
Observed Outcome

SLF

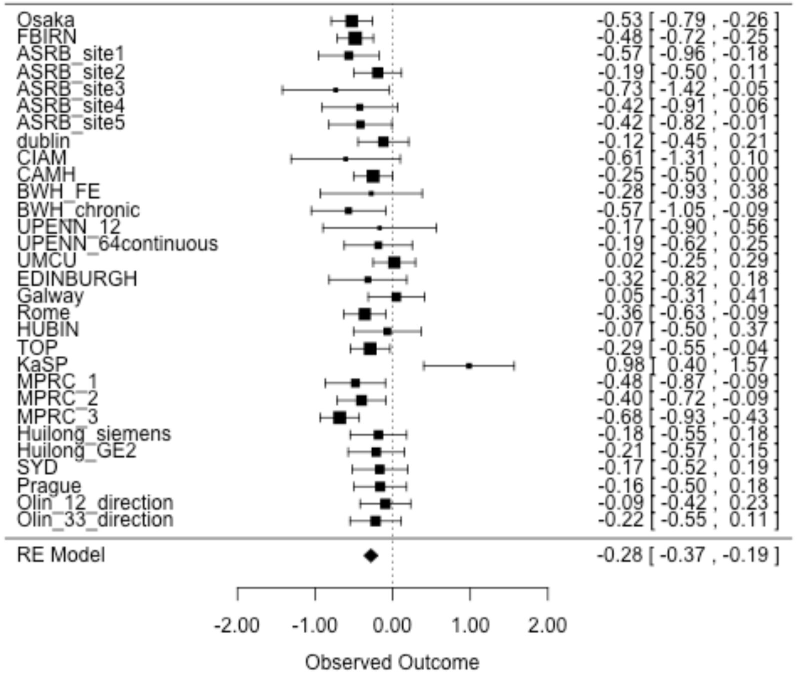
Osaka -0.53 [-0.79 , -0.26]
FBIRN -0.48 [-0.72 , -0.25]
ASRB_site1 -0.57 [-0.96 , -0.18]
ASRB_site2 -0.19 [-0.50 , 0.11]
ASRB_site3 -0.73 [-1.42 , -0.05]
ASRB_site4 -0.42 [-0.91 , 0.06]
ASRB_site5 -0.42 [-0.82 , -0.01]
dublin -0.12 [-0.45 , 0.21]
CIAM -0.61 [-1.31 , 0.10]
CAMH -0.25 [-0.50 , 0.00]
BWH_FE -0.28 [-0.93 , 0.38]
BWH_chronic -0.57 [-1.05 , -0.09]
UPENN_12 -0.17 [-0.90 , 0.56]
UPENN_64continuous -0.19 [-0.62 , 0.25]
UMCU 0.02 [-0.25 , 0.29]
EDINBURGH -0.32 [-0.82 , 0.18]
Galway 0.05 [-0.31 , 0.41]
Rome -0.36 [-0.63 , -0.09]
HUBIN -0.07 [-0.50 , 0.37]
TOP -0.29 [-0.55 , -0.04]
KaSP 0.98 [0.40 , 1.57]
MPRC_1 -0.48 [-0.87 , -0.09]
MPRC_2 -0.40 [-0.72 , -0.09]
MPRC_3 -0.68 [-0.93 , -0.43]
Huilong_siemens -0.18 [-0.55 , 0.18]
Huilong_GE2 -0.21 [-0.57 , 0.15]
SYD -0.17 [-0.52 , 0.19]
Prague -0.16 [-0.50 , 0.18]
Olin_12_direction -0.09 [-0.42 , 0.23]
Olin_33_direction -0.22 [-0.55 , 0.11]
RE Model -0.28 [-0.37 , -0.19]
-2.00 -1.00 0.00 1.00 2.00
Observed Outcome

SS

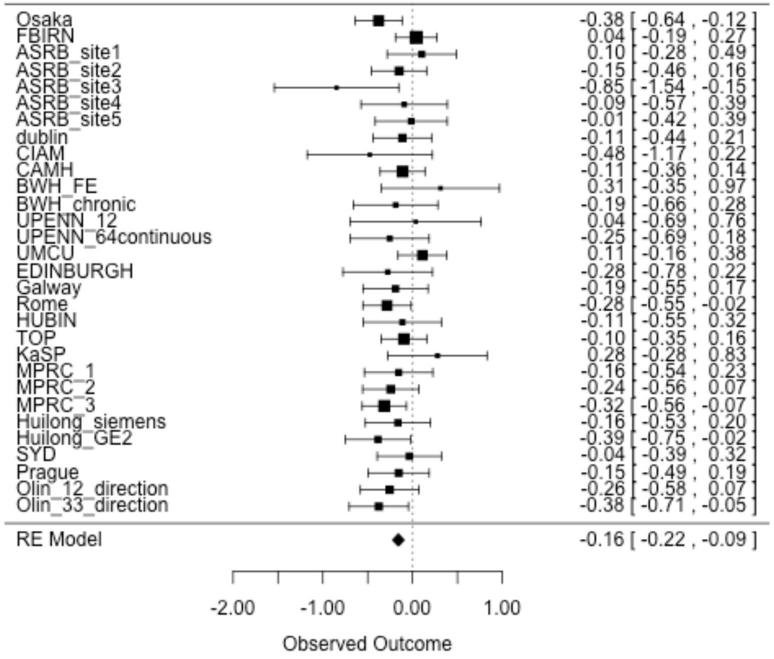
Osaka -0.38 [-0.64 , -0.12]
FBIRN 0.04 [-0.19 , 0.27]
ASRB_site1 0.10 [-0.28 , 0.49]
ASRB_site2 -0.15 [-0.46 , 0.16]
ASRB_site3 -0.85 [-1.54 , -0.15]
ASRB_site4 -0.09 [-0.57 , 0.39]
ASRB_site5 -0.01 [-0.42 , 0.39]
dublin -0.11 [-0.44 , 0.21]
CIAM -0.48 [-1.17 , 0.22]
CAMH -0.11 [-0.36 , 0.14]
BWH_FE 0.31 [-0.35 , 0.97]
BWH_chronic -0.19 [-0.66 , 0.28]
UPENN_12 0.04 [-0.69 , 0.76]
UPENN_64continuous -0.25 [-0.69 , 0.18]
UMCU 0.11 [-0.16 , 0.38]
EDINBURGH -0.28 [-0.78 , 0.22]
Galway -0.19 [-0.55 , 0.17]
Rome -0.28 [-0.55 , -0.02]
HUBIN -0.11 [-0.55 , 0.32]
TOP -0.10 [-0.35 , 0.16]
KaSP 0.28 [-0.28 , 0.83]
MPRC_1 -0.16 [-0.54 , 0.23]
MPRC_2 -0.24 [-0.56 , 0.07]
MPRC_3 -0.32 [-0.56 , -0.07]
Huilong_siemens -0.16 [-0.53 , 0.20]
Huilong_GE2 -0.39 [-0.75 , -0.02]
SYD -0.04 [-0.39 , 0.32]
Prague -0.15 [-0.49 , 0.19]
Olin_12_direction -0.26 [-0.58 , 0.07]
Olin_33_direction -0.38 [-0.71 , -0.05]
RE Model -0.16 [-0.22 , -0.09]
-2.00 -1.00 0.00 1.00
Observed Outcome

UNC

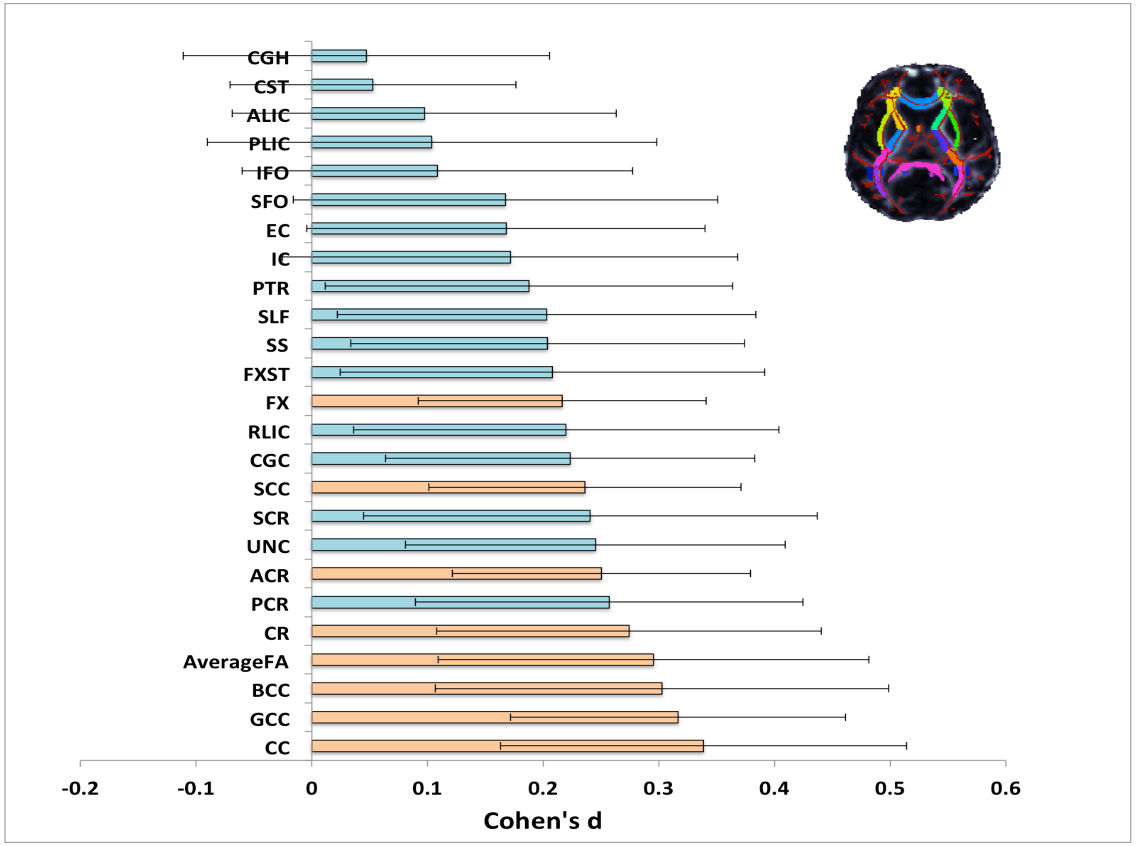

Supplementary figure 6. Cohen's *d* effect sizes, after meta-analysis, for MD differences between schizophrenia patients and healthy controls, including age, sex, age×sex, age and age×sex, as covariates. Error bars represent the 95% confidence interval. Significant regions ($p < 0.05/25 = 0.002$) are indicated with an asterisk (*).

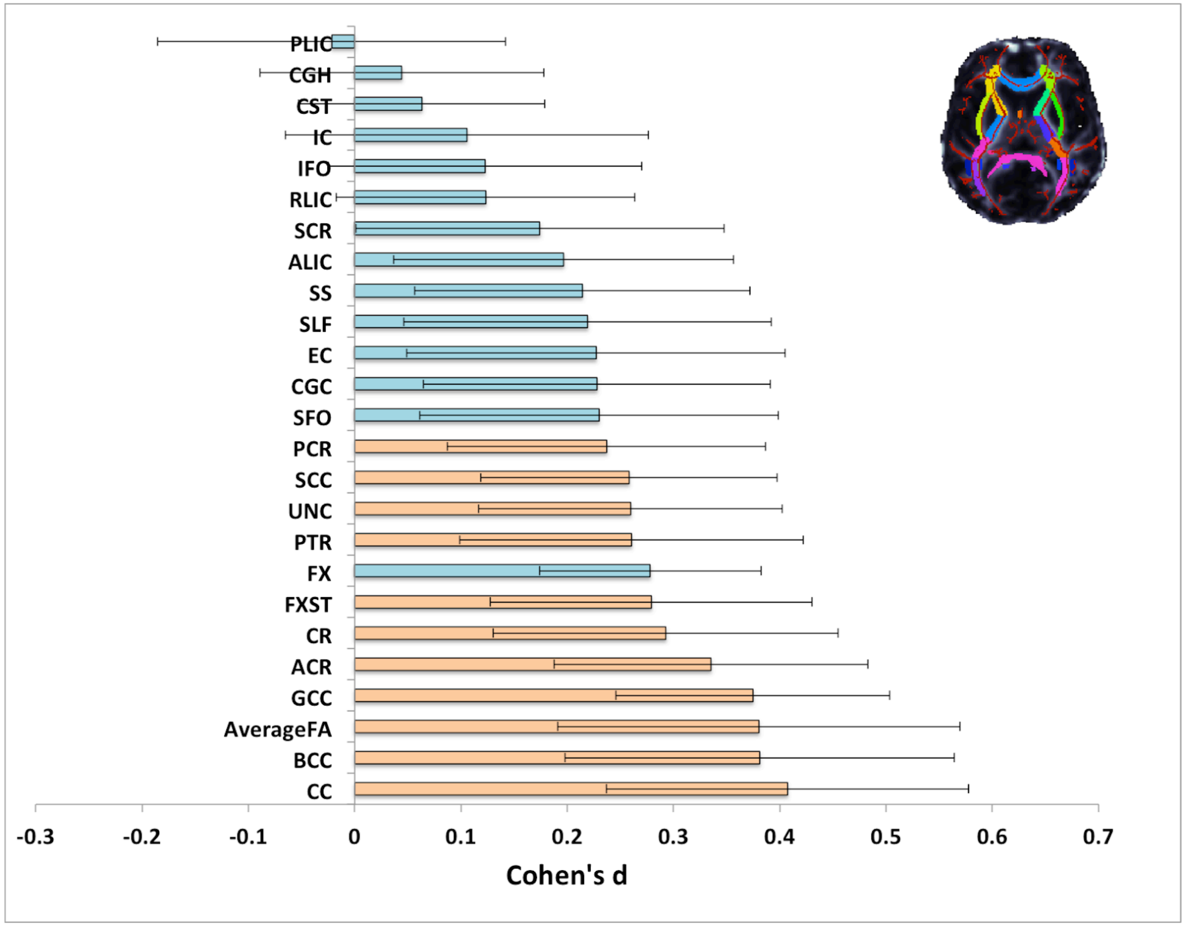

Supplementary figure 7. Cohen's *d* effect sizes, after meta-analysis, for RD differences between schizophrenia patients and healthy controls, including age, sex, age×sex, age and age×sex, as covariates. Error bars represent the 95% confidence interval. Significant regions ($p < 0.05/25 = 0.002$) are indicated with an asterisk (*).

Study Cohort	Scanner	Field strength	Acquisitions	Voxel size and slice thickness	Gradient directions and b-value (mm/s2)	b=0 scans
**ESO study (Prague)**	Siemens TRIO	3T	2	2x 2x2 mm	30 at b=900	2
**Osaka**	GE	3T	1	1.0156x1.0156x33.0 mm	15 at b = 1000	1
**KaSP**	GE	3T	1	0.94 x 0.94 x 2.90 mm	60 at b=1000	10
**HUBIN**	GE	3T	1	0.94 x 0.94 x 2.90 mm	60 at b=1000	10
**TOP**	GE	3T	1	1.875 x 1.875 x 2.5 mm	30 at b=1000	1
**Brain and Mind Research Institute (SYD)**	GE MR750	3T	1	0.9x2.0x2.0 mm	69 at b=1159	8
**Olin(12 direction)**	Siemens	3T	1	1.56x1.56x3 mm	12 at b=1000	1
**Olin(32 direction)**	Siemens	3T	1	1.7x1.7x3 mm	32 at b = 1000	1
**CAMH**	GE	1.5T	3	Voxel size=2x2mm Thickness	23 at b=1000	2
**BWH_FE (CIDAR)**	GE Echospeed	3T	1	1.7x1.7x1.7 mm	51 at b=900	8
**BWH_chronic (CIDAR)**	GE Echospeed	3T	1	1.7x1.7x1.7 mm	51 at b=900	8
**Dublin**	Philips Achieva	3T	1	2x2x2.3mm	15 at b=800	1
**Galway**	Siemens Magnetom Symphony	1.5T	1	2.5x2.5x2.5	64 at b=1300	7
**UPENN (12)**	Siemens Trio	3T	1	1.72x1.72x3mm	12 at b=1000	1
**UPENN (64)**	Siemens Trio	3T	1	1.875x1.875x2.0mm	64 at b=1000	1
**MPRC (Study1)**	Siemens Alegra	3T	1	1.7×1.7×4.0	12 at b=1000	8
**MPRC (Study2)**	Siemens Trio	3T	1	1.8×1.8×3.0	30 at b=1000	3

**MPRC (Study3)**	Siemens Trio	3T	1	1.7×1.7×3.0	64 at b=800	5
**UMCU**	Philips Achieva	1.5T	2	2.5x2.5x2.5	32 at b = 1000	8
**Roma_S_Lucia (Rome)**	Siemens Allegra	3T	3	1.8x1.8x1.8 mm3	30 at b=1000	2
**CIAM_SouthAfrica**	Siemens Allegra	3T	2 - one AP, one PA	1.8×1.8×4.0 mm.	30 at b = 1000 and b = 0	2
**Australia (ASRB) site 1**	Siemnes Avanto	1.5 T	1, AP, shimmed EPI	2.4x 2.4x 2.4 mm	64 at b =1000	1
**Australia (ASRB) site 2**	Siemnes Avanto	1.5 T	1 AP, shimmed EPI	2.4x 2.4x 2.4 mm	64 dx, b =1000	1
**Australia (ASRB) site 3**	Siemnes Avanto	1.5 T	1 AP, shimmed EPI	2.4x 2.4x 2.4 mm	64 dx, b =1000	1
**Australia (ASRB) site 4**	Siemnes Avanto	1.5 T	1 AP, shimmed EPI	2.4x 2.4x 2.4 mm	64 dx, b =1000	1
**Australia (ASRB) site 5**	Siemnes Avanto	1.5 T	1 AP, shimmed EPI	2.4x 2.4x 2.4 mm	64 dx, b =1000	1
**FBIRN**	Siemens Trio	3T	1	2x2x2 mm	30 at b=800	5
Huilongguan Siemens	Siemens Trio	3T	1 AP, shimmed EPI	1.8 x1.8 x3.0 mm	64 dx, b =1000 mm/s2	1
Huilongguan GE	GE	3T	1 AP, shimmed EPI	1.0 x1.0 x3.0 mm	32 dx, b =1000 mm/s2	3
Edinburgh	Siemens Magnetom Verio	3T	1	2.2x2.5x2.5mm	56 at b=1000	6

Supplementary table 1. DTI acquisition protocols for each site

Dataset name	N cases/controls	Mean age (cases)	Mean age (controls)	std dev age (cases)	std dev age (controls)	Age range patients	Age range controls	M/F (cases)	M/F (controls)
**ESO study (Prague)**	77/59	31.1	28.9	7.7	7.1	19-53	19-48	34/43	28/31
**Osaka**	71/249	34	31.2	12.1	13.2	18-68	18-66	36/35	138/111
**KaSP**	21/32	30.43	27.47	9.21	5.67	18-58	20-43	14/7	15/17
**HUBIN**	37/45	51.95	54.62	8.21	8.69	37-64	33-69	8/29	31/14
**TOP**	76/275	28.08	31.89	7.83	7.56	18-53	18-46	48/28	161/114
**Brain and Mind Research Institute (SYD)**	51/73	23.34	26.77	4.53	23.69	18-16	18-35	37/14	31/42
**Olin(12 direction)**	57/100	34.5	31	11.82	11.75	18-56	18-77	36/21	53/47
**Olin(33 direction)**	63/83	35.9	36	13.11	13.14	18-64	20-70	47/16	62/21
**CAMH**	113/134	41.77	42.43	16.4	19.2	18-77	18-86	70/43	66/68
**BWH_FE (CIDAR)**	18/18	36.762	36.502	13.891	12.681	18-53	18-57	3/15	8/10
**BWH_chronic (CIDAR)**	37/33	45.66	45.26	9	6.56	27-56	29-53	7/30	6/27
**Dublin**	49/140	43.5	32.24	10.9	11.58	22-61	19-64	35/14	68/72
**Galway**	44/88	34.09	35.93	10.4	10..97	19-58	18-57	33/11	55/33
**Upenn (12)**	15/14	35.53	29.35	8.8	6.23	21-50	21-42	10/5	8/6
**Upenn (64)**	44/37	39.2	32.7	10.02	9.6	22-60	19-58	24/20	18/19
**MPRC (Study1)**	59/48	38.71	36.98	12.25	13.13	21-62	19-61	49/10	33/15
**MPRC (Study2)**	82/76	36.53	42.92	12.29	12.44	18-63	18-64	58/24	31/45
**MPRC (Study3)**	103/168	35.79	38.29	12.7	15.15	18-59	18-77	72/31	74/94

**UMCU**	128/87	26.38	27.06	5.7	8	18-43	18-45	106/22	44/45
**Roma_S_Lucia**	84/148	39.261	48.282	11.588	15.919	18-66	19-78	56/28	76/72
**CIAM_SouthAfrica**	12/25	31.25	25.88	7.65	4.39	20-40	19-34	8/4	12/13
**Australia (ASRB) site 1**	121/33	38.67	40.15	10.98	14.05	20-65	18-63	89/32	16/17
**Australia (ASRB) site 2**	85/79	37.83	41.3	10.43	13.78	20-63	19-65	54/31	41/38
**Australia (ASRB) site 3**	17/18	41.7	43.94	8.72	13.64	27-58	19-62	12/5	9/9
**Australia (ASRB) site 4**	39/29	38.72	37	10.38	13.73	20-59	18-65	28/11	15/14
**Australia (ASRB) site 5**	64/38	40.04	40	10.41	14.89	20-64	18-62	42/22	18/20
**FBIRN**	143/146	38.93	37.05	11.78	10.91	18-62	19-60	109/34	104/42
**Huilongang Siemens**	154/36	29.02	31.75	5.62	6.51	18-40	19-40	84/70	20/16
**Huilongang GE**	90/44	24.25	24.93	6.11	5.64	18-43	18-37	48/42	25/19
**Edinburgh**	28/35	38.04	37.8	9.63	14.8	23-57	20-67	17/11	18/17

Supplementary table 2: ENIGMA-Schizophrenia DTI site demographics

Site	PANSS Pos	PANSS Neg	PANSS Total	SANS Total	SAPS Total	Mean age at onset	Mean duration of illness	Mean CPZ (Woods, 2005)	Number of patients on Typical/Atypical/Both/Neither
**ESO study (Prague)**	19.8	16.2	74.3	n/a	n/a	n/a	5.5 months	306.9	1/65/1/4
**Osaka**	19.2	20.6	84.6	n/a	n/a	22.8	11.2	731.3	3/62/8/7
**KASP**	20.14	17.71	79.67	n/a	n/a	n/a	14 months	n/a	0/9/1/11
**HUBIN**	n/a	n/a	n/a	28.4 +/- 14.2	9 +/- 7.9	23.6 +/- 4.22	28.35 +/- 8.51	348.58 +/- 296.09	11/15/8/3
**TOP**	13.83 +/- 4.88	14.54 +/- 4.96	57.59 +/- 13.29	n/a	n/a	21.27 +/- 6.28	6.85 +/- 6.55	340.75 +/- 310.9	2/50/2/2
**Brain and Mind Research Institute (SYD)**	NA	NA	NA	NA	NA	18.16y	5.48y	339.6	0/46/0/18
**Olin (12 direction)**	NA	NA	NA	NA	NA	21.9(6.1)	9.3(8.0)	NA	NA
**Olin (33 direction)**	NA	NA	NA	NA	NA	NA	NA	NA	Na
**CAMH**	13.8(6.0)	13.2(5.7)	51.8(15.23)	NA	NA	24.9(9.2)	16.8(15.1) year	272.6(291.4)	7/78/8/20
**BWH_FE (CIDAR)**	na	na	86.353 +/- 30.723	21.46 +/- 12.69	22.98 +/- 22.25	22.17+/- 4.93	13.09 +/-12.37 year	416.784 +/- 400.899	NA
**BWH_chronic (CIDAR)**	NA	NA	NA	25.05	26.97	23.52	22.22	430.22	NA
**Dublin**	NA	NA	NA	3.46	2.38	23	19.56	339	
**Galway**	13.76	22.92	22.92	NA	NA	23.72	10.48	482.83	NA
**Upenn (12)**	NA	NA	NA	13.4	14.26	20.07	15.21	379.166	0/4/0/0
**Upenn (64)**	NA	NA	NA	21.29	13.77	21.59	18.07	18.07	4/4/2/0
**MPRC (Study1)**	NA	NA	NA	NA	NA	NA	NA	NA	NA

**MPRC (Study2)**	NA	NA	NA	NA	NA	NA	NA	NA	NA
**MPRC (Study3)**	NA	NA	NA	NA	NA	NA	NA	NA	NA
**UMCU**	15.43	15.64	62.88	n/a	n/a	21.9	3.67	NA	11/102/2/143
**Roma_S_Lucia**	23.233	20.791	92.651	9.702	9.06	25.261	14y	416.942	15/44/23/6
**CIAM_SouthAfrica**	12.6(4.9)	15.2(6.4)	54.2(17.5)	n/a	n/a	21.9(6.1)	9.3(8.0)	NA	4/6/1/1
**Australia (ASRB) site 1**	NA	NA	NA	NA	NA	22.9	15.7	NA	NA
**Australia (ASRB) site 2**	NA	NA	NA	NA	NA	23.3	14.5	NA	NA
**Australia (ASRB) site 3**	NA	NA	NA	NA	NA	23.4	18.2	NA	NA
**Australia (ASRB) site 4**	NA	NA	NA	NA	NA	23	15.8	NA	NA
**Australia (ASRB) site 5**	NA	NA	NA	NA	NA	22.8	17.3	NA	NA
**FBIRN**	15.17	14.63	14.63	20.52	15.79	21.96	17.05	380.65	16/106/6/0
**Huilongguan Siemens**	NA	NA	NA	NA	NA	NA	NA	NA	55/0/11/0
**Huilongguan**	NA	NA	NA	NA	NA	NA	NA	301.28	NA
**Edinburgh**	12.29	13.07	13.07	23.64	NA	24.25	13.58	434.97	0/23/0/3

Supplementary table 3: ENIGMA-Schizophrenia DTI clinical information

ROI	Cohen's d	SE	P-value	CI lowerbound	CI upperbound	i^2	N cases/controls
ACR	-0.38	0.05	$7.9x10^{-14}$	-0.48	-0.28	54.59	1984/2391
ACR_L	-0.34	0.05	$1.8 x10^{-11}$	-0.44	-0.24	54.79	1984/2391
ACR_R	-0.37	0.05	$7.8 x10^{-14}$	-0.47	-0.27	53.29	1984/2391
ALIC	-0.34	0.05	$5.8 x10^{-12}$	-0.44	-0.25	54.13	1984/2391
ALIC_L	-0.33	0.05	$4.4 x10^{-11}$	-0.43	-0.23	53.87	1984/2391
ALIC_R	-0.32	0.05	$7.2 x10^{-10}$	-0.42	-0.22	56.92	1984/2391
AverageFA	-0.40	0.05	$2.2 x10^{-17}$	-0.49	-0.31	48.14	1984/2391
BCC	-0.38	0.05	$2.5 x10^{-16}$	-0.47	-0.29	46.57	1984/2391
CC	-0.38	0.05	$7.1 x10^{-16}$	-0.47	-0.29	48.03	1984/2391
CGC	-0.25	0.05	$2.6 x10^{-07}$	-0.34	-0.15	50.22	1984/2391
CGC_L	-0.21	0.05	$6.6 x10^{-06}$	-0.31	-0.12	49.02	1984/2391
CGC_R	-0.26	0.05	$3.2 x10^{-08}$	-0.35	-0.17	48.98	1984/2391
CGH	-0.10	0.04	$1.8 x10^{-02}$	-0.18	-0.02	35.96	1984/2391
CGH_L	-0.08	0.04	$5.0 x10^{-02}$	-0.17	0.00	38.58	1984/2391
CGH_R	-0.10	0.04	$1.3 x10^{-02}$	-0.17	-0.02	28.16	1984/2391
CR	-0.31	0.04	$5.7 x10^{-13}$	-0.40	-0.23	40.01	1984/2391
CR_L	-0.30	0.04	$1.3 x10^{-11}$	-0.38	-0.21	40.88	1984/2391
CR_R	-0.31	0.04	$3.4 x10^{-12}$	-0.39	-0.22	41.25	1984/2391
CST	-0.04	0.04	$2.7 x10^{-01}$	-0.12	0.03	23.14	1984/2391
CST_L	0.01	0.03	$8.8 x10^{-01}$	-0.06	0.07	9.79	1984/2391
CST_R	-0.09	0.04	$2.9 x10^{-02}$	-0.17	-0.01	28.63	1984/2391
EC	-0.19	0.04	$1.2 x10^{-05}$	-0.28	-0.11	40.88	1984/2391
EC_L	-0.18	0.05	$7.3 x10^{-05}$	-0.27	-0.09	46.33	1984/2391
EC_R	-0.17	0.04	$1.1 x10^{-04}$	-0.26	-0.09	43.93	1984/2391
FX	-0.30	0.04	$1.2 x10^{-11}$	-0.39	-0.22	42.79	1984/2391
FX_ST_L	-0.28	0.04	$6.2 x10^{-12}$	-0.36	-0.20	33.99	1984/2391

FX_ST_R	-0.27	0.04	9.9 x10^{-10}	-0.36	-0.19	42.95	1984/2391
FXST	-0.31	0.04	7.1 x10^{-13}	-0.39	-0.22	37.92	1984/2391
GCC	-0.36	0.04	4.3 x10^{-17}	-0.44	-0.28	37.74	1984/2391
IC	-0.16	0.05	6.0 x10^{-04}	-0.25	-0.07	47.33	1984/2391
IC_L	-0.16	0.05	5.8 x10^{-04}	-0.25	-0.07	46.25	1984/2391
IC_R	-0.15	0.05	3.1 x10^{-03}	-0.24	-0.05	52.82	1984/2391
IFO	-0.10	0.04	9.5 x10^{-03}	-0.18	-0.02	26.03	1984/2391
IFO_L	-0.06	0.04	2.0 x10^{-01}	-0.14	0.03	40.25	1984/2391
IFO_R	-0.11	0.04	1.9 x10^{-03}	-0.18	-0.04	16.80	1984/2391
PCR	-0.24	0.04	6.5 x10^{-10}	-0.31	-0.16	25.58	1984/2391
PCR_L	-0.25	0.04	4.6 x10^{-11}	-0.33	-0.18	25.75	1984/2391
PCR_R	-0.20	0.04	1.3 x10^{-07}	-0.27	-0.12	20.07	1984/2391
PLIC	0.05	0.05	2.5 x10^{-01}	-0.04	0.15	49.48	1984/2391
PLIC_L	0.06	0.05	2.0 x10^{-01}	-0.03	0.15	45.53	1984/2391
PLIC_R	0.04	0.05	3.7 x10^{-01}	-0.05	0.14	53.28	1984/2391
PTR	-0.29	0.04	3.9 x10^{-11}	-0.37	-0.20	39.57	1984/2391
PTR_L	-0.25	0.04	2.3 x10^{-09}	-0.33	-0.17	34.81	1984/2391
PTR_R	-0.27	0.04	6.6 x10^{-11}	-0.35	-0.19	33.45	1984/2391
RLIC	-0.11	0.04	1.1 x10^{-02}	-0.20	-0.03	40.25	1984/2391
RLIC_L	-0.13	0.04	9.0 x10^{-04}	-0.21	-0.05	27.03	1984/2391
RLIC_R	-0.08	0.05	1.2 x10^{-01}	-0.17	0.02	50.91	1984/2391
SCC	-0.20	0.05	2.1 x10^{-04}	-0.30	-0.09	59.30	1984/2391
SCR	-0.14	0.03	3.1 x10^{-05}	-0.21	-0.08	11.24	1984/2391
SCR_L	-0.12	0.03	4.0 x10^{-04}	-0.19	-0.05	10.84	1984/2391
SCR_R	-0.15	0.04	7.2 x10^{-05}	-0.22	-0.08	23.48	1984/2391
SFO	-0.27	0.06	7.3 x10^{-07}	-0.38	-0.17	62.84	1984/2391
SFO_L	-0.26	0.05	1.7 x10^{-08}	-0.35	-0.17	46.55	1984/2391

SFO_R	-0.24	0.06	1.2×10^{-04}	-0.36	-0.12	69.91	1984/2391
SLF	-0.20	0.04	1.3×10^{-06}	-0.28	-0.12	35.17	1984/2391
SLF_L	-0.20	0.04	1.3×10^{-06}	-0.29	-0.12	36.18	1984/2391
SLF_R	-0.18	0.04	1.1×10^{-05}	-0.26	-0.10	31.05	1984/2391
SS	-0.28	0.05	6.2×10^{-10}	-0.37	-0.19	44.32	1984/2391
SS_L	-0.29	0.05	3.7×10^{-09}	-0.39	-0.19	52.65	1984/2391
SS_R	-0.22	0.04	1.9×10^{-08}	-0.30	-0.14	28.06	1984/2391
UNC	-0.16	0.03	2.1×10^{-06}	-0.22	-0.09	5.02	1984/2391
UNC_L	-0.14	0.03	1.6×10^{-05}	-0.20	-0.08	1.15	1984/2391
UNC_R	-0.15	0.03	1.0×10^{-05}	-0.21	-0.08	7.00	1984/2391

Supplementary table 4. Lateralized results for case/control FA differences

ROI	Meta b SANS	Meta b SAPS	SE SANS	SE SAPS	P-value SANS	P value SAPS
ACR	-0.00021	-0.00037	0.00017	0.00031	0.21	0.231
ALIC	-0.00024	-0.00039	0.00010	0.00022	0.01	0.073
Average FA	-0.00018	-0.00018	0.00012	0.00007	0.12	0.009
BCC	-0.00019	-0.00032	0.00019	0.00019	0.33	0.082
CC	-0.00027	-0.00032	0.00011	0.00011	0.01	0.004
CGC	-0.00037	-0.00026	0.00017	0.00013	0.03	0.057
CGH	-0.00024	-0.00007	0.00018	0.00061	0.18	0.907
CR	-0.00017	-0.00019	0.00012	0.00015	0.17	0.186
CST	0.00005	-0.00022	0.00037	0.00015	0.89	0.160
EC	-0.00008	-0.00004	0.00023	0.00009	0.72	0.648
FX	-0.00007	-0.00027	0.00026	0.00026	0.80	0.302
FXST	-0.00009	-0.00016	0.00028	0.00012	0.75	0.186
GCC	-0.00033	-0.00036	0.00012	0.00013	0.01	0.004
IC	-0.00019	-0.00019	0.00008	0.00008	0.02	0.013
IFO	0.00017	-0.00009	0.00015	0.00014	0.27	0.519
PCR	-0.00014	-0.00016	0.00014	0.00015	0.31	0.302
PLIC	-0.00012	-0.00009	0.00010	0.00011	0.25	0.441
PTR	-0.00037	-0.00021	0.00012	0.00015	0.001	0.157
RLIC	-0.00014	-0.00021	0.00023	0.00009	0.54	0.026
SCC	-0.00018	-0.00030	0.00011	0.00012	0.11	0.016
SCR	-0.00006	-0.00003	0.00012	0.00009	0.61	0.740
SFO	-0.00001	-0.00036	0.00031	0.00037	0.97	0.329
SLF	-0.00025	-0.00015	0.00015	0.00010	0.10	0.105
SS	-0.00018	-0.00010	0.00022	0.00010	0.41	0.331

UNC	-0.00042	-0.00030	0.00032	0.00046	0.18	0.517

Supplementary table 5: Partial correlation betas (meta b), and p-values for symptom severity as measured by SAPS and SANS scores

ROI	Cohen's d	Standard error	P-value	95% CI lower-bound	95% CI upper-bound	N female cases/controls
ACR	-0.45	0.053	8.92×10^{-18}	-0.56	-0.35	678/1107
AverageFA	-0.43	0.053	1.52×10^{-16}	-0.54	-0.33	678/1107
CC	-0.41	0.053	3.70×10^{-15}	-0.52	-0.31	678/1107
BCC	-0.40	0.053	7.21×10^{-14}	-0.50	-0.29	678/1107
GCC	-0.39	0.053	1.87×10^{-13}	-0.49	-0.28	678/1107
CR	-0.38	0.053	8.59×10^{-13}	-0.48	-0.27	678/1107
PTR	-0.36	0.053	4.85×10^{-12}	-0.47	-0.26	678/1107
FX	-0.36	0.063	1.33×10^{-8}	-0.48	-0.23	678/1107
ALIC	-0.35	0.053	1.95×10^{-11}	-0.46	-0.25	678/1107
SS	-0.34	0.053	5.76×10^{-11}	-0.45	-0.24	678/1107
FXST	-0.30	0.052	6.69×10^{-9}	-0.41	-0.20	678/1107
PCR	-0.29	0.052	2.30×10^{-8}	-0.40	-0.19	678/1107
SFO	-0.29	0.052	4.57×10^{-8}	-0.39	-0.18	678/1107
SCC	-0.25	0.058	1.50×10^{-5}	-0.37	-0.14	678/1107
SLF	-0.24	0.052	5.22×10^{-6}	-0.34	-0.14	678/1107
UNC	-0.23	0.052	8.12×10^{-6}	-0.34	-0.13	678/1107
EC	-0.23	0.058	8.61×10^{-5}	-0.34	-0.11	678/1107
CGC	-0.23	0.056	4.56×10^{-5}	-0.34	-0.12	678/1107
IC	-0.21	0.052	7.53×10^{-5}	-0.31	-0.10	678/1107
RLIC	-0.18	0.053	0.00	-0.28	-0.07	678/1107
SCR	-0.15	0.052	0.01	-0.25	-0.04	678/1107
IFO	-0.13	0.059	0.03	-0.25	-0.01	678/1107
CGH	-0.13	0.053	0.02	-0.23	-0.02	678/1107
CST	-0.06	0.052	0.24	-0.16	0.04	678/1107
PLIC	0.02	0.052	0.65	-0.08	0.13	678/1107

Supplementary table 6: Female only. Cohen's d effect sizes, standard error (SE), p-values, confidence intervals (CI) after meta-analysis for FA differences between schizophrenia and healthy control females.

ROI	Cohen's d	Standard error	p-value	95% CI-lower bound	95% CI-upper bound	N male cases/controls
AverageFA	-0.40	0.06	3.95×10^{-11}	-0.50	-0.28	1306/1284
BCC	-0.38	0.06	6.47×10^{-10}	-0.49	-0.26	1306/1284
CC	-0.37	0.07	2.64×10^{-09}	-0.50	-0.25	1306/1284
ALIC	-0.37	0.06	4.76×10^{-08}	-0.47	-0.24	1306/1284
GCC	-0.36	0.06	3.89×10^{-10}	-0.47	-0.25	1306/1284
ACR	-0.34	0.06	1.16×10^{-07}	-0.43	-0.22	1306/1284
FXST	-0.32	0.06	4.71×10^{-08}	-0.41	-0.20	1306/1284
CR	-0.29	0.07	1.40×10^{-06}	-0.43	-0.17	1306/1284
SFO	-0.28	0.05	8.79×10^{-05}	-0.38	-0.14	1306/1284
FX	-0.28	0.06	5.28×10^{-08}	-0.39	-0.18	1306/1284
CGC	-0.26	0.05	3.10×10^{-05}	-0.37	-0.14	1306/1284
SS	-0.26	0.06	2.44×10^{-06}	-0.36	-0.15	1306/1284
PTR	-0.24	0.05	2.48×10^{-05}	-0.31	-0.13	1306/1284
PCR	-0.22	0.06	2.65×10^{-06}	-0.31	-0.13	1306/1284
SLF	-0.20	0.06	0.001	-0.32	-0.08	1306/1284
SCC	-0.19	0.06	0.003	-0.28	-0.06	1306/1284
EC	-0.17	0.05	0.002	-0.25	-0.06	1306/1284
SCR	-0.15	0.06	0.004	-0.26	-0.05	1306/1284
IC	-0.14	0.04	0.023	-0.19	-0.02	1306/1284
UNC	-0.11	0.05	0.008	-0.19	-0.03	1306/1284
IFO	-0.10	0.05	0.031	-0.19	-0.01	1306/1284
CGH	-0.08	0.05	0.126	-0.18	0.02	1306/1284
RLIC	-0.08	0.05	0.147	-0.13	0.03	1306/1284
CST	-0.03	0.06	0.458	-0.04	0.06	1306/1284
PLIC	0.07	0.06	0.222	-0.52	0.19	1306/1284

Supplementary table 7: Male only. Cohen's d effect sizes, standard error (SE), p-values, confidence intervals (CI) after meta-analysis for FA differences between schizophrenia and healthy control males.

ROI	Cohen's d	Standard error	meta_pval	95% CI lowerbound	95% CI upperbound	N controls	N cases
Core_FA	0.06	0.09	0.53	-0.124	0.241	1361	1226
Periphery_FA	-0.16	0.08	0.03	-0.309	-0.015	1361	1226
ACR	-0.14	0.07	0.05	-0.284	0.002	1361	1226
ACR_L	-0.14	0.07	0.06	-0.28	0.006	1361	1226
ACR_R	-0.13	0.07	0.08	-0.267	0.016	1361	1226
ALIC	-0.1	0.08	0.22	-0.246	0.055	1361	1226
ALIC_L	-0.13	0.08	0.1	-0.278	0.024	1361	1226
ALIC_R	-0.06	0.08	0.46	-0.216	0.097	1361	1226
BCC	-0.16	0.08	0.03	-0.307	-0.011	1361	1226
CC	-0.13	0.08	0.1	-0.284	0.024	1361	1226
CGC	0.04	0.07	0.59	-0.096	0.168	1361	1226
CGC_L	0.08	0.06	0.23	-0.048	0.201	1361	1226
CGC_R	-0.02	0.07	0.74	-0.158	0.113	1361	1226
CGH	0.1	0.07	0.18	-0.046	0.247	1361	1226
CGH_L	0.11	0.07	0.15	-0.037	0.248	1361	1226

CGH_R	0.07	0.07	0.32	-0.068	0.211	1361	1226
CR	-0.03	0.07	0.74	-0.172	0.122	1361	1226
CR_L	-0.03	0.08	0.66	-0.186	0.117	1361	1226
CR_R	-0.02	0.07	0.82	-0.164	0.13	1361	1226
CST	0.04	0.05	0.42	-0.063	0.151	1361	1226
CST_L	0.09	0.05	0.1	-0.016	0.188	1361	1226
CST_R	-0.01	0.05	0.91	-0.112	0.1	1361	1226
EC	0.09	0.09	0.28	-0.078	0.268	1361	1226
EC_L	0.03	0.08	0.65	-0.117	0.187	1361	1226
EC_R	0.12	0.09	0.17	-0.052	0.299	1361	1226
FX	-0.23	0.07	0.002	-0.377	-0.086	1361	1226
FX_ST_L	-0.14	0.07	0.04	-0.275	-0.008	1361	1226
FX_ST_R	-0.12	0.07	0.1	-0.268	0.025	1361	1226
FXST	-0.15	0.07	0.05	-0.288	-0.003	1361	1226
GCC	-0.15	0.07	0.03	-0.278	-0.012	1361	1226
IC	0.15	0.09	0.09	-0.024	0.331	1361	1226

IC_L	0.11	0.08	0.18	-0.052	0.281	1361	1226
IC_R	0.16	0.1	0.11	-0.033	0.344	1361	1226
IFO	0.06	0.06	0.34	-0.059	0.171	1361	1226
IFO_L	0.05	0.06	0.39	-0.067	0.173	1361	1226
IFO_R	0.04	0.06	0.48	-0.072	0.153	1361	1226
PCR	-0.03	0.06	0.62	-0.159	0.094	1361	1226
PCR_L	-0.05	0.07	0.46	-0.185	0.083	1361	1226
PCR_R	-0.01	0.06	0.83	-0.126	0.101	1361	1226
PLIC	0.25	0.08	0.002	0.092	0.398	1361	1226
PLIC_L	0.23	0.07	0.001	0.097	0.373	1361	1226
PLIC_R	0.22	0.08	0.01	0.054	0.384	1361	1226
PTR	-0.07	0.06	0.29	-0.186	0.055	1361	1226
PTR_L	-0.02	0.06	0.79	-0.132	0.101	1361	1226
PTR_R	-0.1	0.06	0.09	-0.207	0.015	1361	1226
RLIC	0.12	0.07	0.11	-0.026	0.265	1361	1226
RLIC_L	0.08	0.06	0.21	-0.045	0.205	1361	1226

RLIC_R	0.13	0.08	0.12	-0.033	0.296	1361	1226
SCC	-0.01	0.07	0.93	-0.143	0.131	1361	1226
SCR	0.1	0.06	0.11	-0.021	0.215	1361	1226
SCR_L	0.09	0.06	0.14	-0.029	0.201	1361	1226
SCR_R	0.09	0.07	0.18	-0.041	0.222	1361	1226
SFO	-0.08	0.07	0.28	-0.219	0.062	1361	1226
SFO_L	-0.09	0.06	0.15	-0.215	0.034	1361	1226
SFO_R	-0.05	0.08	0.5	-0.199	0.097	1361	1226
SLF	0.12	0.08	0.13	-0.034	0.277	1361	1226
SLF_L	0.09	0.08	0.25	-0.064	0.249	1361	1226
SLF_R	0.12	0.07	0.1	-0.024	0.26	1361	1226
SS	-0.01	0.08	0.85	-0.167	0.138	1361	1226
SS_L	-0.04	0.08	0.63	-0.207	0.124	1361	1226
SS_R	0.01	0.07	0.88	-0.125	0.146	1361	1226
UNC	-0.04	0.05	0.33	-0.133	0.045	1361	1226
UNC_L	-0.02	0.05	0.69	-0.117	0.078	1361	1226

UNC_R	-0.06	0.04	0.16	-0.145	0.023	1361	1226

Supplementary table 8 Lateralized meta-analysis results after covarying for average FA

ROI	Cohen's d	Standard error	P-value	95% CI lowerbound	95% CI upperbound	N controls	N cases
Periphery_FA	-0.32	0.07	$1.61x10^{-6}$	-0.4523	-0.1899	1361	1226
ACR	-0.2	0.07	0.004	-0.3292	-0.0622	1361	1226
ACR_L	-0.18	0.07	0.011	-0.3228	-0.0423	1361	1226
ACR_R	-0.17	0.06	0.007	-0.3006	-0.0472	1361	1226
ALIC	-0.15	0.06	0.013	-0.2752	-0.033	1361	1226
ALIC_L	-0.17	0.06	0.01	-0.2906	-0.0397	1361	1226
ALIC_R	-0.11	0.07	0.096	-0.248	0.0203	1361	1226
AverageFA	-0.33	0.07	$3.14x10^{-6}$	-0.4695	-0.1915	1361	1226
BCC	-0.21	0.07	0.002	-0.3352	-0.0791	1361	1226
CC	-0.19	0.07	0.008	-0.3298	-0.0502	1361	1226
CGC	0.01	0.06	0.81	-0.1036	0.1326	1361	1226
CGC_L	0.05	0.06	0.343	-0.0579	0.1665	1361	1226
CGC_R	-0.04	0.06	0.518	-0.1633	0.0823	1361	1226
CGH	0.05	0.07	0.486	-0.0823	0.173	1361	1226
CGH_L	0.05	0.07	0.417	-0.0749	0.1809	1361	1226

CGH_R	0.03	0.06	0.674	-0.0952	0.1472	1361	1226
CR	-0.08	0.07	0.234	-0.2158	0.0528	1361	1226
CR_L	-0.08	0.08	0.287	-0.2341	0.0693	1361	1226
CR_R	-0.07	0.06	0.255	-0.1954	0.0519	1361	1226
CST	0.02	0.05	0.629	-0.0742	0.1227	1361	1226
CST_L	0.08	0.05	0.099	-0.0144	0.1676	1361	1226
CST_R	-0.03	0.05	0.539	-0.1318	0.0689	1361	1226
EC	0.04	0.07	0.538	-0.0949	0.1819	1361	1226
EC_L	-0.02	0.06	0.721	-0.1464	0.1013	1361	1226
EC_R	0.09	0.08	0.219	-0.0549	0.2395	1361	1226
FX	-0.24	0.07	0.0004	-0.3814	-0.1081	1361	1226
FX_ST_L	-0.2	0.06	0.0003	-0.3088	-0.0907	1361	1226
FX_ST_R	-0.15	0.07	0.025	-0.2825	-0.0187	1361	1226
FXST	-0.2	0.06	0.001	-0.3138	-0.0764	1361	1226
GCC	-0.18	0.07	0.013	-0.3169	-0.037	1361	1226
IC	0.15	0.08	0.054	-0.0023	0.296	1361	1226

IC_L	0.1	0.07	0.148	-0.036	0.2398	1361	1226
IC_R	0.15	0.08	0.072	-0.0133	0.3125	1361	1226
IFO	0.02	0.05	0.704	-0.082	0.1215	1361	1226
IFO_L	0.02	0.06	0.765	-0.0933	0.1269	1361	1226
IFO_R	0.02	0.05	0.752	-0.0835	0.1155	1361	1226
PCR	-0.08	0.05	0.149	-0.1833	0.028	1361	1226
PCR_L	-0.09	0.06	0.139	-0.2007	0.028	1361	1226
PCR_R	-0.06	0.05	0.24	-0.1539	0.0385	1361	1226
PLIC	0.28	0.07	$4.83x10^{-5}$	0.1437	0.4115	1361	1226
PLIC_L	0.26	0.06	$1.46x10^{-5}$	0.1445	0.383	1361	1226
PLIC_R	0.24	0.07	0.001	0.098	0.3891	1361	1226
PTR	-0.1	0.07	0.165	-0.2343	0.0401	1361	1226
PTR_L	-0.04	0.06	0.564	-0.159	0.0866	1361	1226
PTR_R	-0.13	0.06	0.05	-0.2517	-0.0001	1361	1226
RLIC	0.1	0.07	0.127	-0.0286	0.2296	1361	1226
RLIC_L	0.06	0.05	0.306	-0.0512	0.1634	1361	1226

RLIC_R	0.11	0.08	0.132	-0.0345	0.2627	1361	1226
SCC	-0.02	0.07	0.748	-0.1519	0.1091	1361	1226
SCR	0.1	0.06	0.083	-0.0126	0.2048	1361	1226
SCR_L	0.08	0.06	0.151	-0.0306	0.199	1361	1226
SCR_R	0.09	0.06	0.147	-0.0302	0.2013	1361	1226
SFO	-0.07	0.07	0.315	-0.2107	0.0678	1361	1226
SFO_L	-0.09	0.06	0.157	-0.2074	0.0335	1361	1226
SFO_R	-0.04	0.07	0.551	-0.1906	0.1017	1361	1226
SLF	0.1	0.07	0.142	-0.0339	0.2367	1361	1226
SLF_L	0.06	0.07	0.416	-0.0839	0.203	1361	1226
SLF_R	0.1	0.06	0.11	-0.0226	0.2229	1361	1226
SS	-0.06	0.07	0.381	-0.1943	0.0742	1361	1226
SS_L	-0.09	0.08	0.238	-0.2387	0.0592	1361	1226
SS_R	-0.02	0.06	0.689	-0.1452	0.0959	1361	1226
UNC	-0.07	0.04	0.114	-0.1544	0.0165	1361	1226
UNC_L	-0.04	0.05	0.365	-0.1322	0.0486	1361	1226

UNC_R	-0.08	0.04	0.049	-0.1661	-0.0003	1361	1226

Supplementary table 9 Lateralized meta-analysis results after covarying for core FA

ROI	Cohen's d	Standard error	P-value	95% CI lowerbound	95% CI upperbound	N controls	N cases
Core_FA	-0.03	0.08	0.74	-0.19	0.135	1361	1226
ACR	-0.15	0.07	0.03	-0.29	-0.011	1361	1226
ACR_L	-0.14	0.07	0.05	-0.29	-0.002	1361	1226
ACR_R	-0.14	0.07	0.04	-0.28	-0.003	1361	1226
ALIC	-0.12	0.08	0.1	-0.27	0.025	1361	1226
ALIC_L	-0.14	0.07	0.05	-0.29	0.002	1361	1226
ALIC_R	-0.09	0.08	0.23	-0.25	0.059	1361	1226
AverageFA	-0.02	0.08	0.76	-0.18	0.134	1361	1226
BCC	-0.17	0.07	0.02	-0.31	-0.03	1361	1226
CC	-0.14	0.07	0.05	-0.29	0.001	1361	1226
CGC	0.003	0.06	0.97	-0.12	0.129	1361	1226
CGC_L	0.04	0.06	0.49	-0.08	0.163	1361	1226
CGC_R	-0.05	0.07	0.45	-0.18	0.08	1361	1226
CGH	0.09	0.07	0.25	-0.06	0.234	1361	1226
CGH_L	0.09	0.07	0.21	-0.05	0.237	1361	1226

CGH_R	0.06	0.07	0.39	-0.08	0.198	1361	1226
CR	-0.06	0.07	0.38	-0.21	0.079	1361	1226
CR_L	-0.07	0.08	0.37	-0.21	0.08	1361	1226
CR_R	-0.06	0.07	0.41	-0.2	0.082	1361	1226
CST	0.03	0.05	0.59	-0.08	0.136	1361	1226
CST_L	0.08	0.05	0.14	-0.03	0.18	1361	1226
CST_R	-0.02	0.05	0.67	-0.13	0.083	1361	1226
EC	0.06	0.09	0.47	-0.11	0.233	1361	1226
EC_L	0	0.08	0.95	-0.15	0.158	1361	1226
EC_R	0.1	0.09	0.27	-0.07	0.27	1361	1226
FX	-0.22	0.07	0.004	-0.36	-0.075	1361	1226
FX_ST_L	-0.16	0.07	0.02	-0.3	-0.021	1361	1226
FX_ST_R	-0.12	0.07	0.11	-0.26	0.027	1361	1226
FXST	-0.15	0.07	0.04	-0.3	-0.01	1361	1226
GCC	-0.13	0.07	0.06	-0.27	0.007	1361	1226
IC	0.08	0.08	0.31	-0.08	0.247	1361	1226

IC_L	0.06	0.08	0.43	-0.09	0.217	1361	1226
IC_R	0.08	0.09	0.33	-0.09	0.257	1361	1226
IFO	0.04	0.06	0.52	-0.08	0.152	1361	1226
IFO_L	0.03	0.06	0.61	-0.09	0.152	1361	1226
IFO_R	0.03	0.06	0.56	-0.08	0.142	1361	1226
PCR	-0.06	0.06	0.31	-0.19	0.059	1361	1226
PCR_L	-0.07	0.07	0.28	-0.21	0.06	1361	1226
PCR_R	-0.05	0.06	0.39	-0.16	0.061	1361	1226
PLIC	0.21	0.07	0.004	0.07	0.356	1361	1226
PLIC_L	0.21	0.07	0.002	0.07	0.341	1361	1226
PLIC_R	0.18	0.08	0.02	0.03	0.336	1361	1226
PTR	-0.12	0.06	0.03	-0.23	-0.009	1361	1226
PTR_L	-0.07	0.06	0.22	-0.18	0.04	1361	1226
PTR_R	-0.13	0.05	0.01	-0.23	-0.035	1361	1226
RLIC	0.06	0.07	0.45	-0.09	0.197	1361	1226
RLIC_L	0.03	0.06	0.63	-0.1	0.158	1361	1226

RLIC_R	0.07	0.08	0.37	-0.08	0.228	1361	1226
SCC	-0.05	0.07	0.48	-0.18	0.083	1361	1226
SCR	0.05	0.06	0.37	-0.06	0.166	1361	1226
SCR_L	0.04	0.06	0.45	-0.07	0.153	1361	1226
SCR_R	0.05	0.06	0.4	-0.07	0.18	1361	1226
SFO	-0.08	0.07	0.28	-0.22	0.064	1361	1226
SFO_L	-0.1	0.06	0.13	-0.22	0.028	1361	1226
SFO_R	-0.05	0.08	0.51	-0.2	0.099	1361	1226
SLF	0.08	0.08	0.29	-0.07	0.235	1361	1226
SLF_L	0.06	0.08	0.48	-0.1	0.208	1361	1226
SLF_R	0.09	0.07	0.22	-0.05	0.228	1361	1226
SS	-0.07	0.08	0.37	-0.22	0.082	1361	1226
SS_L	-0.09	0.08	0.29	-0.25	0.075	1361	1226
SS_R	-0.04	0.07	0.58	-0.17	0.094	1361	1226
UNC	-0.06	0.04	0.19	-0.15	0.029	1361	1226
UNC_L	-0.03	0.05	0.52	-0.13	0.066	1361	1226

UNC_R	-0.07	0.04	0.08	-0.16	0.009	1361	1226

Supplementary table 10 Lateralized meta-analysis results after covarying for peripheral FA

ROI	Cohen's d	N controls	N patients	N80
AverageFA	-0.40	2391	1984	100
ACR	-0.38	2391	1984	110
BCC	-0.38	2391	1984	110
CC	-0.38	2391	1984	110
GCC	-0.36	2391	1984	123
ALIC	-0.34	2391	1984	137
FXST	-0.31	2391	1984	165
CR	-0.31	2391	1984	165
FX	-0.30	2391	1984	163
SS	-0.28	2391	1984	176
PTR	-0.29	2391	1984	188
SFO	-0.27	2391	1984	217
CGC	-0.25	2391	1984	253
PCR	-0.24	2391	1984	274
SLF	-0.20	2391	1984	394
EC	-0.19	2391	1984	436
SCC	-0.20	2391	1984	394
IC	-0.16	2391	1984	615
UNC	-0.16	2391	1984	615
SCR	-0.14	2391	1984	802
RLIC	-0.11	2391	1984	1299
IFO	-0.10	2391	1984	1571
CGH	-0.10	2391	1984	1571
CST	-0.04	2391	1984	9813
PLIC	0.05	2391	1984	6284

Supplementary table 11. N80: The total number of samples required, per group, to achieve 80% power to detect group differences using a t-test at the threshold of $p<0.05$ (two-tailed).

ROI	meta_d	meta_se	meta_pval	meta_ci.lb	meta_ci.ub	meta_i2
ACR	0.25	0.07	0.0001	0.12	0.38	64.14
ACR_L	0.22	0.06	0.0001	0.11	0.34	53.75
ACR_R	0.24	0.07	0.0002	0.11	0.37	63.20
ALIC	0.10	0.08	0.251	-0.07	0.26	78.58
ALIC_L	0.13	0.08	0.088	-0.02	0.28	73.51
ALIC_R	0.06	0.08	0.469	-0.10	0.22	78.16
AverageFA	0.30	0.10	0.002	0.11	0.48	82.92
BCC	0.30	0.10	0.002	0.11	0.50	84.60
CC	0.34	0.09	0.0002	0.16	0.51	80.71
CGC	0.22	0.08	0.006	0.06	0.38	76.67
CGC_L	0.20	0.08	0.016	0.04	0.36	77.48
CGC_R	0.23	0.08	0.003	0.08	0.38	73.26
CGH	0.05	0.08	0.558	-0.11	0.21	76.46
CGH_L	0.07	0.08	0.398	-0.09	0.22	76.07
CGH_R	0.03	0.08	0.713	-0.12	0.18	73.49
CR	0.27	0.08	0.001	0.11	0.44	78.46
CR_L	0.27	0.08	0.001	0.12	0.43	76.27
CR_R	0.26	0.08	0.002	0.09	0.42	77.98
CST	0.05	0.06	0.400	-0.07	0.18	61.06
CST_L	0.03	0.06	0.594	-0.09	0.16	62.28
CST_R	0.07	0.05	0.229	-0.04	0.17	49.06
EC	0.17	0.09	0.056	0.00	0.34	80.04

EC_L	0.20	0.09	0.017	0.04	0.37	78.98
EC_R	0.10	0.08	0.208	-0.06	0.27	77.87
FX	0.22	0.06	0.001	0.09	0.34	61.49
FX_ST_L	0.23	0.09	0.015	0.04	0.41	82.03
FX_ST_R	0.15	0.09	0.099	-0.03	0.33	81.41
FXST	0.21	0.09	0.026	0.02	0.39	82.52
GCC	0.32	0.07	1.82E-05	0.17	0.46	71.48
IC	0.17	0.10	0.088	-0.03	0.37	84.77
IC_L	0.19	0.10	0.041	0.01	0.38	83.15
IC_R	0.12	0.10	0.218	-0.07	0.32	84.66
IFO	0.11	0.09	0.208	-0.06	0.28	79.33
IFO_L	0.09	0.08	0.239	-0.06	0.24	73.07
IFO_R	0.09	0.08	0.258	-0.07	0.25	75.77
PCR	0.26	0.09	0.003	0.09	0.42	78.85
PCR_L	0.25	0.09	0.004	0.08	0.42	79.77
PCR_R	0.23	0.08	0.003	0.08	0.39	75.05
PLIC	0.10	0.10	0.295	-0.09	0.30	84.45
PLIC_L	0.13	0.09	0.185	-0.06	0.31	82.85
PLIC_R	0.07	0.10	0.464	-0.12	0.26	83.85
PTR	0.19	0.09	0.037	0.01	0.36	80.98
PTR_L	0.13	0.08	0.095	-0.02	0.29	75.70
PTR_R	0.19	0.08	0.017	0.03	0.34	75.39
RLIC	0.22	0.09	0.019	0.04	0.40	82.52
RLIC_L	0.22	0.09	0.014	0.04	0.40	80.82
RLIC_R	0.18	0.09	0.033	0.01	0.35	79.21
SCC	0.24	0.07	0.0006	0.10	0.37	67.29
SCR	0.24	0.10	0.016	0.04	0.44	84.66

SCR_L	0.26	0.09	0.007	0.07	0.44	82.59
SCR_R	0.21	0.10	0.044	0.01	0.41	85.59
SFO	0.17	0.09	0.074	-0.02	0.35	82.53
SFO_L	0.20	0.09	0.027	0.02	0.37	80.13
SFO_R	0.09	0.09	0.291	-0.08	0.27	81.04
SLF	0.20	0.09	0.028	0.02	0.38	81.97
SLF_L	0.21	0.10	0.025	0.03	0.40	83.20
SLF_R	0.18	0.08	0.034	0.01	0.34	77.60
SS	0.20	0.09	0.019	0.03	0.37	79.55
SS_L	0.17	0.09	0.043	0.01	0.34	79.39
SS_R	0.19	0.08	0.014	0.04	0.34	74.30
UNC	0.25	0.08	0.003	0.08	0.41	77.96
UNC_L	0.20	0.09	0.024	0.03	0.37	80.20
UNC_R	0.22	0.07	0.002	0.08	0.36	70.28

Supplementary table 12. Lateralized results for MD differences in 1,954 healthy controls and 1,255 schizophrenia patients

ROI	meta_d	meta_se	meta_pval	meta_ci.lb	meta_ci.ub	meta_i2
ACR	0.34	0.08	$8.89 x 10^{-06}$	0.187	0.483	72.53
ACR_L	0.31	0.07	$2.85 x 10^{-06}$	0.182	0.444	65.01
ACR_R	0.33	0.07	$1.40 x 10^{-05}$	0.178	0.472	72.10
ALIC	0.20	0.08	0.016	0.036	0.357	76.88
ALIC_L	0.21	0.08	0.006	0.060	0.356	72.88
ALIC_R	0.16	0.08	0.053	-0.002	0.318	76.90
AverageFA	0.38	0.10	$8.14 x 10^{-05}$	0.191	0.570	83.31
BCC	0.38	0.09	$4.51 x 10^{-05}$	0.198	0.564	82.18

CC	0.41	0.09	2.76×10^{-06}	0.237	0.578	79.29
CGC	0.23	0.08	0.006	0.065	0.391	77.69
CGC_L	0.19	0.08	0.020	0.031	0.356	77.63
CGC_R	0.24	0.08	0.002	0.088	0.402	75.84
CGH	0.04	0.07	0.515	-0.089	0.178	66.76
CGH_L	0.04	0.07	0.597	-0.098	0.170	66.86
CGH_R	0.05	0.06	0.405	-0.072	0.179	62.23
CR	0.29	0.08	0.000	0.131	0.455	77.33
CR_L	0.29	0.08	0.000	0.132	0.442	75.20
CR_R	0.28	0.08	0.001	0.123	0.442	76.52
CST	0.06	0.06	0.285	-0.053	0.179	55.82
CST_L	0.02	0.06	0.781	-0.102	0.135	57.65
CST_R	0.10	0.06	0.075	-0.010	0.207	49.56
EC	0.23	0.09	0.012	0.049	0.405	81.29
EC_L	0.26	0.09	0.003	0.088	0.424	78.91
EC_R	0.17	0.09	0.058	-0.006	0.336	79.73
FX	0.28	0.05	1.72×10^{-07}	0.174	0.382	45.25
FX_ST_L	0.28	0.08	0.0003	0.128	0.426	73.20
FX_ST_R	0.23	0.07	0.0018	0.086	0.377	71.83
FXST	0.28	0.08	0.0003	0.128	0.430	73.95
GCC	0.37	0.07	1.22×10^{-08}	0.246	0.504	63.75
IC	0.11	0.09	0.2254	-0.065	0.276	79.77
IC_L	0.12	0.08	0.1629	-0.048	0.285	78.72
IC_R	0.09	0.09	0.3110	-0.082	0.256	79.37
IFO	0.12	0.08	0.1041	-0.025	0.270	72.79
IFO_L	0.10	0.07	0.1804	-0.044	0.234	69.31
IFO_R	0.12	0.07	0.0589	-0.005	0.254	64.57

PCR	0.24	0.08	0.0019	0.087	0.387	73.43
PCR_L	0.24	0.08	0.0030	0.081	0.395	75.85
PCR_R	0.22	0.07	0.0016	0.082	0.348	66.45
PLIC	-0.02	0.08	0.7951	-0.185	0.142	77.95
PLIC_L	-0.01	0.08	0.8819	-0.174	0.149	77.35
PLIC_R	-0.02	0.08	0.7727	-0.181	0.135	76.39
PTR	0.26	0.08	0.0016	0.099	0.422	77.23
PTR_L	0.21	0.08	0.0088	0.053	0.366	75.80
PTR_R	0.25	0.07	0.0001	0.124	0.384	64.65
RLIC	0.12	0.07	0.0861	-0.017	0.264	69.95
RLIC_L	0.13	0.07	0.0636	-0.007	0.270	69.09
RLIC_R	0.10	0.07	0.1487	-0.035	0.229	65.74
SCC	0.26	0.07	0.0003	0.119	0.397	69.19
SCR	0.17	0.09	0.0489	0.001	0.348	80.36
SCR_L	0.17	0.08	0.0474	0.002	0.328	77.77
SCR_R	0.17	0.09	0.0595	-0.007	0.351	81.57
SFO	0.23	0.09	0.0076	0.061	0.399	79.22
SFO_L	0.22	0.08	0.0053	0.067	0.383	76.22
SFO_R	0.17	0.08	0.0378	0.010	0.338	78.09
SLF	0.22	0.09	0.0129	0.046	0.392	80.16
SLF_L	0.22	0.09	0.0176	0.038	0.398	81.74
SLF_R	0.20	0.08	0.0122	0.044	0.358	76.01
SS	0.21	0.08	0.0078	0.057	0.372	76.08
SS_L	0.22	0.08	0.0107	0.050	0.381	78.39
SS_R	0.17	0.07	0.0135	0.036	0.311	68.62
UNC	0.26	0.07	0.0004	0.117	0.402	70.75
UNC_L	0.21	0.08	0.0074	0.056	0.365	75.01

Supplementary table 13. Lateralized results for RD differences in 1,956 healthy controls and 1,252 schizophrenia patients

ADNI 3-month	N=19 (baseline, 3 months, 6 months)		
ROI (FA)	**ICC**	**CI lowerbound**	**CI upperbound**
ACR	0.989	0.976	0.995
ALIC	0.981	0.96	0.992
Average_FA	0.986	0.967	0.995
BCC	0.977	0.948	0.99
CC	0.979	0.95	0.991
CGC	0.926	0.843	0.969
CGH	0.941	0.871	0.976
CR	0.992	0.983	0.997
CST	0.947	0.886	0.978
EC	0.982	0.963	0.993
FX	0.954	0.902	0.981
FXST	0.962	0.919	0.984
GCC	0.974	0.945	0.989
IC	0.983	0.965	0.993
IFO	0.951	0.893	0.979
PCR	0.993	0.985	0.997
PLIC	0.95	0.893	0.979
PTR	0.989	0.976	0.995
RLIC	0.987	0.97	0.995
SCC	0.959	0.909	0.983
SCR	0.991	0.98	0.996
SFO	0.972	0.94	0.988
SLF	0.985	0.966	0.994
SS	0.987	0.972	0.994

UNC	0.988	0.973	0.995

Supplementary table 14: Intraclass correlation coefficients of FA measures for 19 healthy individuals scanned at baseline, 3 months and 6 months follow-up.

ADNI 6-month	N=19 (baseline, 3 months, 6 months)		
ROI (AD)	**ICC**	**CI lowerbound**	**CI upperbound**
ACR	0.957	0.908	0.982
ALIC	0.987	0.972	0.995
Average_FA	0.99	0.979	0.996
BCC	0.976	0.948	0.99
CC	0.978	0.954	0.991
CGC	0.956	0.906	0.982
CGH	0.952	0.897	0.98
CR	0.988	0.975	0.995
CST	0.938	0.868	0.974
EC	0.992	0.982	0.997
FX	0.978	0.953	0.991
FXST	0.956	0.907	0.982
GCC	0.969	0.934	0.987
IC	0.984	0.965	0.993
IFO	0.982	0.962	0.993
PCR	0.996	0.991	0.998
PLIC	0.97	0.936	0.988
PTR	0.987	0.973	0.995

RLIC	0.978	0.953	0.991
SCC	0.979	0.955	0.991
SCR	0.993	0.984	0.997
SFO	0.989	0.977	0.996
SLF	0.991	0.982	0.996
SS	0.979	0.956	0.991
UNC	0.991	0.98	0.996

Supplementary table 15: Intraclass correlation coefficients of AD (axial diffusivity) measures for 19 healthy individuals scanned at baseline, 3 months and 6 months follow-up.

ADNI 6-month v 3-month	N=19 (baseline, 3 months, 6 months)		
ROI (RD)	**ICC**	**CI lowerbound**	**CI upperbound**
ACR	0.986	0.97	0.994
ALIC	0.993	0.984	0.997
Average_FA	0.992	0.983	0.997
BCC	0.993	0.984	0.997
CC	0.99	0.974	0.996
CGC	0.981	0.958	0.992
CGH	0.968	0.932	0.987
CR	0.993	0.985	0.997
CST	0.892	0.766	0.955
EC	0.994	0.987	0.997
FX	0.983	0.963	0.993
FXST	0.971	0.938	0.988
GCC	0.983	0.96	0.993

IC	0.991	0.981	0.996
IFO	0.954	0.902	0.981
PCR	0.997	0.993	0.999
PLIC	0.965	0.925	0.985
PTR	0.991	0.981	0.996
RLIC	0.99	0.979	0.996
SCC	0.973	0.94	0.989
SCR	0.992	0.982	0.997
SFO	0.988	0.975	0.995
SLF	0.994	0.987	0.997
SS	0.992	0.983	0.997
UNC	0.993	0.985	0.997

Supplementary table 16: Intraclass correlation coefficients of RD (radial diffusivity) measures for 19 healthy individuals scanned at baseline, 3 months and 6 months follow-up.

ADNI 6-month v 3-month	N=19 (baseline, 3 months, 6 months)		
ROI (MD)	**ICC**	**CI lowerbound**	**CI upperbound**
ACR	0.96	0.914	0.983
ALIC	0.991	0.982	0.996
Average_FA	0.966	0.928	0.986
BCC	0.957	0.908	0.982
CC	0.944	0.881	0.977
CGC	0.957	0.908	0.982
CGH	0.945	0.884	0.977
CR	0.983	0.964	0.993

CST	0.854	0.687	0.939
EC	0.99	0.979	0.996
FX	0.757	0.475	0.899
FXST	0.757	0.475	0.899
GCC	0.963	0.921	0.985
IC	0.987	0.973	0.995
IFO	0.964	0.923	0.985
PCR	0.996	0.991	0.998
PLIC	0.969	0.934	0.987
PTR	0.974	0.945	0.989
RLIC	0.979	0.956	0.991
SCC	0.913	0.815	0.964
SCR	0.712	0.138	0.899
SFO	0.983	0.963	0.993
SLF	0.983	0.963	0.993
SS	0.979	0.955	0.991
UNC	0.991	0.98	0.996

Supplementary table 17: Intraclass correlation coefficients of MD (mean diffusivity) measures for 19 healthy individuals scanned at baseline, 3 months and 6 months follow-up.

ADNI 3-month	N=19 (baseline, 3 months)		
ROI (FA)	**ICC**	**CI lowerbound**	**CI upperbound**
ACR	0.981	0.95	0.992
ALIC	0.978	0.944	0.991
Average_FA	0.978	0.941	0.992

BCC	0.967	0.906	0.988
CC	0.964	0.894	0.987
CGC	0.905	0.759	0.963
CGH	0.931	0.797	0.975
CR	0.987	0.962	0.995
CST	0.88	0.688	0.954
EC	0.968	0.918	0.988
FX	0.925	0.805	0.971
FXST	0.937	0.836	0.976
GCC	0.952	0.876	0.981
IC	0.98	0.949	0.992
IFO	0.948	0.864	0.98
PCR	0.988	0.963	0.995
PLIC	0.951	0.873	0.981
PTR	0.978	0.943	0.991
RLIC	0.978	0.944	0.991
SCC	0.913	0.763	0.967
SCR	0.982	0.954	0.993
SFO	0.978	0.943	0.991
SLF	0.975	0.923	0.991
SS	0.981	0.951	0.993
UNC	0.974	0.914	0.991

Supplementary table 18: Intraclass correlation coefficients of FA measures for 19 healthy individuals scanned at baseline and 3 months.

ADNI 3-month	N=19 (baseline, 3 months)		
ROI (AD)	**ICC**	**CI-lowerbound**	**CI-upperbound**
ACR	0.954	0.88	0.982
ALIC	0.978	0.944	0.982
Average_FA	0.987	0.966	0.995
BCC	0.96	0.898	0.985
CC	0.972	0.927	0.989
CGC	0.902	0.749	0.962
CGH	0.932	0.803	0.975
CR	0.99	0.975	0.996
CST	0.891	0.719	0.958
EC	0.99	0.974	0.996
FX	0.972	0.921	0.99
FXST	0.934	0.831	0.974
GCC	0.964	0.909	0.986
IC	0.973	0.93	0.99
IFO	0.973	0.93	0.99
PCR	0.995	0.986	0.998
PLIC	0.95	0.872	0.981
PTR	0.982	0.954	0.993
RLIC	0.965	0.91	0.986
SCC	0.972	0.929	0.989
SCR	0.993	0.982	0.997
SFO	0.978	0.943	0.992
SLF	0.985	0.962	0.994
SS	0.972	0.929	0.989

UNC	0.985	0.962	0.994

Supplementary table 19: Intraclass correlation coefficients of AD (axial diffusivity) measures for 19 healthy individuals scanned at baseline and 3 months.

ADNI 3-month	N=19 (baseline, 3 months)		
ROI (MD)	**ICC**	**CI lowerbound**	**CI upperbound**
ACR	0.938	0.839	0.976
ALIC	0.984	0.959	0.994
Average_FA	0.947	0.861	0.979
BCC	0.915	0.779	0.968
CC	0.904	0.75	0.963
CGC	0.93	0.817	0.973
CGH	0.927	0.815	0.972
CR	0.977	0.94	0.991
CST	0.797	0.479	0.922
EC	0.986	0.965	0.995
FX	0.419	-0.589	0.781
FXST	0.958	0.892	0.984
GCC	0.943	0.854	0.978
IC	0.984	0.958	0.994
IFO	0.96	0.896	0.984
PCR	0.997	0.993	0.999
PLIC	0.964	0.908	0.986
PTR	0.962	0.901	0.985

RLIC	0.964	0.909	0.986
SCC	0.893	0.721	0.959
SCR	0.973	0.93	0.99
SFO	0.969	0.919	0.988
SLF	0.971	0.925	0.989
SS	0.968	0.918	0.988
UNC	0.982	0.955	0.993

Supplementary table 20: Intraclass correlation coefficients of MD (mean diffusivity) measures for 19 healthy individuals scanned at baseline and 3 months.

ADNI 3-month	N=19 (baseline, 3 months)		
ROI (RD)	**ICC**	**CI lowerbound**	**CI upperbound**
ACR	0.981	0.952	0.993
ALIC	0.986	0.964	0.994
Average_FA	0.988	0.97	0.995
BCC	0.99	0.973	0.996
CC	0.983	0.95	0.994
CGC	0.971	0.927	0.989
CGH	0.962	0.902	0.985
CR	0.989	0.97	0.996
CST	0.811	0.503	0.927
EC	0.991	0.978	0.997
FX	0.976	0.939	0.991
FXST	0.966	0.913	0.987
GCC	0.975	0.936	0.99

IC	0.988	0.97	0.995
IFO	0.955	0.884	0.983
PCR	0.995	0.986	0.998
PLIC	0.962	0.903	0.985
PTR	0.982	0.954	0.993
RLIC	0.983	0.956	0.994
SCC	0.94	0.838	0.977
SCR	0.983	0.956	0.994
SFO	0.98	0.949	0.992
SLF	0.988	0.969	0.995
SS	0.99	0.973	0.996
UNC	0.985	0.958	0.994

Supplementary table 21: Intraclass correlation coefficients of RD (radial diffusivity) measures for 19 healthy individuals scanned at baseline and 3 months.

ADNI 6-month	N=19 (baseline, 6 months)		
ROI (FA)	**ICC**	**CI lowerbound**	**CI upperbound**
ACR	0.986	0.95	0.995
ALIC	0.976	0.934	0.991
Average_FA	0.977	0.855	0.993
BCC	0.964	0.878	0.988
CC	0.969	0.816	0.991
CGC	0.85	0.618	0.942
CGH	0.923	0.704	0.974
CR	0.988	0.964	0.996

CST	0.949	0.871	0.98
EC	0.968	0.919	0.988
FX	0.923	0.805	0.97
FXST	0.932	0.828	0.974
GCC	0.966	0.898	0.988
IC	0.975	0.937	0.991
IFO	0.934	0.816	0.975
PCR	0.99	0.971	0.996
PLIC	0.91	0.766	0.965
PTR	0.985	0.959	0.994
RLIC	0.984	0.885	0.995
SCC	0.935	0.787	0.977
SCR	0.988	0.971	0.996
SFO	0.947	0.864	0.98
SLF	0.973	0.918	0.99
SS	0.98	0.946	0.992
UNC	0.98	0.948	0.992

Supplementary table 22: Intraclass correlation coefficients of FA measures for 19 healthy individuals scanned at baseline and 6 months.

ADNI 3-month	N=19 (baseline, 6 months)		
ROI (AD)	**ICC**	**CI-lowerbound**	**CI-upperbound**
ACR	0.91	0.766	0.965
ALIC	0.977	0.94	0.991
Average_FA	0.979	0.947	0.992

BCC	0.972	0.929	0.989
CC	0.966	0.912	0.987
CGC	0.94	0.845	0.977
CGH	0.935	0.834	0.975
CR	0.975	0.936	0.99
CST	0.912	0.769	0.966
EC	0.985	0.961	0.994
FX	0.967	0.907	0.988
FXST	0.93	0.82	0.973
GCC	0.948	0.867	0.98
IC	0.977	0.939	0.991
IFO	0.974	0.932	0.99
PCR	0.992	0.977	0.997
PLIC	0.957	0.89	0.983
PTR	0.978	0.945	0.992
RLIC	0.966	0.913	0.987
SCC	0.952	0.877	0.981
SCR	0.985	0.959	0.995
SFO	0.978	0.945	0.992
SLF	0.984	0.958	0.994
SS	0.963	0.904	0.986
UNC	0.983	0.957	0.993

Supplementary table 23: Intraclass correlation coefficients of AD (axial diffusivity) measures for 19 healthy individuals scanned at baseline and 3 months.

ADNI 3-month	N=19 (baseline, 6 months)	

ROI (MD)	ICC	CI lowerbound	CI upperbound
ACR	0.959	0.895	0.984
ALIC	0.986	0.965	0.995
Average_FA	0.982	0.954	0.993
BCC	0.988	0.97	0.996
CC	0.978	0.929	0.992
CGC	0.961	0.894	0.985
CGH	0.955	0.883	0.982
CR	0.984	0.956	0.994
CST	0.848	0.602	0.942
EC	0.988	0.97	0.995
FX	0.979	0.922	0.993
FXST	0.933	0.828	0.974
GCC	0.965	0.894	0.987
IC	0.982	0.955	0.993
IFO	0.939	0.845	0.976
PCR	0.994	0.981	0.998
PLIC	0.941	0.847	0.977
PTR	0.983	0.957	0.993
RLIC	0.981	0.95	0.993
SCC	0.946	0.844	0.98
SCR	0.987	0.962	0.995
SFO	0.989	0.971	0.996
SLF	0.99	0.975	0.996
SS	0.983	0.956	0.993
UNC	0.987	0.965	0.995

Supplementary table 24: Intraclass correlation coefficients of MD (mean diffusivity) measures for 19 healthy individuals scanned at baseline and 3 months.

ADNI 3-month	N=19 (baseline, 6 months)		
ROI (RD)	**ICC**	**CI lowerbound**	**CI upperbound**
ACR	0.977	0.937	0.991
ALIC	0.99	0.972	0.996
Average_FA	0.982	0.949	0.994
BCC	0.987	0.945	0.996
CC	0.98	0.875	0.994
CGC	0.959	0.869	0.985
CGH	0.958	0.892	0.984
CR	0.987	0.959	0.995
CST	0.827	0.549	0.934
EC	0.989	0.971	0.996
FX	0.98	0.925	0.993
FXST	0.935	0.835	0.975
GCC	0.972	0.87	0.991
IC	0.983	0.956	0.993
IFO	0.919	0.794	0.969
PCR	0.994	0.982	0.998
PLIC	0.924	0.801	0.971
PTR	0.985	0.962	0.994
RLIC	0.985	0.962	0.994

SCC	0.945	0.823	0.981
SCR	0.987	0.965	0.995
SFO	0.987	0.966	0.995
SLF	0.99	0.974	0.996
SS	0.988	0.969	0.995
UNC	0.988	0.967	0.995

Supplementary table 25: Intraclass correlation coefficients of RD (radial diffusivity) measures for 19 healthy individuals scanned at baseline and 3 months.

ADNI 6-month v 3-month	N=19 (3 months, 6months)		
ROI (FA)	**ICC**	**CI lowerbound**	**CI upperbound**
ACR	0.984	0.958	0.994
ALIC	0.961	0.899	0.985
Average_FA	0.985	0.96	0.994
BCC	0.964	0.908	0.986
CC	0.972	0.928	0.989
CGC	0.919	0.792	0.969
CGH	0.889	0.713	0.957
CR	0.991	0.976	0.996
CST	0.94	0.844	0.977
EC	0.985	0.961	0.994
FX	0.949	0.869	0.98
FXST	0.964	0.908	0.986
GCC	0.968	0.919	0.988
IC	0.97	0.922	0.988

IFO	0.899	0.724	0.962
PCR	0.992	0.98	0.997
PLIC	0.918	0.788	0.968
PTR	0.986	0.965	0.995
RLIC	0.981	0.947	0.993
SCC	0.966	0.913	0.987
SCR	0.988	0.97	0.995
SFO	0.945	0.859	0.979
SLF	0.985	0.961	0.994
SS	0.979	0.948	0.992
UNC	0.992	0.979	0.997

Supplementary table 26: Intraclass correlation coefficients of FA measures for 19 healthy individuals scanned at 3 months and 6 months after baseline.

ADNI 3-month	N=19 (3 months, 6months)		
ROI (AD)	**ICC**	**CI-lowerbound**	**CI-upperbound**
ACR	0.95	0.871	0.981
ALIC	0.986	0.965	0.995
Average_FA	0.99	0.974	0.996
BCC	0.958	0.891	0.984
CC	0.958	0.891	0.984
CGC	0.963	0.905	0.986
CGH	0.922	0.802	0.97
CR	0.983	0.957	0.993
CST	0.927	0.813	0.972

EC	0.988	0.968	0.995
FX	0.964	0.906	0.986
FXST	0.945	0.823	0.981
GCC	0.952	0.877	0.981
IC	0.978	0.942	0.991
IFO	0.974	0.933	0.99
PCR	0.994	0.986	0.998
PLIC	0.961	0.897	0.985
PTR	0.982	0.955	0.993
RLIC	0.973	0.931	0.99
SCC	0.981	0.952	0.993
SCR	0.989	0.973	0.996
SFO	0.995	0.987	0.998
SLF	0.993	0.981	0.997
SS	0.974	0.932	0.99
UNC	0.99	0.975	0.996

Supplementary table 27: Intraclass correlation coefficients of AD (axial diffusivity) measures for 19 healthy individuals scanned at 3 months and 6 months after baseline.

ADNI 3-month	N=19 (3 months, 6months)		
ROI (MD)	**ICC**	**CI lowerbound**	**CI upperbound**
ACR	0.927	0.809	0.972
ALIC	0.991	0.977	0.997
Average_FA	0.918	0.787	0.968
BCC	0.896	0.73	0.96

CC	0.862	0.642	0.947
CGC	0.915	0.779	0.967
CGH	0.879	0.687	0.954
CR	0.965	0.909	0.987
CST	0.734	0.309	0.897
EC	0.981	0.952	0.993
FX	0.36	-0.661	0.754
FXST	0.94	0.845	0.977
GCC	0.927	0.811	0.972
IC	0.977	0.94	0.991
IFO	0.941	0.847	0.977
PCR	0.991	0.977	0.997
PLIC	0.956	0.886	0.983
PTR	0.938	0.84	0.976
RLIC	0.965	0.909	0.986
SCC	0.772	0.408	0.912
SCR	0.966	0.911	0.987
SFO	0.964	0.908	0.986
SLF	0.959	0.893	0.984
SS	0.957	0.888	0.983
UNC	0.988	0.969	0.995

Supplementary table 28: Intraclass correlation coefficients of MD (mean diffusivity) measures for 19 healthy individuals scanned at 3 months and 6 months after baseline.

ADNI 3-month	N=19 (3 months, 6months)		
ROI (RD)	**ICC**	**CI lowerbound**	**CI upperbound**
ACR	0.979	0.947	0.992
ALIC	0.991	0.976	0.996
Average_FA	0.995	0.985	0.998
BCC	0.992	0.979	0.997
CC	0.993	0.982	0.998
CGC	0.986	0.952	0.995
CGH	0.937	0.838	0.976
CR	0.994	0.986	0.998
CST	0.892	0.718	0.959
EC	0.991	0.978	0.997
FX	0.967	0.916	0.987
FXST	0.97	0.923	0.988
GCC	0.976	0.937	0.991
IC	0.988	0.969	0.995
IFO	0.919	0.794	0.969
PCR	0.998	0.995	0.999
PLIC	0.96	0.896	0.984
PTR	0.992	0.978	0.997
RLIC	0.988	0.966	0.995
SCC	0.991	0.976	0.996
SCR	0.992	0.979	0.997
SFO	0.981	0.952	0.993
SLF	0.994	0.984	0.998
SS	0.986	0.964	0.995

UNC	0.995	0.988	0.998

Supplementary table 29: Intraclass correlation coefficients of RD (radial diffusivity) measures for 19 healthy individuals scanned at 3 months and 6 months after baseline.

(baseline, 24 hours, 72 hours)			
ROI (FA)	**ICC**	**CI-lowerbound**	**CI-upperbound**
ACR	0.932	0.801	0.982
ALIC	0.849	0.576	0.959
AverageFA	0.974	0.926	0.993
BCC	0.864	0.618	0.963
CC	0.877	0.654	0.966
CGC	0.898	0.711	0.972
CGH	0.744	0.309	0.929
CR	0.904	0.727	0.974
CST	0.75	0.304	0.931
EC	0.892	0.697	0.971
FX	0.681	0.076	0.914
FXST	0.809	0.461	0.948
GCC	0.924	0.784	0.979
IC	0.825	0.513	0.952
IFO	0.877	0.645	0.967

PCR	0.873	0.644	0.965
PLIC	0.799	0.434	0.945
PTR	0.919	0.771	0.978
RLIC	0.886	0.679	0.969
SCC	0.895	0.704	0.971
SCR	0.899	0.715	0.972
SFO	0.626	0.046	0.893
SLF	0.902	0.723	0.973
SS	0.934	0.812	0.982
UNC	0.956	0.876	0.988

Supplementary table 30: Intraclass correlation coefficients for FA measures of N=10 healthy individuals, age 22-28 years, scanned at baseline, 24 hours and 72 hours

(baseline, 24 hours)			
ROI (FA)	**ICC**	**CI-lowerbound**	**CI-upperbound**
ACR	0.934	0.738	0.983
ALIC	0.881	0.515	0.971
AverageFA	0.962	0.846	0.991
BCC	0.867	0.478	0.967
CC	0.885	0.53	0.971
CGC	0.937	0.739	0.984
CGH	0.754	-0.078	0.94
CR	0.937	0.755	0.984
CST	0.917	0.676	0.979
EC	0.933	0.727	0.984
FX	0.142	-3.532	0.799

FXST	0.683	-0.324	0.925
GCC	0.903	0.598	0.976
IC	0.889	0.538	0.973
IFO	0.833	0.29	0.959
PCR	0.932	0.725	0.983
PLIC	0.92	0.669	0.98
PTR	0.959	0.841	0.99
RLIC	0.893	0.557	0.974
SCC	0.924	0.691	0.981
SCR	0.939	0.765	0.985
SFO	0.813	0.312	0.952
SLF	0.926	0.696	0.982
SS	0.946	0.779	0.987
UNC	0.965	0.864	0.991

Supplementary table 31: Intraclass correlation coefficients for FA measures of N=10 healthy individuals, age 22-28 years, scanned at baseline and 24 hours .

(baseline, 72 hours)			
ROI (FA)	**ICC**	**CI-lowerbound**	**CI-upperbound**
ACR	0.876	0.459	0.97
ALIC	0.774	0.151	0.943
AverageFA	0.987	0.921	0.997
BCC	0.819	0.331	0.954
CC	0.838	0.397	0.959
CGC	0.869	0.47	0.968
CGH	0.656	-0.15	0.91

CR	0.824	0.326	0.956
CST	0.625	-0.378	0.905
EC	0.827	0.361	0.956
FX	0.768	0.107	0.942
FXST	0.802	0.276	0.95
GCC	0.896	0.611	0.974
IC	0.72	0.016	0.928
IFO	0.888	0.58	0.972
PCR	0.824	0.353	0.955
PLIC	0.605	-0.439	0.899
PTR	0.872	0.524	0.968
RLIC	0.86	0.473	0.965
SCC	0.859	0.476	0.964
SCR	0.805	0.277	0.951
SFO	0.506	-0.37	0.862
SLF	0.885	0.142	0.939
SS	0.896	0.607	0.974
UNC	0.956	0.827	0.989

Supplementary table 32: Intraclass correlation coefficients for FA measures of N=10 healthy individuals, age 22-28 years, scanned at baseline and 72 hours .

(24 hours, 72 hours)			
ROI (FA)	**ICC**	**CI-lowerbound**	**CI-upperbound**
ACR	0.898	0.589	0.975
ALIC	0.719	0.018	0.927
AverageFA	0.933	0.746	0.983

BCC	0.758	0.068	0.939
CC	0.772	0.135	0.942
CGC	0.764	0.14	0.94
CGH	0.612	-0.246	0.897
CR	0.831	0.378	0.957
CST	0.486	-0.769	0.867
EC	0.786	0.222	0.946
FX	0.498	-0.878	0.873
FXST	0.69	-0.187	0.922
GCC	0.871	0.518	0.967
IC	0.691	-0.092	0.921
IFO	0.746	-0.006	0.937
PCR	0.741	0.022	0.935
PLIC	0.662	-0.227	0.914
PTR	0.81	0.239	0.953
RLIC	0.779	0.195	0.944
SCC	0.788	0.185	0.947
SCR	0.824	0.35	0.955
SFO	0.301	-1.408	0.819
SLF	0.763	0.142	0.939
SS	0.876	0.532	0.969
UNC	0.88	0.55	0.97

Supplementary table 33: Intraclass correlation coefficients for FA measures of N=10 healthy individuals, age 22-28 years, scanned at 24 hours and 72 hours after baseline.
